# Evidence of Cosmic Impact at Abu Hureyra, Syria at the Younger Dryas Onset (~12.8 ka): High-temperature melting at >2200 °C

**DOI:** 10.1038/s41598-020-60867-w

**Published:** 2020-03-06

**Authors:** Andrew M. T. Moore, James P. Kennett, William M. Napier, Ted E. Bunch, James C. Weaver, Malcolm LeCompte, A. Victor Adedeji, Paul Hackley, Gunther Kletetschka, Robert E. Hermes, James H. Wittke, Joshua J. Razink, Michael W. Gaultois, Allen West

**Affiliations:** 1 College of Liberal Arts, Rochester Institute of Technology, Rochester, NY 14623 USA; 2 Department of Earth Science and Marine Science Institute, University of California Santa Barbara, Santa Barbara, CA 93106 USA; 3 Armagh Observatory and Planetarium, College Hill, Armagh BT61 9DG, Northern Ireland, UK; 4 Geology Division, School of Earth and Sustainability, Northern Arizona University, Flagstaff, AZ 86011 USA; 5 Wyss Institute for Biologically Inspired Engineering, Harvard University, Cambridge, MA 02138 USA; 6 Elizabeth City State University, Center of Excellence in Remote Sensing Education and Research, Elizabeth City, NC, 27909 USA; 7 Department of Natural Sciences, Elizabeth City State University, Elizabeth City, NC 27909 USA; 8 U.S. Geological Survey (USGS), 12201 Sunrise Valley Drive, Reston, VA, 20192, USA; 9 Institute of Geology, Czech Academy of Science of the Czech Republic and, Charles University, Faculty of Science, Czech Republic, CZE; and University of Alaska Fairbanks, 903 Koyukuk Drive, Fairbanks, Alaska, 99775 USA; 10 Los Alamos National Laboratory (retired), White Rock, NM 87547 USA; 11 Center for Advanced Materials Characterization at Oregon (CAMCOR), University of Oregon, Eugene, OR 97403 USA; 12 Leverhulme Research Centre for Functional Materials Design, The Materials Innovation Factory, Department of Chemistry, University of Liverpool, Liverpool, UK; 13 Comet Research Group, 2204 Lakewood Drive, Prescott, AZ, 86301 USA

**Keywords:** Astronomy and astrophysics, Geochemistry

## Abstract

At Abu Hureyra (AH), Syria, the 12,800-year-old Younger Dryas boundary layer (YDB) contains peak abundances in meltglass, nanodiamonds, microspherules, and charcoal. AH meltglass comprises 1.6 wt.% of bulk sediment, and crossed polarizers indicate that the meltglass is isotropic. High YDB concentrations of iridium, platinum, nickel, and cobalt suggest mixing of melted local sediment with small quantities of meteoritic material. Approximately 40% of AH glass display carbon-infused, siliceous plant imprints that laboratory experiments show formed at a minimum of 1200°–1300 °C; however, reflectance-inferred temperatures for the encapsulated carbon were lower by up to 1000 °C. Alternately, melted grains of quartz, chromferide, and magnetite in AH glass suggest exposure to minimum temperatures of 1720 °C ranging to >2200 °C. This argues against formation of AH meltglass in thatched hut fires at 1100°–1200 °C, and low values of remanent magnetism indicate the meltglass was not created by lightning. Low meltglass water content (0.02–0.05% H_2_O) is consistent with a formation process similar to that of tektites and inconsistent with volcanism and anthropogenesis. The wide range of evidence supports the hypothesis that a cosmic event occurred at Abu Hureyra ~12,800 years ago, coeval with impacts that deposited high-temperature meltglass, melted microspherules, and/or platinum at other YDB sites on four continents.

## Introduction

Firestone *et al*.^1^ first proposed that a cosmic impact event occurred ~12,800 years ago^1,2^, resulting in multi-continental airbursts, possibly caused by the debris stream from a short-period comet^1,3,4^. This event is proposed to have created the Younger Dryas boundary layer (YDB), which contains peak abundances of magnetic spherules^1,5–11^, meltglass^7,8^, carbon spherules^1,12^, glasslike carbon^1,12^, charcoal^13,14^, platinum^15–18^, iridium^17,18^, nickel^19^ cobalt^19^, and/or nanodiamonds^12,20–22^ at ~40 sites across North America and Europe, including from Abu Hureyra, Syria (Appendix, Fig. S1). In this paper, the term “airburst/impact” refers to a collision of a cosmic body with the Earth’s atmosphere, after which numerous smaller fragments may strike the ground forming transient surface craters. These studies propose that such an impact event triggered a cascade of secondary effects, including a brief impact winter and severe Younger Dryas (YD) climate change (span: ~12,800–11,500 cal BP)^1,13,14^, along with possible contributions to the megafaunal extinctions and human population declines^23^. Moore and Kennett^24^ concluded that impact-triggered climate change caused the prehistoric villagers at Abu Hureyra to transition from hunting/gathering to cultivation, indicative of earliest agriculture, one of the most significant cultural transformations in human history.

Since the introduction of the hypothesis, there have been many scientific publications supporting it and some against it^10,25–29^. It is beyond the scope of this study to present them again in detail, and so, for in-depth details and references, see Pino *et al*.^9^, Supporting Information, and Wolbach *et al*.^13^, Appendix, Table A3.

At Abu Hureyra, Bunch *et al*.^8^ investigated large quantities of Abu Hureyra meltglass (called here “AH glass”) and identified high-temperature minerals, such as corundum (Al_2_O_3_, melting point at ~2044 °C), mullite (3Al_2_O_3_·2SiO_2_ at ~1840 °C), and suessite (Fe_3_Si at ~2300 °C). The latter mineral is rare on Earth but common in meteorites, suggesting a cosmic connection. Those researchers proposed that the melted minerals indicate the village was destroyed by a cosmic airburst/impact event. They also found similar coeval meltglass in YDB strata at Melrose, Pennsylvania and Blackville, South Carolina, which are ~10,000 km away. The most distant YDB site containing high-temperature melted evidence is at Pilauco, Chile^9^, 14,000 km away or ~35% of Earth’s circumference.

Arguing against the results of Bunch *et al*.^8^, Thy *et al*.^30^ examined meltglass from Abu Hureyra and several other sites in northern Syria, conducted heating experiments, and concluded that AH glass resulted from thatched hut fires that melted AH sediment at no more than ~1200 °C (Appendix, Text S1**)**. However, Bunch *et al*.^8^ reported melted high-temperature minerals on the outer surfaces and vesicles of Abu Hureyra meltglass, whereas Thy *et al*.^30^ only examined the interiors of meltglass, where viscous, molten glass can act as an insulator. Because the maximum temperatures inferred by Thy *et al*.^30^ differ from those of Bunch *et al*.^8^, we re-examine here AH glass formation temperatures and explore multiple potential origins.

This study addresses the following. (i) What are the maximum temperatures and potential origins of YDB meltglass and mineral inclusions? (ii) Can reflectance values for YDB charcoal and carbon spherules be used to infer accurate formation temperatures? (iii) Can measurements of remanent magnetization be used to infer potential formation processes of AH glass and spherules? (iv) Are the characteristics of Abu Hureyra meltglass similar or dissimilar to other high-temperature materials, including meltglass produced by known cosmic impact events, volcanism, anthropogenesis, lightning, and biomass burning?

## Site Setting, Geology, and Age

**Figure 1:**
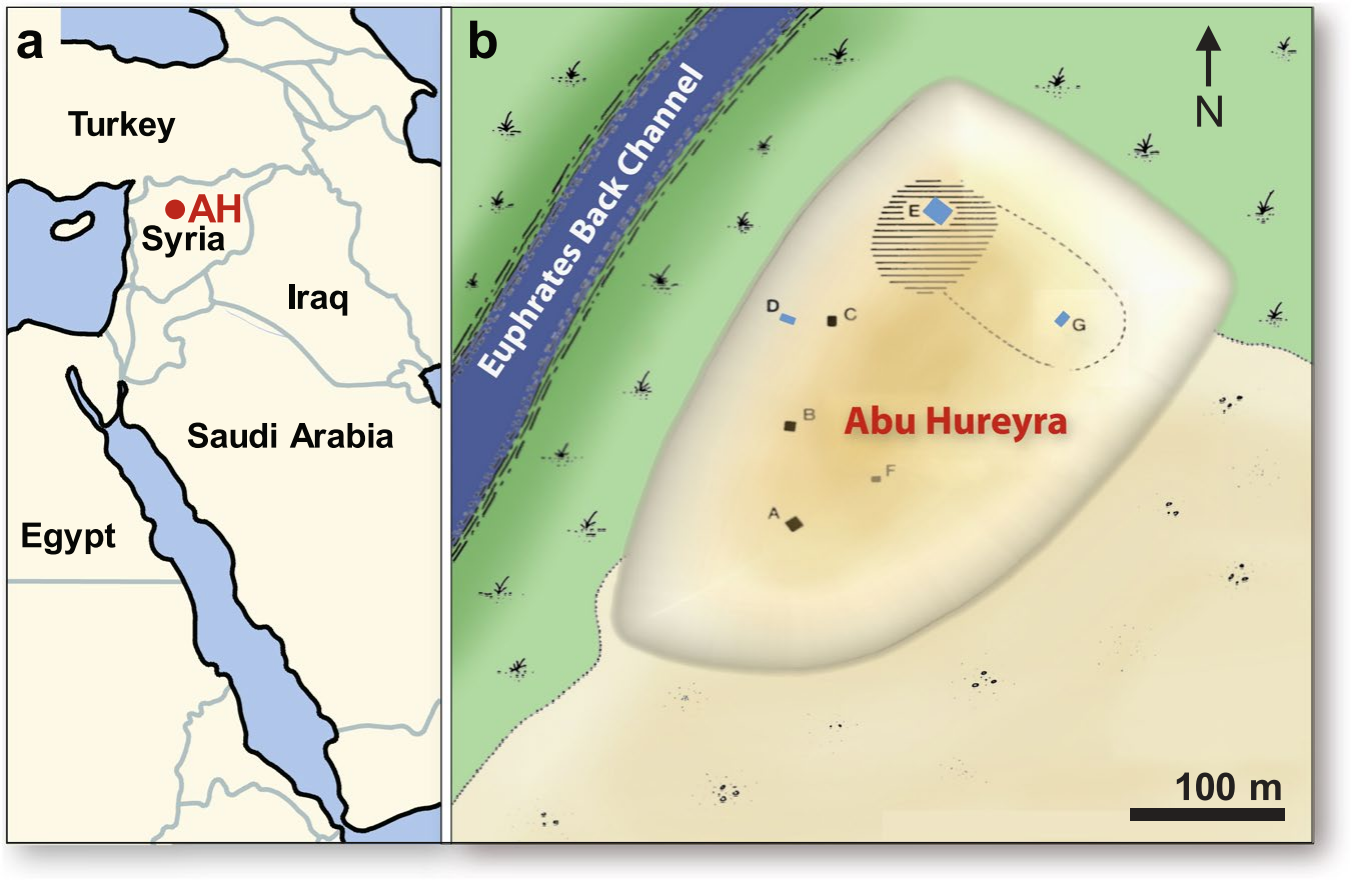
Location of Abu Hureyra (adapted from Moore *et al*.^31^. (**a**) Map of the Middle East, showing Abu Hureyra location (AH) in Syria. (**b**) Map of the Abu Hureyra tell, showing locations of excavation trenches labeled A-G near a back channel of Euphrates River that is now abandoned. Sediment samples from Trenches D, E, and G (blue rectangles) contain abundance peaks in YDB proxies, including spherules^5^, nanodiamonds^12^, meltglass^8^, and platinum.

Abu Hureyra is a mound settlement (commonly known as a “tell”) located in northern Syria along the Euphrates River (Fig. 1a). When the sediments used in this study were excavated in 1972 and 1973, the site was located on the southwest bank of the Euphrates floodplain. After the river was dammed, the site was flooded and now lies beneath Lake Assad. The bedrock beneath the village consists predominantly of chalky limestone with thin beds of fine-grained chert and marls that date to the Middle and Upper Eocene^31^ and is typically covered with sandy Quaternary alluvium. Excavations in the village exposed charcoal-rich, living surfaces (Appendix, Fig. S2) that contain meltglass, melted Fe- and Si-rich spherules, nanodiamonds, and carbon spherules. These proxy-rich surfaces have a Bayesian-modeled YDB age of 12825 ± 55 cal BP (range: 12935–12705). Impact-related materials were observed in 3 dated excavation trenches (D, E, and G) separated by up to 175 m (Fig. 1b, Appendix, Tables S1 and S2).

## Results and Interpretations

These investigations into Abu Hureyra meltglass (AH glass) included analyses of sedimentary abundance peaks, morphologies, geochemical composition, crystallinity, melting/boiling temperatures, carbon reflectance, remanent magnetism, and water content. These were accomplished using reflected-light microscopy, scanning electron microscopy (SEM) with energy-dispersive spectroscopy (EDS), electron microprobe, reflectance, and transmission Fourier transform infrared spectroscopy (FTIR). Numbers, depths, ages of stratigraphic levels, and impact proxies investigated are listed in Appendix, Table S2.

### Abu Hureyra meltglass

#### Morphology and composition

Peak concentrations of AH meltglass^8^, magnetic spherules^5^, and nanodiamonds^12^ co-occur in and adjacent to the E301 sample at a depth below the surface of ~405 cm (Appendix, Fig. S1). Representative SEM-EDS analyses for AH meltglass are listed in Appendix, Table S3, keyed to figures in this paper; representative microprobe analyses for AH glass and other types of meltglass are listed in Appendix, Table S4.

**Figure 2:**
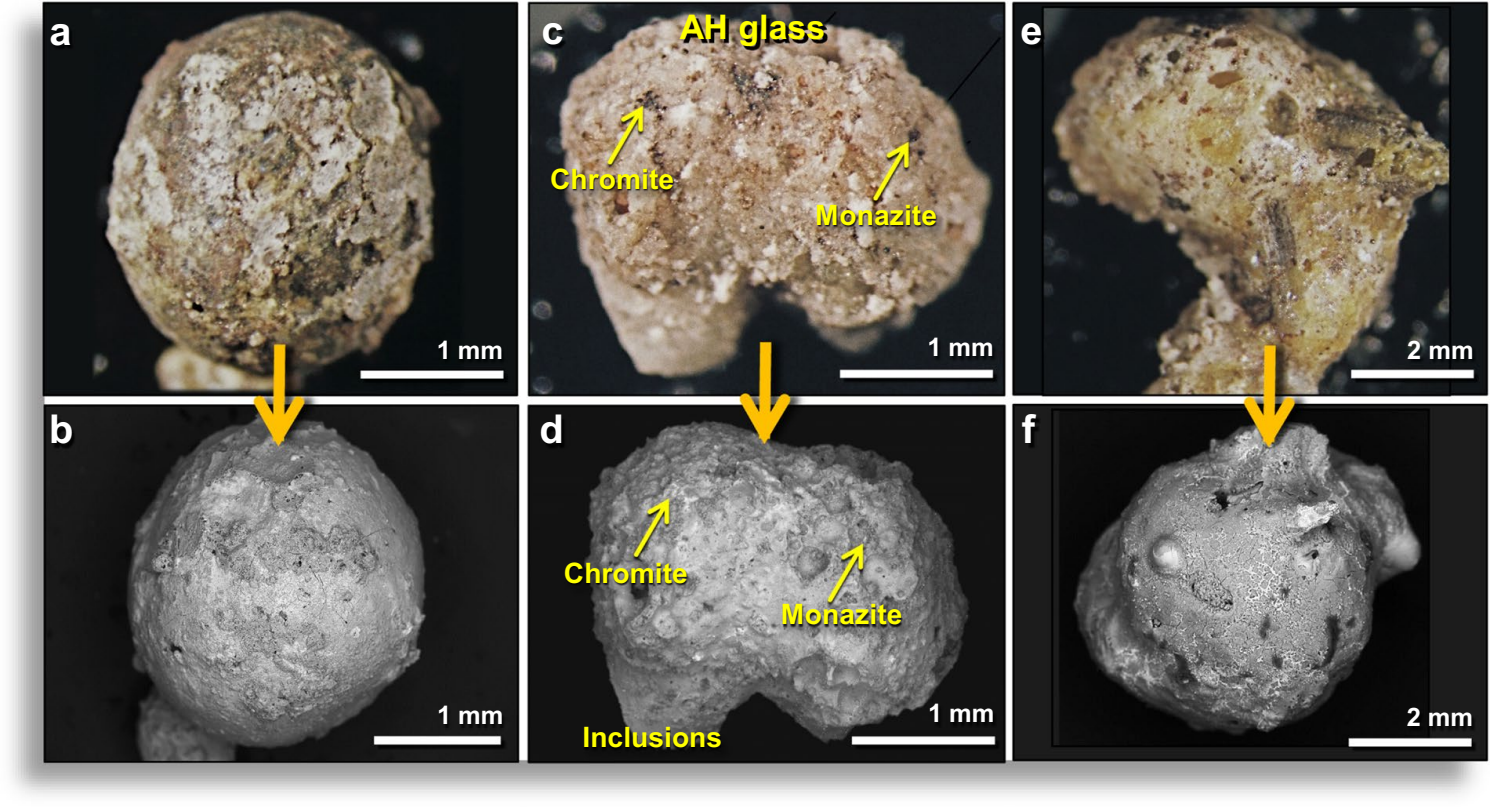
Typical examples of Abu Hureyra (AH) meltglass. (**a**) Photomicrograph of rough 2.5-mm-wide AH glass spheroid from level 457, sample E313, 413 cm depth. The term “level 457” refers to a single stratum as numbered by the original site excavators^31^. “Sample E313” was extracted from level 457 at 413 cm below a surveyed datum point. Numbers, depths, and ages of stratigraphic levels investigated are listed in Appendix, Tables S1 and S2. Sediment samples discussed in this article are from Trench E unless otherwise noted. (**b**) SEM image of the same glass fragment as in (**a**). (**c**) Photomicrograph of a 2.7-mm-wide fragment from level 449, sample E305, 412 cm depth. Yellow arrows point to inclusions of chromite and monazite. (**d**) SEM image of the same AH glass fragment as in (**c**). (**e**) Photomicrograph of 7.8-mm glass fragment from level 457, sample E313 at a depth of 413 cm depth. (**f**) SEM image of a different view of the same glass fragment as in (**e**).

**Figure 3:**
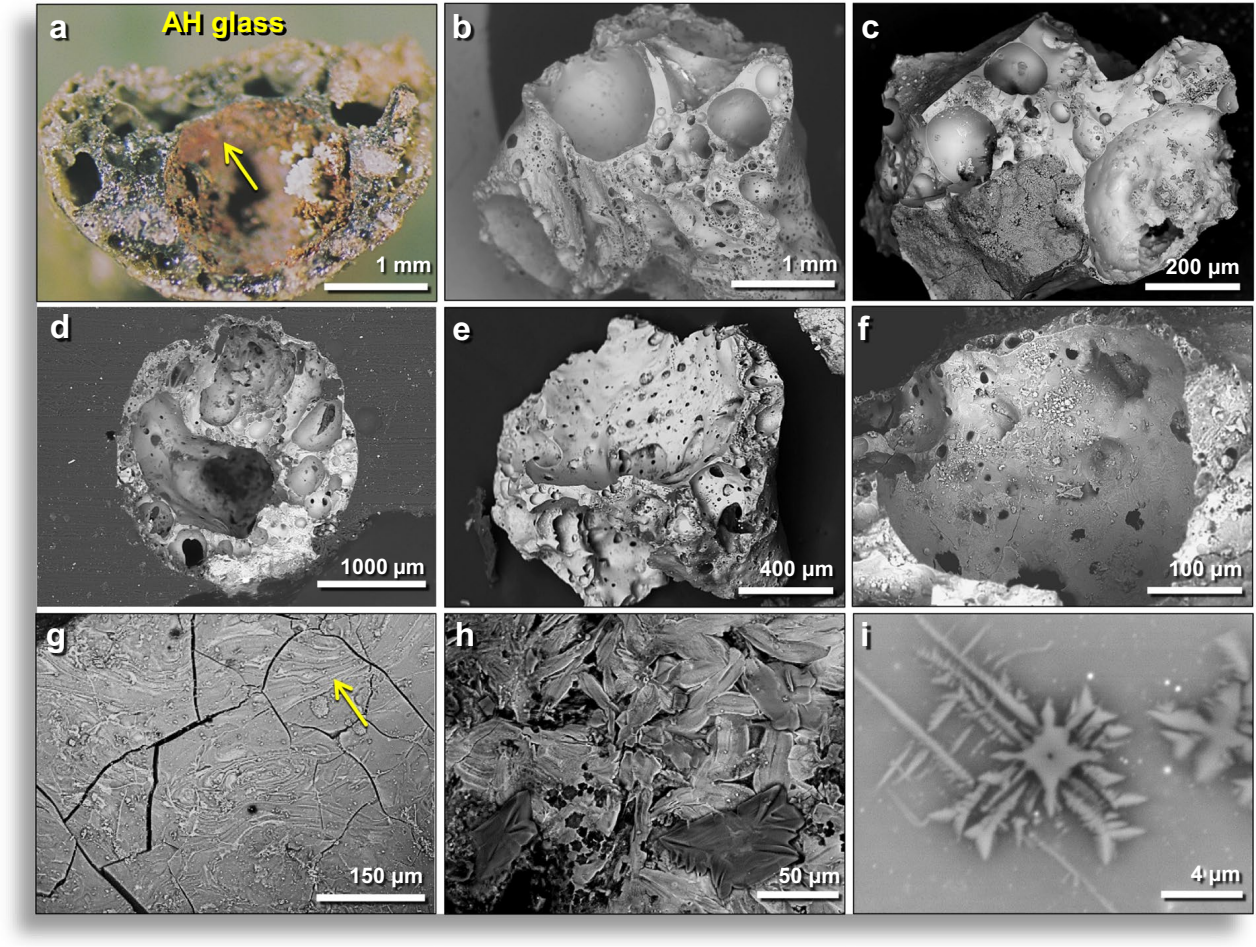
Examples of inclusions inside vesicles and on outer surfaces of AH glass. (**a**) Photomicrograph of 3.9-mm fragment of broken meltglass dominated by a large vesicle. The red-brown color (arrow) indicates the lining of vesicle contains high Fe content. (**b–f**) SEM images of the inner walls of typical glass vesicles. (**g**) SEM image of flow textures (e.g., at arrow) inside a large vesicle. (**h**) Common crystalline textures inside glass vesicles formed as the molten glass cooled. (**i**) Central, cross-shaped, ~5.5-μm-wide embedded crystal composed of Ca, Mg, and Si. Based on SEM-EDS, it likely is a quenched, skeletal diopside crystal (CaMgSi_2_O_6_) within a glassy Ca-Al-Si matrix. (**a,f,g**) from level 457, sample E313, 413 cm depth. (**b**–**e**,**h**–**i**) from level 445, sample E301, 405 cm depth.

Meltglass fragments comprise 1.6 wt.% of bulk sediment and range in diameter from <200 μm to ~1.4 cm, with most ranging from 1–2 mm in diameter. Some AH glass fragments are roughly spherical, others are rounded (Fig. 2a–b), and some have complex subrounded shapes (Fig. 2c–f); most typically are found broken and rarely whole. Glass surfaces are generally rough-textured but commonly have small areas with smooth, reflective surfaces. Nearly all fragments larger than several millimeters are highly vesicular (Fig. 3), so that most float on water, which is how they were extracted from AH sediment^31^. Nearly all vesicles observed in broken AH glass display interior surfaces with one or more of the following features: (i) flow patterns suggesting the material was in motion when it cooled; (ii) small inclusions of melted-to-unmelted minerals, including quartz, magnetite, titanomagnetite, chrome-magnetite, chromite, zircon, and monazite (Figs. 2, 3**)**; and/or (iii) intricate, well-organized, crystalline patterns (Fig. 3). Optical microscopy typically shows that the mineral linings have different colors than the outside of AH glass fragments. Often, these linings are red-colored, indicative of high-Fe content.

Bunch *et al*.^8^ found that most AH glass is a calcium-rich aluminosilicate mix, with an average composition of 59.5 wt.% SiO_2_, 15.2 wt.% CaO, 6.3 wt.% FeO, and 6.0 wt.% Al_2_O_3_, with the remaining 13.0 wt.% as various minor oxides. We report similar compositions of AH glass averaging 50.9 wt.% SiO_2_, 16.6 wt.% CaO, 9.8 wt.% FeO, 9.0 wt.% Al_2_O_3_ and the remaining 13.7 wt.% as various minor oxides, excluding carbon. The meltglass is geochemically similar to the local sediment, which is mostly composed of plagioclase, carbonates, gypsum, chert, and quartz with other minor rock components derived from local and upstream bedrock. The HCl treatment of several grams of AH sediment left less than 50% of the original volume due to carbonate dissolution, as indicated by vigorous outgassing. When carbon was measured by SEM-EDS along with oxides, we found typical C concentrations to fall between 25 and 50 wt.%, consistent with high carbonate content.

Crossed polarizers are useful in determining whether a mineral is anisotropic (e.g., quartz, calcite, and tourmaline) or isotropic, a characteristic that occurs in only a few materials (e.g, sodium chloride, anthropogenic glass, tektites, and some impact glass^32^). Six pieces of AH glass were crushed, and dozens of the thinnest fragments were viewed through crossed polarizers, which revealed that all were isotropic, consistent with being amorphous glass. Fragments of YDB glass samples of an Australasian tektite (Muong Nong) were also isotropic, as were samples from two other YDB sites at Melrose, Pennsylvania, and Blackville, South Carolina.

### Furnace and torch heating experiments

To test the hypothesis that local melting produced AH glass, heating experiments were conducted on AH sediment and pieces of excavated AH glass. Laboratory furnaces were used at temperatures varying from 1100° to 1850 °C at 50–150° intervals (Table S5). All experiments used either sediment from Abu Hureyra level 435, sample ES15 at 395 cm depth or YDB AH glass displaying plant imprints (e.g., impressions of leaves and stems) from level 457, sample E313 at 413 cm depth. The highest temperature of 1850 °C achieved in the heating experiments is known to occur in impact events and atomic detonations but does not occur under any other natural terrestrial conditions except lightning strikes.

#### Melting plant-imprinted AH glass

Baseline temperatures were established by heating fragments of 12,800-year-old, plant-imprinted AH glass until they fully melted. At ~1100 °C, the glass softened but did not completely melt, retaining most of its original morphology. Plant imprints remained visible but became less pronounced. Thirteen original fragments melted within a range of 1200°–1300 °C. It is important to note that this is the minimum temperature to which they may have been exposed, not the maximum.

#### Melting AH bulk sediment

At 1100 °C, the bulk sediment showed no obvious melting. From 1200° to 1300 °C, the melted portion of AH sediment encapsulated abundant unmelted or partially melted mineral grains (Appendix, Fig. S3). Smaller quartz grains (approximately <50 µm) began partial melting at ~1300 °C, most likely because limestone (CaCO_3_), soda ash (Na_2_CO_3_), and other minerals acted as fluxing agents to lower the melting point of quartz. At 1700 °C, clusters of white minerals (identified by SEM-EDS as mostly quartz) remained visible on the solid AH glass surface, while nearly all of the remaining sediment and grains were transformed to transparent-to-translucent amber-brown-colored glass.

In summary, when bulk AH sediment was heated to ~1700 °C, the resulting meltglass had similar morphology and characteristics as AH glass. When cooled from ~1700 °C, this glass remained molten until it solidified at 1200° to 1300 °C. The same was true of actual pieces of AH glass. These results show this as the solidus temperature for both AH glass and laboratory meltglass produced from AH sediment, closely matching that reported by Thy *et al*.^30^. However, this is not the maximum temperature range possible, as claimed by Thy *et al*.^30^, but rather, the minimum temperature at which molten AH sediment solidifies. These laboratory experiments conclusively show that it is possible for AH glass to reach temperatures to ~1700 °C, much higher than its melting point of ~1200° to 1300 °C.

### AH meltglass with carbon-rich siliceous plant imprints

**Figure 4:**
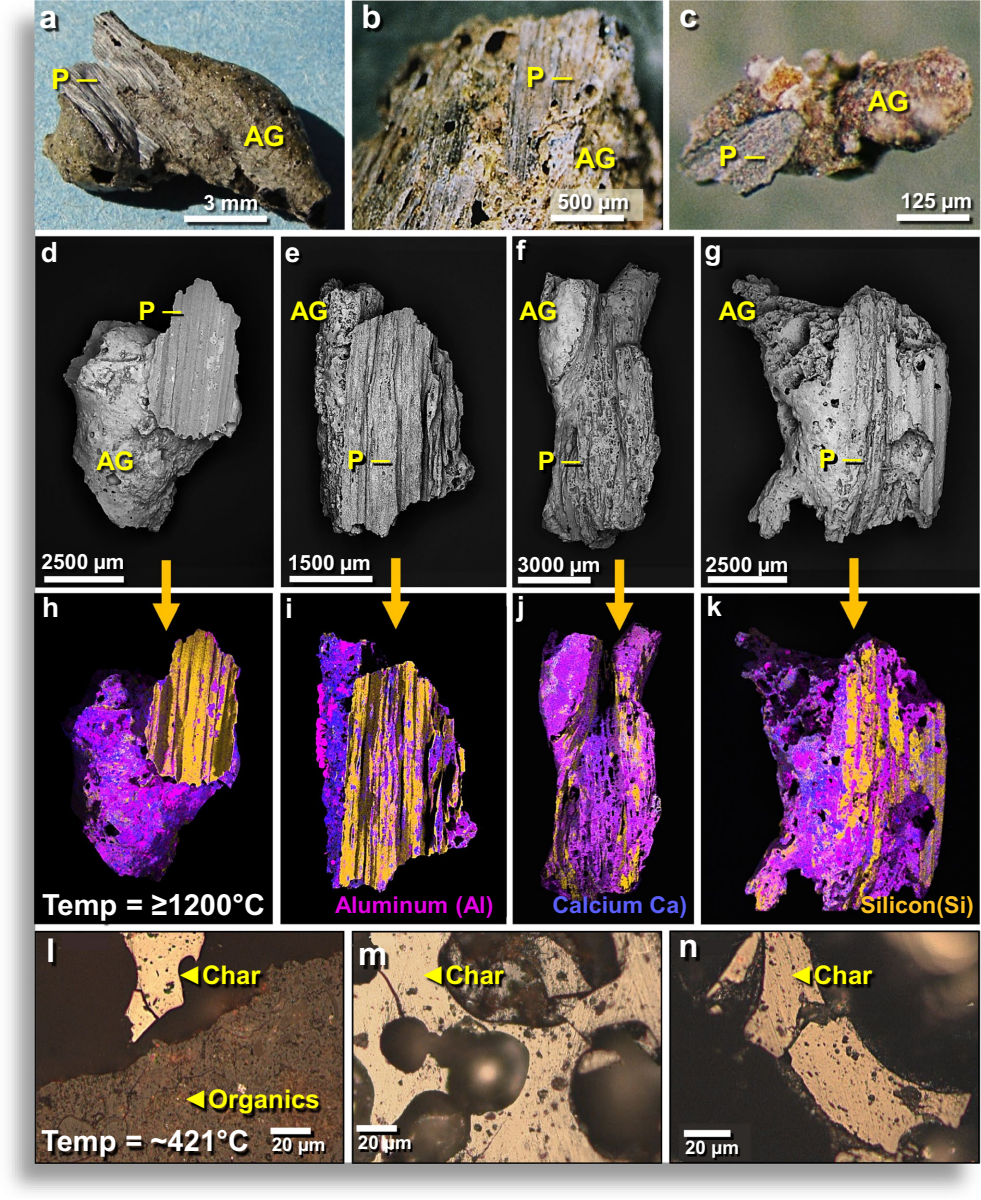
Plant imprints on surfaces of AH glass. (**a–c**) Photomicrographs of AH glass (AG) with silicified plant imprints (P). (**d–k**) SEM images of carbon-infused, siliceous plant imprints (P) on AH glass (AG). (**i–m**) Multi-element EDS maps of same objects in images above. Magenta represents Al, Blue represents Ca, and Gold represents Si. Carbon is not shown, but SEM-EDS indicates it is almost exclusively associated with the Si-rich imprints. From level 457, sample E313, 413 cm depth. (**l–n**) SEM images of polished charred organics embedded within AH glass. The reflectance-derived temperature is ~421 °C, much lower than the laboratory-measured melting point. AH meltglass from level 445, sample E301, 405 cm. Inferred temperature range of ~1200° to 1300 °C for melting AH glass.

Approximately 40% of AH meltglass pieces display one or more carbon-infused impressions, typically appearing as fine, parallel ridges and grooves (Fig. 4a–k**)** that closely correspond to the ribbed patterns on plant stems and leaves. This plant-imprinted AH glass typically contains inclusions of charcoal and burned carbon, all indicative of biomass burning closely associated with the formation of AH meltglass. Such plant imprints (with C, O, Mg, Al, Si) have been previously reported by Schultz *et al*.^33^, who found that temperatures above 1500 °C were necessary to entrap organics and that lower temperatures resulted in carbonization.

SEM-EDS analyses indicate the imprints have distinct chemistry, averaging ~80 wt.% SiO_2_ (range: 65–100 wt.%) with an average of ~20 wt.% carbon (range: 0–35 wt.%). The composition of non-imprinted AH glass is distinctly different, mainly containing a mix of oxides of Si, Ca, Fe, Al, and Mg, with only a few weight percent of carbon. SEM analyses reveal a three-layered morphology: (i) Ca-rich glass as the matrix; (ii) fused with microns-thick silicified plant material, typically only on one surface; and (iii) with a transition layer between the two.

This morphology may best be explained as resulting when airborne molten Ca-rich glass struck and fused to local plant material and then cooled to preserve the plant imprints. This carbon-infused meltglass is associated with melted magnetic spherules^5^ and nanodiamonds^12^, along with abundance peaks in nickel, cobalt, iridium, and platinum, as discussed below.

#### Reflectance as a temperature proxy

We investigated whether carbon infused into plant-imprinted glass might reveal clues to formation temperatures. To do so, we measured reflectance (%R_0_), defined as the percentage of vertically incident monochromatic light reflected from a highly polished surface of a sample of carbon calibrated against the light reflected from a carbon standard of known reflectance^34,35^. Typically, carbon exposed to higher temperatures exhibits higher reflectance values. We determined the average reflectance-derived temperatures for two types of AH carbon: for carbon embedded in AH meltglass from level 445, the reflectance-inferred temperature was ~421 °C (Fig. 4l–m); for loose sedimentary charcoal found associated with AH meltglass, the temperature was ~391 °C. For carbon inside the AH glass, the inferred reflectance-derived temperatures are ~779° to 879 °C lower than the measured laboratory melt temperature range of ~1200° to 1300 °C. This important result demonstrates that melted glass can maintain higher temperatures than that of the charcoal it encloses (Appendix, Table S6). All experimental results for reflectance are shown in Appendix, Figs. S4–S6 and described in Appendix, Text S2.

### Silicon-rich minerals as temperature indicators

**Figure 5:**
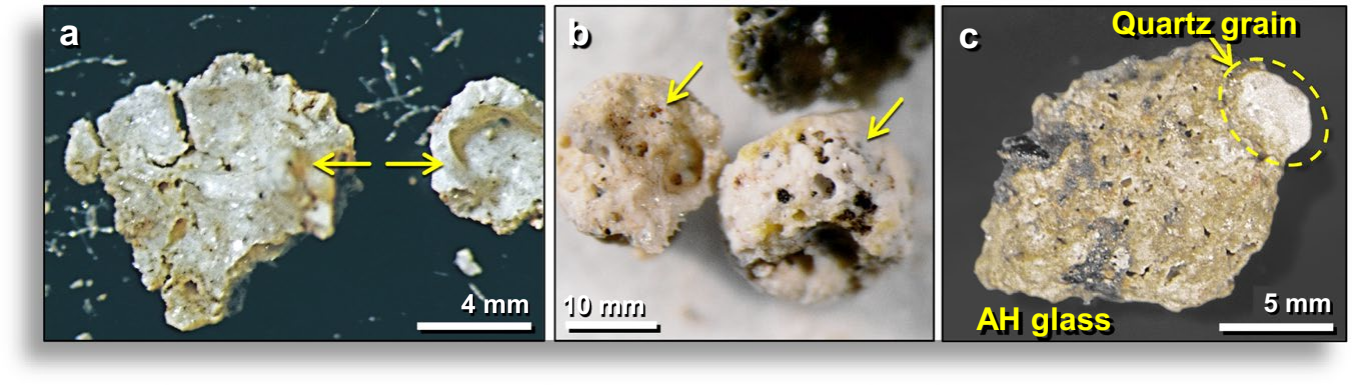
Photomicrographs of varieties of SiO_2_ on surfaces of AH glass. (**a**) ~12.5-mm-wide fragment of AH glass with a thin coating of SiO_2_ (lechatelierite) that lines bubble cavities. The AH glass matrix is pale green, and the Si-rich coating is white. Under light microscopy, the surface is highly reflective and mostly vesicle-free with few inclusions. (**b**) Rounded, vesicular, rough-textured, 21-mm-wide AH glass fragment composed of SiO_2_ (white areas) with some inclusions. (**c**) Dashed circles and arrows indicate partially melted quartz grains embedded in AH glass. (Equilibrium melting point of quartz is 1720 °C, with a flux-adjusted melting point of ~1520 °C. (**a**) from level 457, sample E313, 413 cm depth. (**b**–**c**) from level 445, sample E301, 405 cm depth.

Light microscopy and SEM-EDS analyses show that AH glass commonly contains several varieties of SiO_2_: (i) thin coatings of vitreous or glassy amorphous SiO_2,_ often called lechatelierite (Fig. 5a); (ii) subrounded melted glass objects that are nearly 100 wt.% SiO_2_ (Fig. 5b); and (iii) partially to fully melted quartz grains (Fig. 5c).

### Melted amorphous silica (SiO_2_ as lechatelierite)

**Figure 6:**
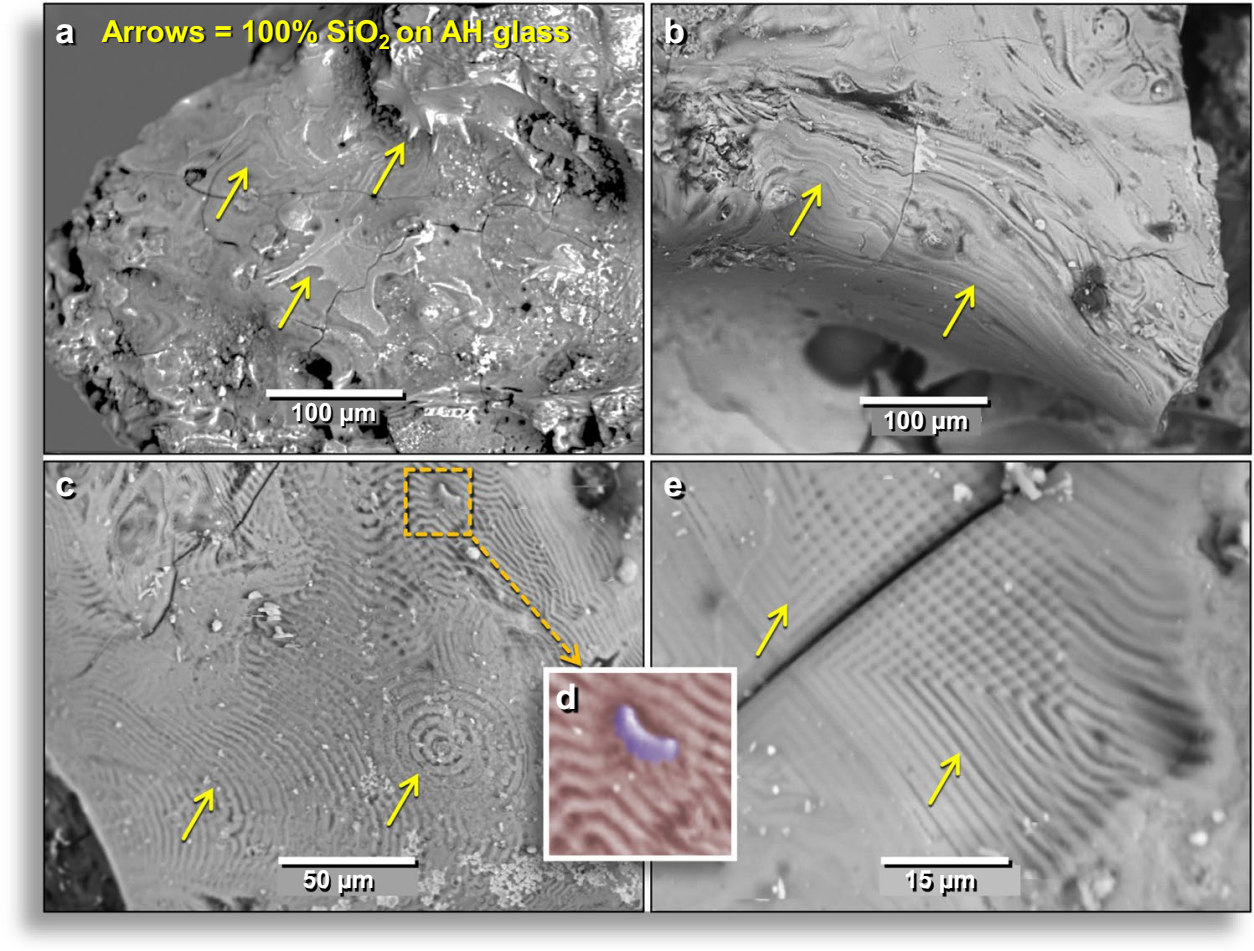
SEM images of pure SiO_2_ (lechatelierite). (**a**) Lechatelierite coating with swirling flow marks on an outer surface of AH glass. Sharp lechatelierite spikes are shown at the upper yellow arrow. From level 445, sample E301, 405 cm depth. (**b**) Lechatelierite coating on an outer surface of AH glass with oriented flow marks at arrows. (**c**) Distinctive patterns produced during the cooling of lechatelierite. The lechatelierite is a transparent outer layer on the wall of an interior vesicle and covers the Ca-rich aluminosilicate glass matrix. (**d**) Manually constructed EDS-based phase map shows how the surface irregularity (purple) distorted the thin lechatelierite coating (light red) that flowed over and around it, distorting the textural ridges. (**e**) Distinctive lechatelierite pattern that appears orthogonally oriented relative to crack in vesicle wall of AH glass. The lechatelierite coating is only several microns thick and these distortions indicate that lechatelierite flowed around the pre-existing grain. Glass samples are from level 457, sample E313, 413 cm depth.

SEM-EDS analyses indicate that surfaces and vesicles of AH glass are commonly coated with lechatelierite (Figs. 5, 6**)**, with a typical composition of SiO_2_ at 88.3–98.7 wt.%. Other oxides showed an average of 2.9 wt.% for MgO; 2.0 wt.% Al_2_O_3_; 1.3 wt.% K_2_O; 1.2 wt.% CaO; and 3.8 wt.% FeO. Many fragments of AH glass display sinuous flow marks^8^, indicating that high temperatures lowered viscosity enough so that the molten glass flowed before cooling very rapidly (Fig. 6a,b). In other cases, AH glass surfaces and vesicles display distinctive, highly organized, *moiré*-like textural patterns that appear to have formed as the molten glass cooled (Fig. 6c–e). In some examples, surface textures are distorted by irregularities in the glass surface (Fig. 6c,d), indicating that the thin lechatelierite surface coating flowed around topographical irregularities. In other cases, the cooling patterns appear to have conformed to cracks in AH glass that existed prior to deposition and cooling (Fig. 6e). These thin lechatelierite coatings most likely resulted from complete, high-temperature vaporization/deposition of quartz grains and/or of silica-rich plant parts. If so, the *moiré*-like patterns (Fig. 6c–e) likely formed during a rapid process analogous to vapor deposition (Appendix, Text S3).

#### Melted quartz grains (SiO_2_)

**Figure 7:**
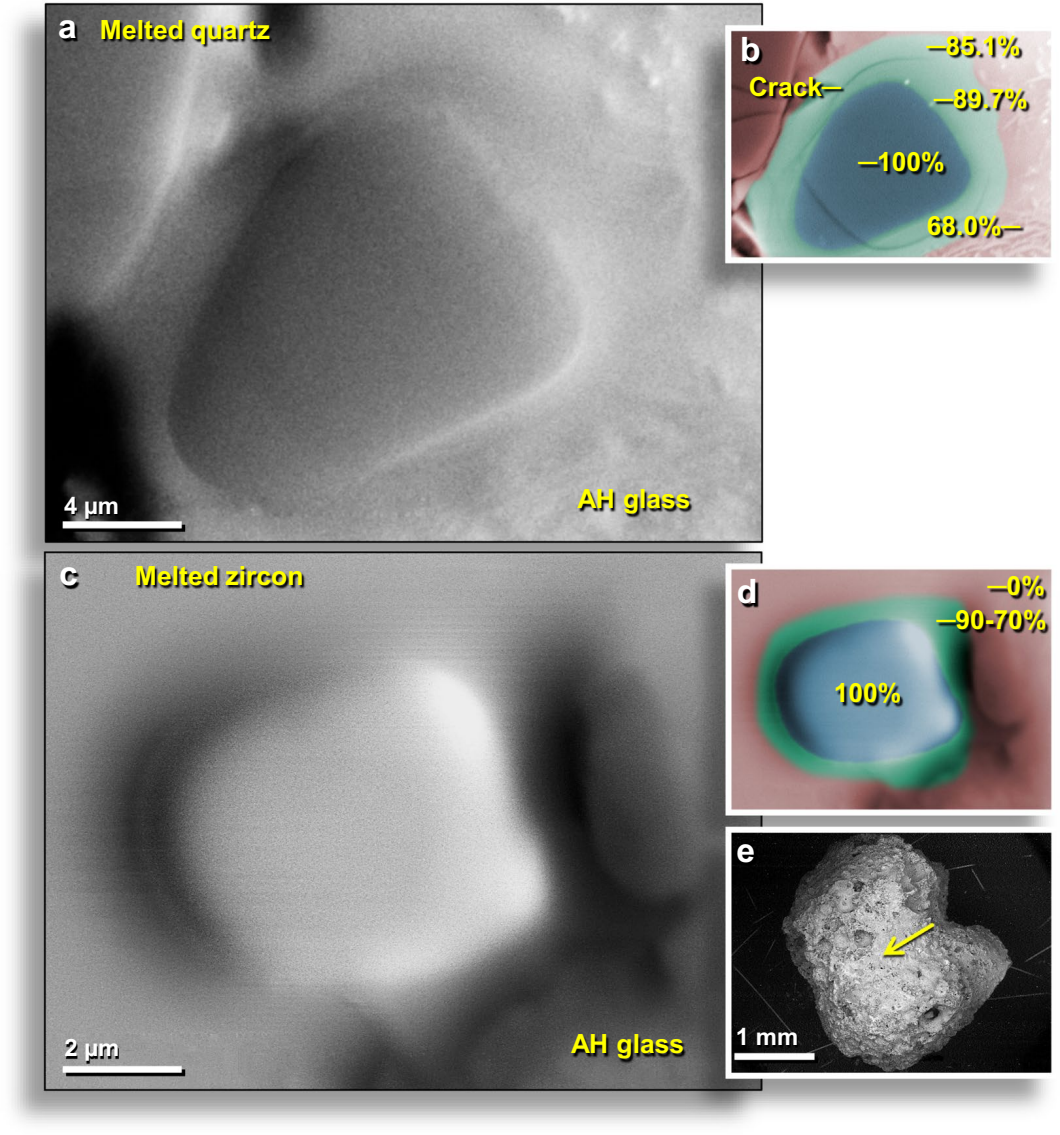
SEM images of melted AH quartz grain. (**a**) SEM topographical image of 15-μm-wide grain on the inner wall of an AH glass vesicle. The darker gray central portion of the image is a smooth, melted quartz grain with no apparent crystalline structure. From level 445, sample E301, 405 cm depth. (**b**) Manually constructed EDS-based phase map with light red representing the aluminosilicate glass matrix, blue representing melted quartz grain, and green representing zone of diffusion of SiO_2_ into the matrix. The crack was likely caused by thermal contraction during cooling. Percentages of SiO_2_ range from 100 to 68.0 wt.%). (**c**) SEM topographical image of melted zircon of melted 8-μm-wide zircon grain embedded in an outer surface of AH glass. (**d**) Manually constructed EDS-based phase map of same grain with the zircon weight percentage ranging from 100 → 90 → 70 → 0 wt.%. **(e)** SEM image showing the location of zircon grain in melted 2.7-mm-wide AH glass fragment. From level 449, sample E305, 412 cm depth.

To determine maximum potential temperatures, we examined the surfaces of the AH meltglass for melted quartz (SiO_2_) that typically melts at ~1720 °C. Light microscopy, SEM-EDS, and microprobe were used on both sectioned and non-sectioned materials to identify partially melted and fully melted quartz grains. One 15-μm-size quartz grain (Fig. 7a) was fully melted but generally retains its original outline; SEM-EDS analyses show that the central melted zone is 100 wt.% SiO_2_ (Fig. 7b). Immediately adjacent to the outline of the grain, the diffusion zone (Fig. 7b) is composed of 10.3 wt.% typical sedimentary oxides (MgO, Al_2_O_3_, K_2_O, FeO, and CaO with no other oxides detected). Si and O account for the remaining 89.7 wt.%. If stoichiometric, the predicted amount of O is 58.0 wt.%, but instead, there is only 31.7 wt.% of O. This oxygen concentration is insufficient to produce oxides of the total measured concentrations of Mg, Al, K, Ca, and Si. These measured percentages suggest that the mixture possibly is composed of ~59.4 wt.% SiO_2_ and ~30.3 wt.% native (elemental) Si. Native Si occasionally is produced under highly reducing conditions in volcanic exhalations, in some mantle-derived rocks, and in meteorites but is extraordinarily rare in other terrestrial settings.

One observed melted example is an unusual 125-μm-wide shattered grain, an example of monomict quartz breccia (Appendix, Fig. S7). This is similar to brecciated rocks found at known impact sites, such as Meteor Crater, Arizona, where the surface impact of a NiFe impactor generated an extreme mechanical and thermal shock that fractured rocks and melted/boiled material along fracture zones. SEM-EDS analyses indicate that SiO_2_ percentages range from 100 wt.% to 63 wt.% (Appendix, Fig. S7c**)**.

Another piece of high-temperature AH glass exhibits an area ~260 µm wide in which one or more completely melted quartz grains are diffusing into the aluminosilicate matrix (Appendix, Fig. S8). SiO_2_ percentages are in a gradient from 100 to 64.7 wt.%. A large highly vesicular, 1040-μm-wide quartz grain shows evidence that it reached the boiling temperature for quartz of ~2230 °C (Appendix, Fig. S9). The central part of the grain is unmelted quartz displaying a highly fractured and vesiculated surface (Appendix, Fig. S9b), surrounded by an area composed of an oxygen-deficient mix of native Si and SiO_2_ (Appendix, Fig. S9b). The high percentage of Si from the original grain and the highly vesicular nature strongly suggest that the edges of the grain were boiling at ~2230 °C. The SiO_2_ content continued to decline as the melted grain diffused into the AH glass matrix (Appendix, Fig. S9). A ternary diagram supports a theoretical minimum temperature range of ~1250 °C for the AH glass matrix and ~1720 °C for the quartz grain (Appendix, Fig. S10). Furnace experiments suggest that some fully melted quartz grains embedded in AH meltglass were subjected to temperatures of >1700 °C (Appendix, Fig. S11**)**, whereas other grains show significant outgassing suggesting that they may have reached ≥2230 °C. In addition, the diffusion zones around some quartz grains in AH glass appear to contain elemental or native Si, suggesting that melting occurred in an oxygen-deficient atmosphere.

Because the accuracy of these analyses is crucial to identifying native Si, SEM-EDS analyses were performed on three reference quartz grains. In pure quartz, the expected value for Si is 46.7 wt.%; our average measured value for the three quartz grains using SEM-EDS was 46.1 wt.% (range 45.2 to 46.6 wt.%). The expected value for O is 53.3 wt.% and the average measured value was 53.9 wt.% (range 53.4 to 54.8 wt.%). These analyses indicate that the identification of oxygen-depleted native Si is accurate.

#### Ca silicate (wollastonite)

Crystals of another Si-rich mineral, calcium silicate (CaSiO_3_, wollastonite) were commonly observed on the outer surfaces and vesicle walls of AH glass but were never found inside the glass or on top of plant imprints (Appendix, Fig. S12). Wollastonite follows the triclinic crystal system, often forming distinctive star-like crystals. This mineral typically melts at 1540 °C with an estimated flux-mediated melting point of ~1340 °C.

### Zirconium-rich minerals

#### Melted zircon, AH meltglass

A surface of one piece of AH glass contained a melted 8-μm-wide zircon grain (Fig. 7c–e). SEM-EDS analyses revealed a composition of Zr at 46.1 wt.%; Si at 15.8 wt.%; O at 36.0 wt.%; and Hf at ~2.1 wt.%. Zircons typically melt at ~1775 °C with a flux-mediated melting point of ~1575 °C. Full melting caused the zircon to lose its characteristic euhedral shape and to diffuse into the aluminosilicate AH glass matrix with weight percentages decreasing from 100 → 90 → 70 → 0 wt.% in the matrix.

### Chromium-rich minerals

**Figure 8:**
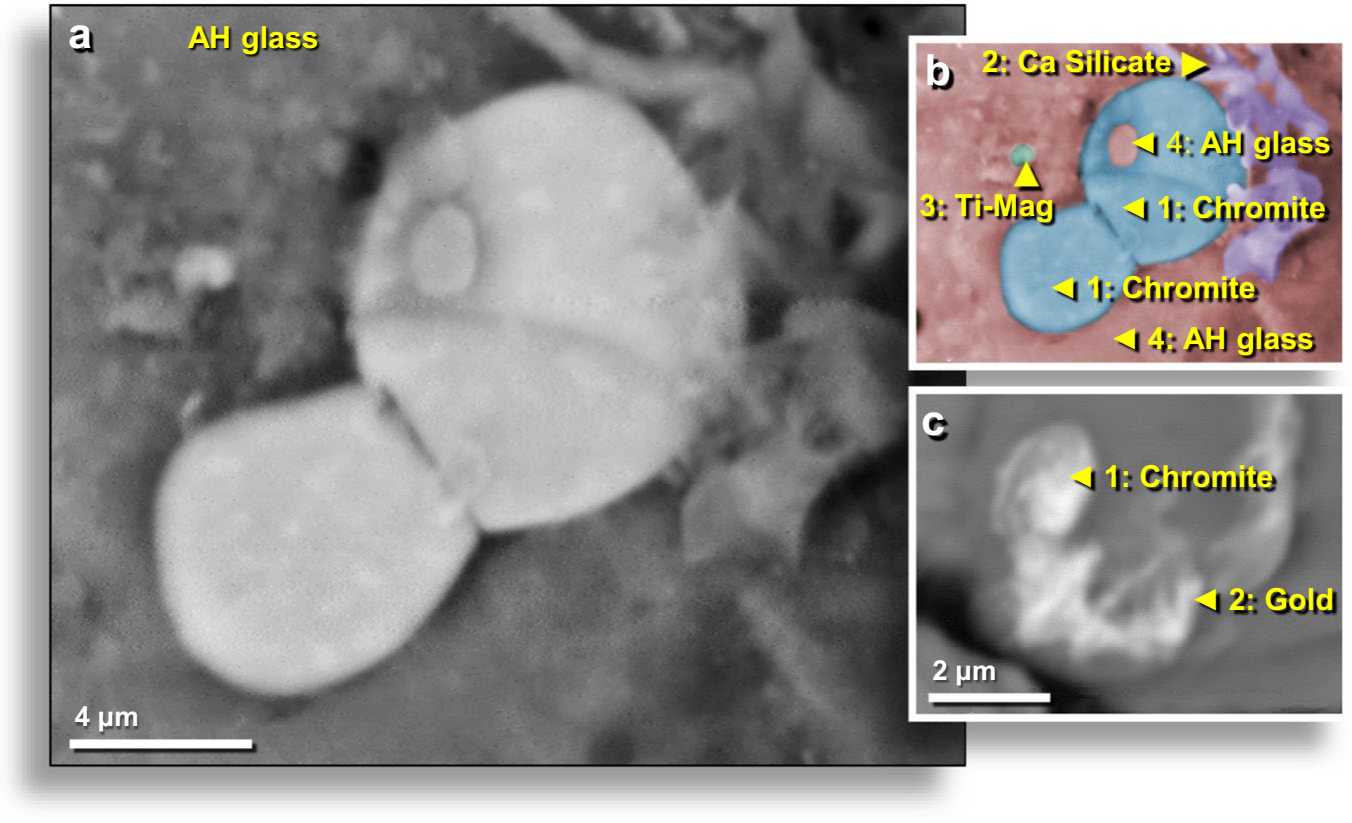
Melted chromite grains. (**a**) Two possible chrome-magnetite grains (Fe^2+^(Fe^3+^,Cr)_2_O_4_), one ~10 µm long and the other ~7.5 µm long, that melted and flowed together on the inner wall of AH glass vesicle. (**b**) Manually constructed EDS-based phase map of SEM image in (**a**) with chrome-magnetite in blue and AH glass matrix in light red. Field of view also contains melted titano-magnetite grain (green; equilibrium melting point: 1625 °C) and post-depositional, unmelted calcium silicate in purple. Note melted droplet of AH glass fused to the top of melted chrome-magnetite at upper right, indicating the melted chrome-magnetite was deposited while the AH glass was molten. (**c**) Irregular chrome-magnetite grain inside AH glass vesicle shows evidence that it melted and flowed as the glass cooled. Chromium is mixed with gold. All grains from level 449, sample E305, 412 cm depth.

**Figure 9:**
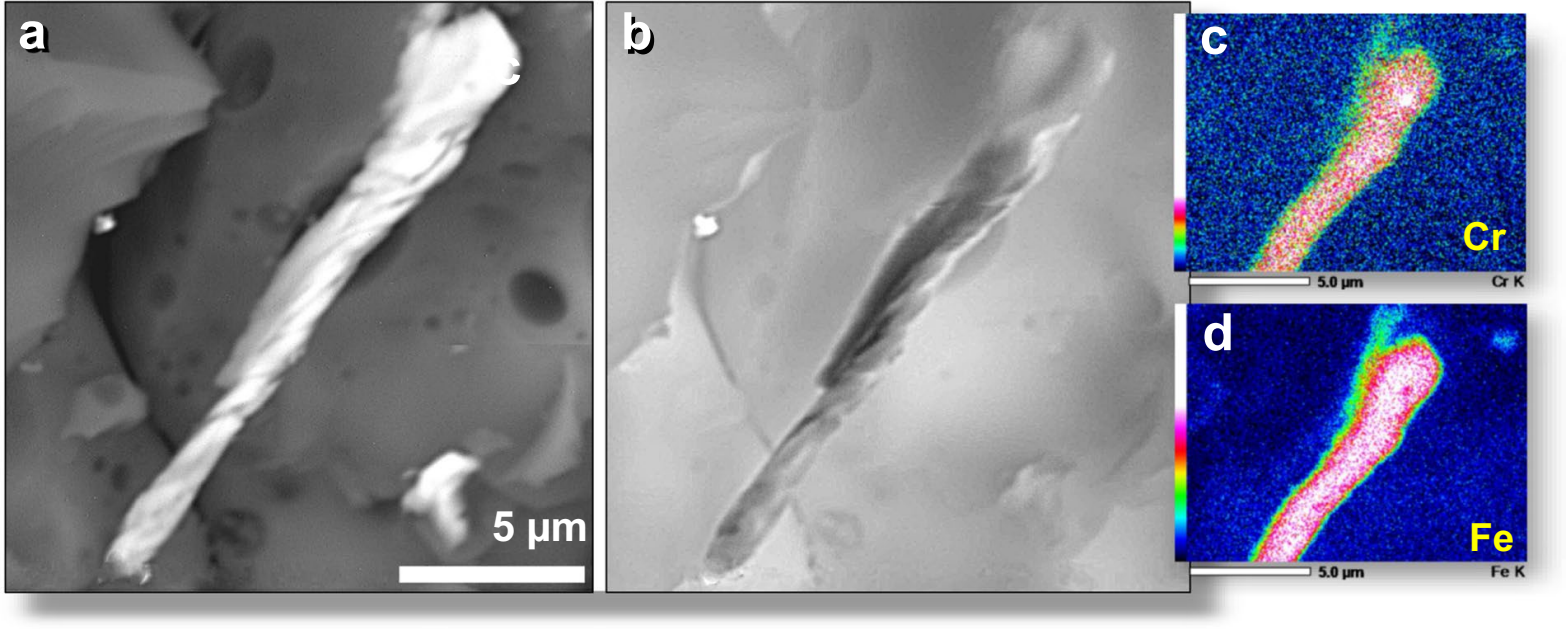
Flow marks in chromferride embedded in AH meltglass. Elongated chromferride grain, 18 μm long by 2.5 μm wide, embedded in the surface of AH meltglass. SEM-EDS indicates it is composed of 88.2 wt.% Fe and 11.8% Cr with almost no detectable oxygen. Grain displays flow marks that likely resulted from mechanical deformation while airborne and molten. Note adjacent darker bubbles in the Al-Si glass matrix. (**a**) SEM backscatter image. (**b**) SEM topographical image shows raised relief. (**c**) Single-element (Cr) EDS map with an intensity scale. (**d**) Single-element (Fe) EDS map with an intensity scale. From level 449, sample E305, 412 cm depth.

Excavated AH glass was examined for the presence of Cr-rich minerals, and the SEM-EDS analyses identified three such minerals: isovite ((Cr,Fe)_23_C_6_) with the highest percentage of Cr = 67.4 wt.%; Fe = 26.8 wt.%; C = 5.8 wt.%; chromite (Fe^2+^Cr_2_O_4_) with Cr = 46.5 wt.%; Fe = 24.9 wt.%; O = 28.6 wt.%; chrome-magnetite (Fe^2+^(Fe^3+^,Cr)_2_O_4_) with Cr = 11.3 wt.%; Fe = 60.8 wt.%, and O = 27.9 wt.% (Fig. 8); and chromferide (Fe_3_Cr_1-x_ (x = 0.6)) with the lowest percentage of Cr at 11.0 wt.% and Fe = 89.0 wt.% (Fig. 9). Chromferide, a very rare mineral, has been previously found in impact melt rocks of the El’gygytgyn Crater, Chukotka, Russia^36^. Some Cr-rich grains observed in AH glass appear to be intergrowths of chromferide and isovite. The surface of one AH glass sample contains an embedded chromium-magnetite grain that displays flow marks consistent with aerodynamic mechanical deformation (elongation and twisting) while molten (Fig. 9). The SEM topographical image shows surface relief.

### Iron-rich minerals

**Figure 10:**
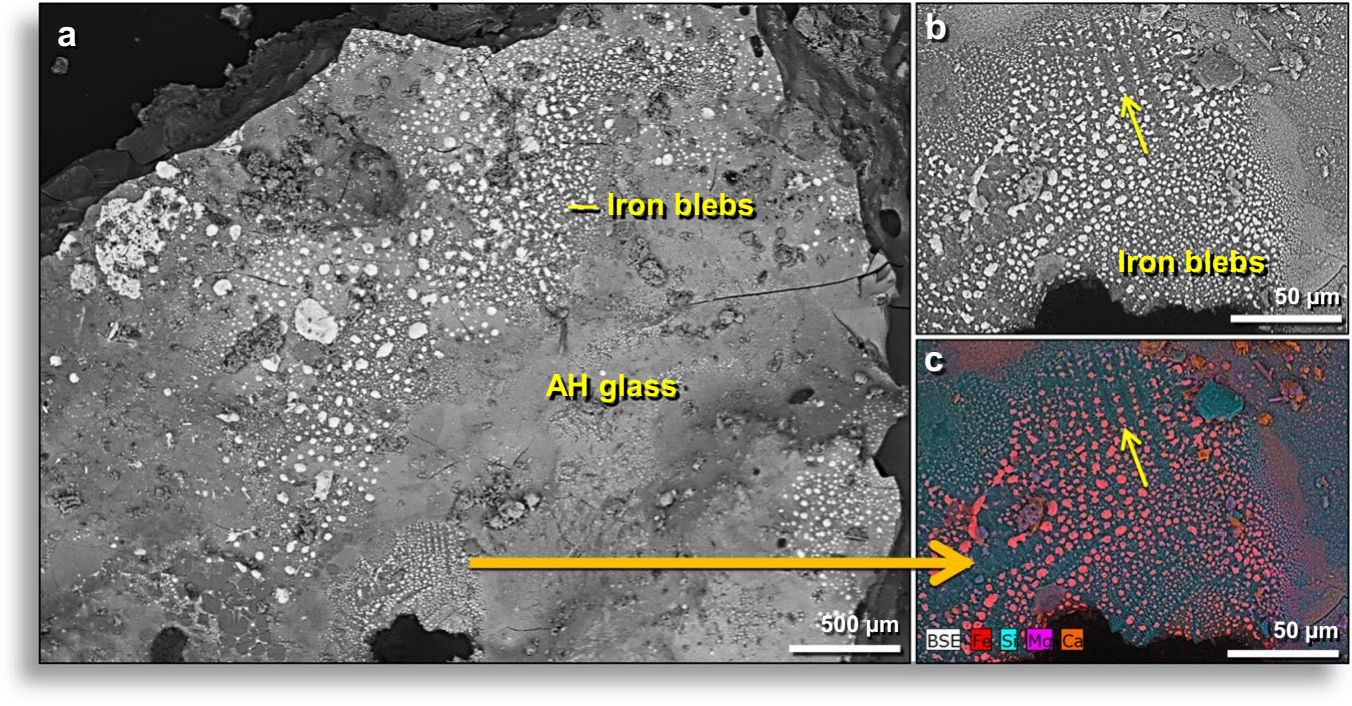
Fe-rich globules on surfaces and in vesicles of AH glass. (**a**) SEM image of hundreds of Fe-rich globules on the outer surface of AH glass. Some globules are FeO and others are iron silicides. Note that globules occur only on original surfaces and not on broken ones (upper left and upper right). Gold arrow points to closeup of same region. (**b**) SEM close-up of globules. Some are round, but most are irregularly shaped. All are flattened, convex globules embedded on the surface of AH glass. (**c**) Multi-element SEM-EDS map of same area of (**b**) showing the distribution of multiple elements (see the color legend at bottom left). Fe-rich globules (red) on the AH glass matrix containing Ca and Si, with smaller amounts of Mg and/or Ni. All from level 457, sample E313, 413 cm depth.

**Figure 11:**
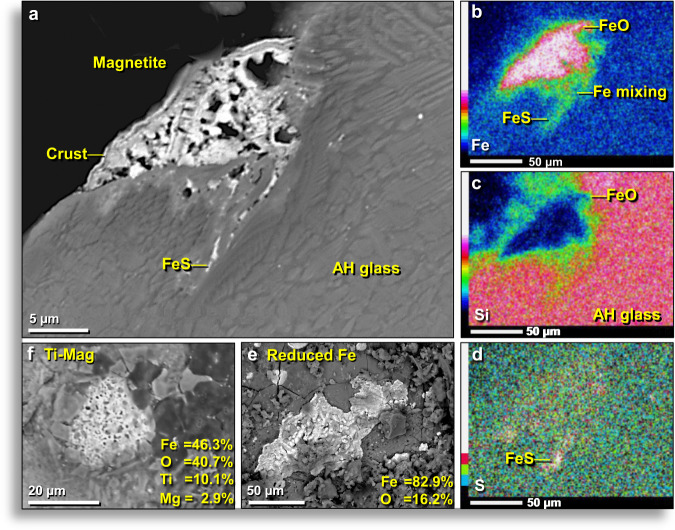
Different forms of iron in Abu Hureyra meltglass. (**a**) SEM image of melted 23-μm-wide magnetite grain embedded in the outer surface of AH glass. Note crust on grain formed from thermal and chemical alteration. The high degree of vesiculation suggests magnetite possibly boiled at ~2623 °C. (**b**) Single-element (Fe) EDS map with intensity scale showing the diffusion of Fe into the glass matrix. This map also reveals that Fe combined with S to produce iron sulfide, FeS (discussed below). (**c**) Single-element (Si) EDS map with intensity scale showing Si-rich composition of the AH glass matrix. (**d**) Single-element (S) EDS map with intensity scale indicating the location of troilite (FeS). (**e**) Irregularly shaped, 150-μm-wide Fe globule on the outside surface of AH glass, composed of highly reduced iron at 95.1 wt.% Fe and the remainder as oxygen. (**f**) Melted 27-μm-wide titano-magnetite grain on the outside surface of AH glass. The equilibrium melting point is 1625 °C. High vesiculation suggests titano-magnetite grain reached or exceeded its boiling point. All samples from level 445, sample E301, 405 cm depth.

SEM-EDS analyses of the outer surfaces of AH glass and interior surfaces of vesicles revealed a high abundance of melted Fe-rich globules, mostly FeO, but sometimes containing native Fe and Fe silicides. Iron globules were only observed on outer surfaces of AH glass and inside vesicles but never on broken interior surfaces. Sometimes, the globules appear to have solidified atop the molten glass (Fig. 10); at other times, the globules are organized in rows (Fig. 10b,c), suggesting that they formed inside vesicles by vapor deposition of Fe. The surfaces of Fe-rich AH glass often display distinctive patterns that appear dendritic (branch-like or feather-like) (Appendix, Fig. S13). During the melting of AH sediment, it appears that these dendritic Fe crystals grew mostly by vapor deposition, as the molten glass cooled rapidly. Other forms of Fe also were observed (Fig. 11a–d), including melted magnetite (Fe^2+^Fe^3+^_2_O_4_) (Fig. 11a) and melted titanium-magnetite (Fe^2+^(Fe^3+^,Ti)_2_O_4_) (Fig. 11f), containing ~10.1 wt.% Ti. We also identified highly reduced iron (FeO), containing 95.1 wt.% Fe and only 4.9 wt.% O (Fig. 11e),

Oxygen fugacity (abbreviated ƒO_2_) relates to the vapor pressure of oxygen. Under low ƒO_2_, little oxygen is available to combine with other elements to produce oxides and so low-oxygen minerals form^37^ in an unusual occurrence (Appendix, Text S4). Formational environments of native Fe and magnetite differ by two log units of ƒO_2_ and by one to two log units between native Fe (Fe°) and Fe silicides. The oxygen fugacity of Fe-rich objects varied from highly reduced (forming native Fe and Fe silicides) to typical oxidizing conditions (forming magnetite), a range of several log units^37^. Based on the Fe-bearing mineral phases attached to vesicle walls of AH glass, it is inferred that exposure temperatures range from the equilibrium melting point of magnetite at ~1590 °C to its boiling point at ~2623 °C.

#### Melted nickel-rich iron

**Figure 12:**
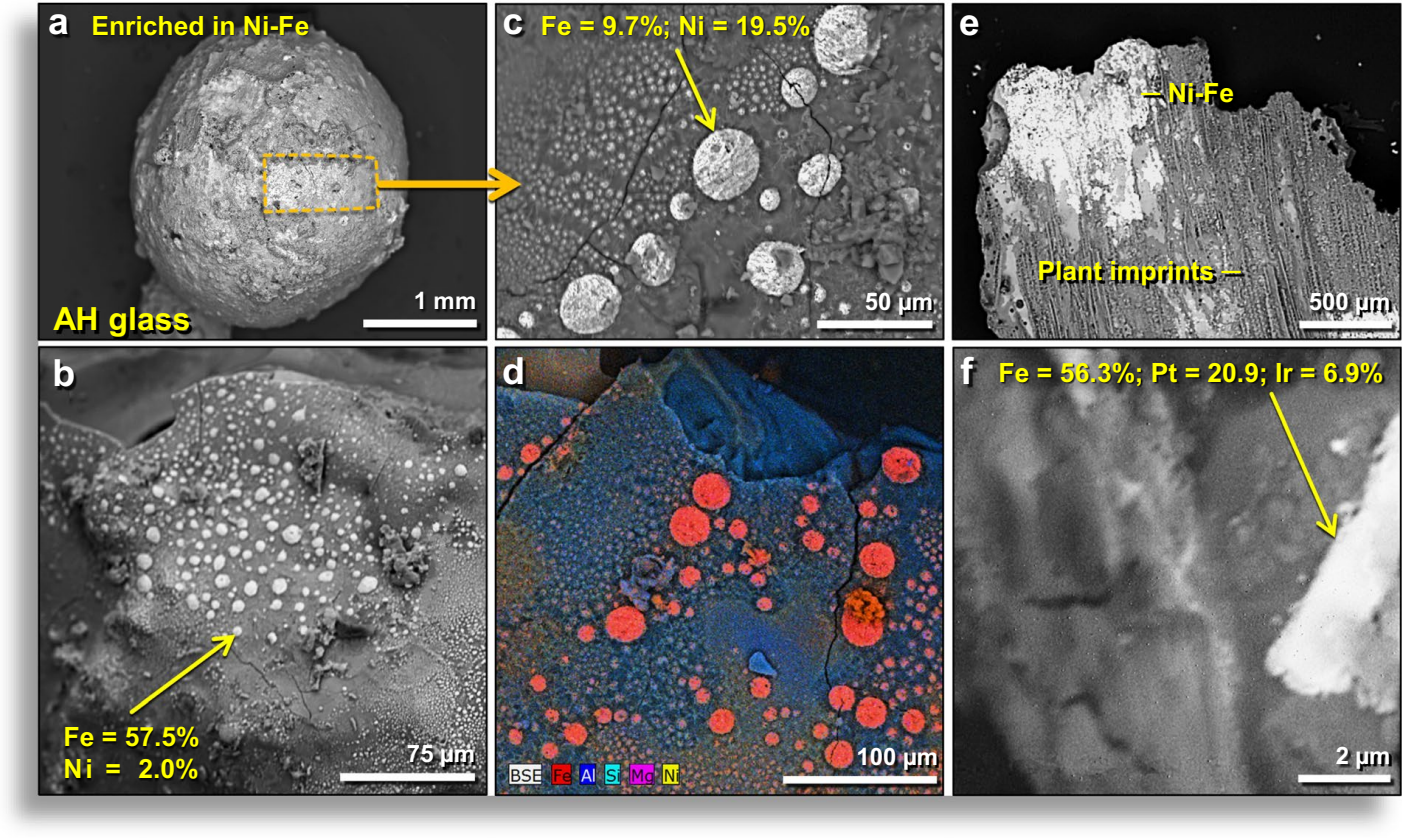
SEM images of Fe enriched in Pt, Ir, and Ni on outer surfaces of AH glass. (**a**) Melted 2.5-μm-wide AH subrounded glass. Box marks area with abundant NiFe globules. (**b**) Surface of AH glass with hundreds of NiFe globules with Ni content of ~2.0 wt.%. (**c**) Inside of vesicle in AH glass shown in (**a**), containing hundreds of NiFe globules. Middle globule at arrow contains ~19.5 wt.% Ni and ~9.7 wt.% Fe. (**d**) Multi-element EDS map (see the legend at lower left) of the surface of AH glass displaying numerous NiFe globules (reddish-orange), a combination of Fe (red) and Ni (yellow). Note there are no globules on the broken upper edge, indicating they did not form inside the AH glass, only on the surface, suggesting possible formation by vapor deposition. (**e**) Thin, flat fragment of AH glass with plant imprints and NiFe globules. Note that plant imprints formed on top of the NiFe, indicating that the imprinting occurred after the glass melted. (**f**) AH glass with long grain with ~20.9 wt.% platinum and ~6.9 wt.% iridium. (**a**–**d**) from level 457, sample E313, 413 cm depth. (**e**) from level 445, sample E301, 405 cm depth. (**f**) from level 449, sample E305, 412 cm depth.

Our SEM-EDS investigations of AH glass identified three types of Ni-enriched minerals: (a) flattened, elliptical globules of Ni-magnetite ((Ni,Fe)_2_O_4_) are common on vesicle walls inside AH glass (Fig. 12); (b) awaruite (Ni_3_Fe) is commonly found on the surfaces of AH glass spherules; and (c) small NiFe spheres and hemispheres (<200 µm in diameter) are found in linear alignments, in random groupings, and as isolated spheres often bordered by Ni-rich glass created by Ni diffusion (Fig. 12). There are also enrichments in concentrations of nickel (300 ppm), cobalt (68 ppm), and chromium (3750 ppm) in the YDB magnetic fraction of sample E301 at a depth of 405 cm, compared to typical concentrations in background samples of ≤20 ppm for Ni, ≤50 ppm for Co, and <800 ppm for Cr (Appendix, Table S7).

The Ni-rich grains are dominated by three NiFe-rich minerals: kamacite (NiFe with Ni concentrations at 2–6.8 wt.%; Fe = 70–73.8 wt.% and the remainder as oxygen); taenite (NiFe with a higher percentage of Ni at 7.3–28.6 wt.%); and awaruite (Ni_2_Fe to Ni_3_Fe). Ni-magnetite is usually limited to hydrothermally serpentinized mafic zones of subducted ophiolites^38^ but is also found in some meteorites. These Ni-rich materials are most likely terrestrial in origin but with possible mixing with a small amount of meteoritic material, because most impactites contain very small meteoritic contributions (usually well below 1 wt.%)^39^.

The Ni-bearing spherules formed under highly reducing conditions at low ƒO_2_ of approximately −6 to −8 log units, sometimes only a few microns away from oxidized magnetite that formed at a very different fugacity. This high variation over very short distances indicates highly variable and chaotic formation conditions. Kamacite and taenite usually only form in highly reduced environments with ƒO_2_ of approximately −10 log units (iron-wüstite or IW buffer). Native Fe and NiFe are common inside several AH glass vesicles, suggesting temperatures of >1500 °C. Thus, some AH meltglass formed. We propose that the NiFe spherules resulted from the melting at >1500 °C under oxygen-deficient conditions (ƒO_2_ between log −6 and −8) of AH bulk sediment with ~0.3 wt.% NiO, also containing ~10% Ni-rich magnetite, potentially mixed with Ni from the impactor.

#### Melted iron with platinum and iridium

SEM-EDS analyses identified a melted PGE-rich grain embedded in a vesicle that contains ~20.9 wt.% Pt; ~6.9 wt.% Ir; and ~56.3 wt.% Fe (Fig. 12f). This nugget was embedded in the wall of the vesicle when the AH glass was molten. The occurrence of Pt and Ir in AH glass is consistent with neutron activation analyses which showed high Pt concentrations in the YDB layer, in which peak concentrations of 6.2 ppb Pt were measured in bulk sediment and of 8.1 ppb Pt in the magnetic fraction from sample E301, the YDB layer (Appendix, Table S7). Layers above and below showed negligible concentrations of Pt.

#### Melted iron silicide

**Figure 13:**
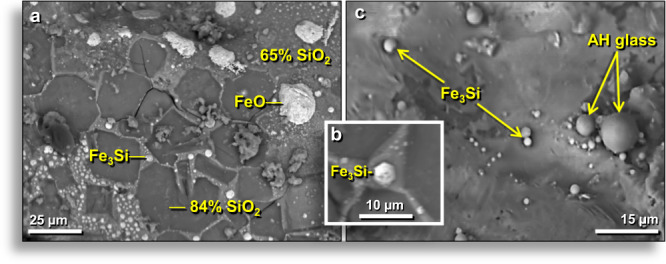
SEM images of iron silicides on the inner walls of AH glass vesicles. (**a**) Small, rounded, bright blebs or globules are iron silicide (Fe_3_Si); the largest is ~22 µm across. AH glass matrix is 65 wt.% SiO_2._ Dark, polygonal, recessed areas are 84 wt.% SiO_2_. (**b**) Close-up of hexagonal ~4-μm-wide globule of Fe_3_Si shown in (**a**). (**c**) Small higher-Z spherules of Fe_3_Si; largest is ~3 µm wide. Fragments at right are AH glass; the largest is ~9 µm wide. From level 457, sample E313, 413 cm depth.

SEM-EDS analyses identified globules of iron silicides (FeSi, Fe_2_Si, Fe_3_Si) and native Si embedded in the walls of AH glass vesicles, as well as on outer glass surfaces (Fig. 13). In AH glass vesicles, ≥1-μm-wide spherules of Fe_3_Si (suessite) (Fig. 13) have an average composition of Fe = 24.5 atom% and Si = 75.5 atom% (n = 5; Si/(Si + Fe) = 24.5), consistent with Fe_3_Si. Other spherules in the same vesicle (Fig. 13c) have compositions similar to typical Ca-Al-Si AH glass. Inside another vesicle, an array of FeSi spherules was embedded in SiO_2_-rich silica glass (SiO_2_ = 93 wt.%) with the same bulk composition as plant-imprinted AH glass, in which Fe = 48.9 atom%, Si = 51.3 atom% (Si/(Si + Fe) = 50.9 ratio), consistent with FeSi. In several AH glass vesicles, spheres of Fe_2_Si (hapkeite) were observed with an estimated elemental composition of Fe = 79 atom% and Si = 21 atom% (not the same as elemental ratios) with the structural formula of Fe_2_Si. Fe_2_Si Formula = (Fe_1.9_ + Ni_0.8_ + Cr_0.2_)2 (Si_0.9_ + P_0.1_), where Cr and Ni often substitute for Fe, and P sometimes substitute for Si.

Suessite (Fe_3_Si) is a very rare mineral on Earth, forming at high temperatures and low ƒO_2_, as for example, in fulgurites^40^. Suessite, in contrast, is common in meteorites, where the mineral was first discovered. It is commonly found in lunar meteorites; micrometeorite impact pits in Apollo samples; and highly reduced achondrites. Fe silicides in AH glass indicate exposure to extreme reducing atmospheres, 2 to 4 log units lower than the iron-wüstite (IW) buffer with temperatures >2000 °C.

It is likely that these highly reduced minerals originally formed in AH glass that contained vesicles filled with trapped gases. If so, abundant carbon, possibly from incinerated vegetation, created a reducing atmosphere that produced the reduced phases of various minerals, including Fe silicides, native Fe, and native Si.

### Sulfide minerals

#### Melted Fe sulfide (troilite)

**Figure 14:**
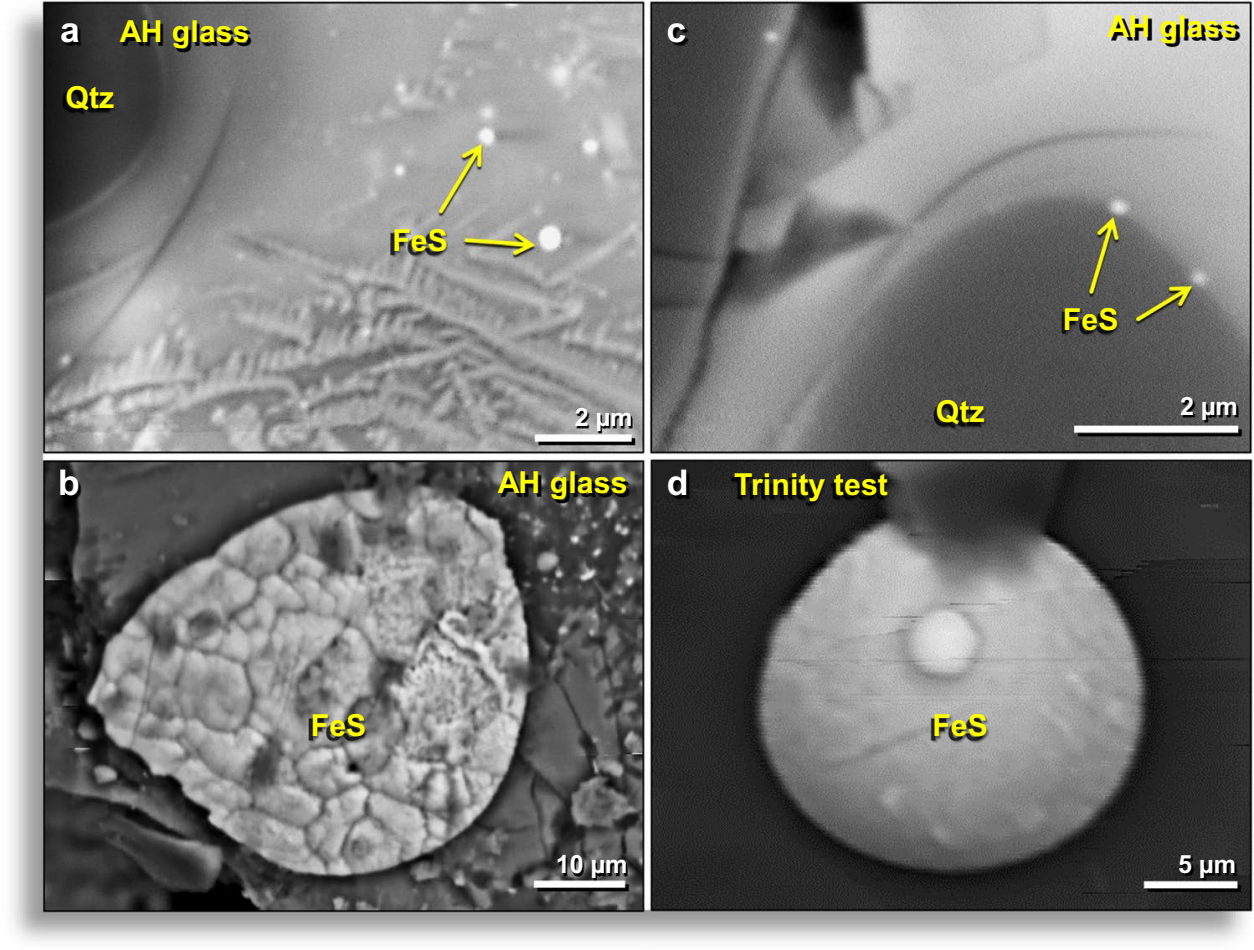
SEM images of iron sulfide (FeS) in AH glass and trinitite. All images from the inner walls of melted glass vesicles. (**a**) Sub-micron FeS spherules in AH glass matrix; the largest is ~0.4 µm wide. (**b**) Large, flattened, textured ~45-μm-wide globule of FeS on AH glass. (**c**) Tiny, 0.15-μm-wide FeS spherules in AH glass along the edge of melted quartz grain. (**d**) Convex disc of ~20-μm-wide FeS globule on the surface of trinitite glass. Small rounded FeS globule lies on the larger disk. AH glass from level 457, sample E313, 413 cm depth. Trinitite sample provided by co-author R.E.H.

In some fragments of AH glass, globules of the mineral troilite (iron sulfide or FeS) were observed with a composition of ~64 wt.% Fe and ~36 wt.% S. FeS globules were observed only on surfaces of AH glass fragments and inside of vesicles (Fig. 14) but not within the meltglass, suggesting that they formed inside vesicles by vapor deposition.

#### Melted Titanium sulfide

The inspection of surfaces and vesicles of AH glass revealed melted titanium sulfide (TiS) that is commonly associated with melted magnetite and other high-temperature melted minerals (Appendix, Fig. S14). When oxygen is sufficient the titanium in Ti-rich minerals typically combines with oxygen upon melting, but when there is insufficient oxygen, Ti combines with S to form sulfides and P to form phosphides. Thus, the presence of TiP and TiS in AH glass suggests high-temperature melting under highly reducing conditions.

### Rare-earth-rich phosphate (monazite)

**Figure 15:**
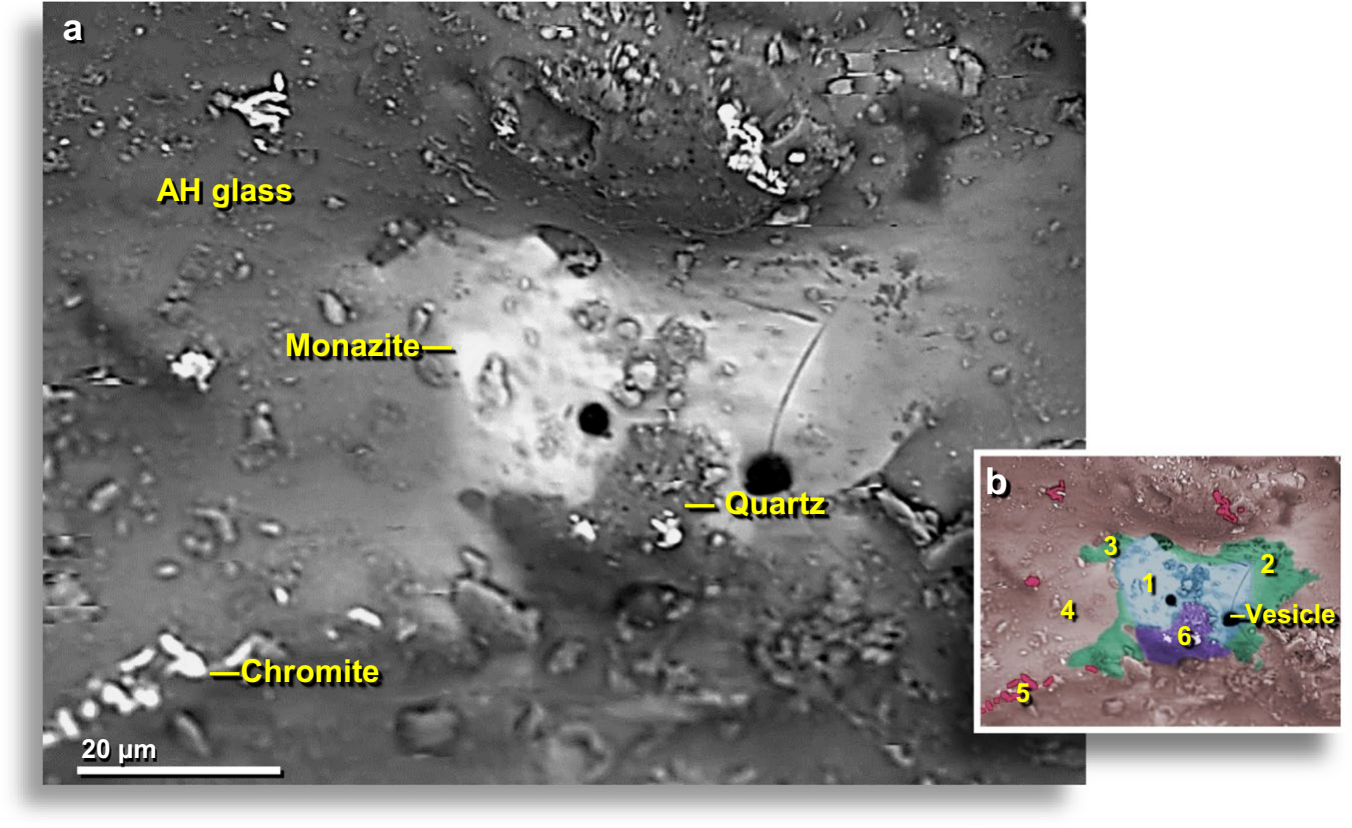
SEM images of melted monazite in AH glass. (**a**) Bright material at center is a partially melted ~35-wide monazite grain on the outer surface of AH glass. This uncommon rare-earth phosphate grain has been partially absorbed by AH glass with decreasing EDS-measured percentages of monazite (100 → 54 → 46 → 0 wt.%). The equilibrium melting point of monazite is ~2072 °C. In addition, more than a dozen small, partially-melted, chromite grains are scattered throughout the image, mostly at the lower left. (**b**) Manually constructed EDS-based phase map showing the remnants of vesicular monazite grain (blue, #1) with diffusion of monazite components (green, #2-#3) into AH glass (light red, #4); globules of chromite (dark red, #5). Melted ~14-μm-wide quartz grain (purple, #6) is highly vesicular, suggesting it reached boiling temperatures of ~2230 °C. From level 449, sample E305, 412 cm depth.

Inside some AH glass vesicles, SEM-EDS analyses identified melted monazite, a rare-earth phosphate ((Ce,La,Nd,Th)PO_4_). In one sample, a faint outline of the original grain is still visible, but the grain has no distinct boundary due to melting and diffusion into the AH glass matrix (Fig. 15). The monazite grain surrounds part of a melted quartz grain that is in partial contact with the AH glass. The large gas bubbles visible in the monazite grain suggest outgassing as the monazite broke down and released oxygen at temperatures of ~2230 °C^41^. This observation is consistent with rapid cooling from a superheated liquid. As the material cooled, it reached a blocking temperature at which the vapor phase could no longer equilibrate with the liquid phase. At that stage, oxygen from the breakdown of oxides remained in the vapor phase, and the liquid phase became oxygen-deficient. This can only result from heating followed by very rapid cooling. The enclosing quartz grain also contains numerous small vesicles, suggesting that it possibly reached its boiling point of ~2230 °C, the same temperature required to boil the monazite grain.

### Remanent magnetism as a formation criterion

We considered whether Fe-rich spherules and meltglass from Abu Hureyra may have been produced by lightning strikes. To investigate, we measured remanent magnetism, defined as the magnetization remaining after molten Fe-rich material cools while exposed to Earth’s magnetic field (e.g. geomagnetism) or some other intense magnetic field. Molten magnetic minerals eventually cool to a blocking temperature (Curie point), the point at which magnetic remanence is essentially fixed. All Fe-rich terrestrial magnetic minerals, whether they have been melted or not, possess a natural remanent magnetization (NRM)^42^. If those minerals were once melted, the resulting TRM (thermoremanent magnetization) provides a record of the strength of Earth’s natural magnetic field at the time the Fe-rich material cooled. This is the most commonly observed natural magnetization. The magnetic arrangement can vary from nearly perfect (saturation state) to nonexistent (demagnetized state), depending on the level of intensity of the ambient field during the cooling of the magnetic mineral. The efficiency of the magnetic intensity level is referred to as REM^43^ and follows an empirical magnetic acquisition law^44^. Some previously molten minerals can hold this magnetic arrangement longer than the age of our solar system^45^.

If any given material has a remanent magnetic value that differs from that of Earth’s natural ambient field, these measurements provide clues to the formation of that material. During an impact event, the magnetic oxides that formed during the quenching process in melted impact materials typically display low values for remanent magnetism, but they can be higher than normal background values, possibly related to ionization and charge separation within the plasma generated during an impact (or perhaps blast)^46,47^. Alternately, lightning-produced fulgurites typically display very high values for remanent magnetism.

In these experiments, the sample’s remanent magnetization was compared to its maximum value, called its saturation remanent magnetization. Induced magnetization and susceptibility are dependent terms (Mi = XB, where Mi is induced magnetization, X susceptibility, and B external field), indicative of the “induced magnetization” that characterizes the immediate response of the sample to the current external magnetic field.

#### Remanent magnetism of AH magnetic spherules

Six representative Fe-rich spherules were extracted from AH sediment, and a superconducting magnetometer was used to measure magnetic remanence (Appendix, Tables S8 and S9). Magnetic efficiency values were obtained^48,49^, along with efficiency demagnetization spectra (Appendix, Fig. S15a) that indicate levels of both paleomagnetic field intensity and magnetic coercivity, the measure of ferromagnetic material’s ability to withstand an external magnetic field without becoming demagnetized^50,51^. None of the six Abu Hureyra Fe-rich spherules contained remanent efficiency values within the range characteristic of lightning (Appendix, Tablse S8 and S9). During the demagnetization of these spherules, the magnetic vectors preserved their direction until the maximum level of demagnetization was reached (Appendix, Fig. S15b). This result indicates that the magnetic acquisition of the spherules originated from one direction at the moment the sample cooled through its magnetic blocking temperature while exposed to Earth’s geomagnetic field. These results conclusively indicate that the tested AH spherules did not acquire their remanent magnetism from lightning discharges.

#### Remanent magnetism of YDB meltglass

**Figure 16:**
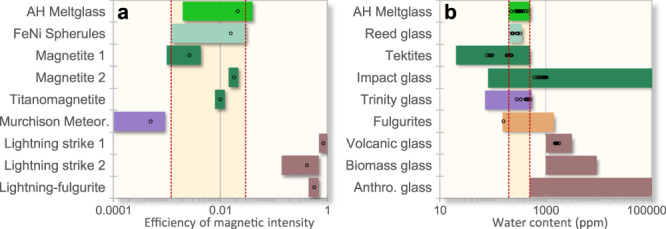
Remanent Magnetism and Water Content (**a**) Magnetization efficiency of Fe-rich materials. Abu Hureyra closely matches lab-produced anthropogenic NiFe spherules, along with terrestrial magnetite, and titanomagnetite. Meteorites are lower in remanent magnetism and lightning-produced materials are much higher. Red vertical dashed lines indicate typical terrestrial values. (**b**) Graph and table of the water content of various glasses. The uppermost medium green bar represents water content in Abu Hureyra (AH) glass. The light green bar represents laboratory-produced, high-temperature reed glass. Red vertical dashed lines indicate upper and lower limits of water content in AH glass, which overlaps that of tektites (Australasian field), impact glass, trinitite, and fulgurites. The water content of AH glass is lower than measured examples of volcanic glass, biomass glass, and anthropogenic glass, eliminating those as likely formation mechanisms.

Remanent magnetism was measured for 3 samples of AH meltglass (Appendix, Tables S8 and S9). The Abu Hureyra meltglass samples contain normal magnetization with one component, meaning that these samples most likely were not produced by lightning^43^ **(**Fig. 16a**)**. However, the results on the magnetic signatures are complicated. On the one hand, the inferred oxygen fugacities and mineralogies indicate intense temperatures and a reducing environment consistent with a plasma, which would have partially blocked (rather than enhanced) the terrestrial field. On the other hand, the results here indicate that some materials examined escaped this environment and cooled under conditions beyond the effects of any plasma. In other words, variable remanent magnetism (enhanced or reduced) in melts could indicate either lightning or a fireball, depending on their location during cooling.

### Water content as a temperature indicator

The water content of a melted material can be diagnostic for inferring origin by high or low temperatures, principally because very high temperatures typically cause outgassing of water and other volatiles, leaving behind ppm concentrations of water^52^. The H_2_O content was measured in AH glass, using established techniques of FTIR^53,54^. Measured absorbance was calculated by measuring the detected energy from light passing through the sample and comparing that with a reference spectrum using no sample.

The results are shown in Fig. 16b (Appendix, Table S10). The Abu Hureyra YDB glass was extracted from bulk sediment sample ES15, level 435, at a depth of ~392 cm. Water content ranged from 222–460 ppm (n = 19 measurements). This suggests that plant-imprinted AH glass was subjected to similarly high temperatures. The geochemical composition of samples was determined by microprobe (results in Appendix, Table S4).

## Discussion and Implications

In this study, high-temperature minerals were observed in and on AH glass, including chromite, monazite, zircon, quartz, magnetite, and iron silicides, with melting points ranging from ~1410 °C to ~2265 °C (Table 1). This Discussion focuses on four crucial questions. (i) **Laboratory experiments**. Under what temperatures and conditions can these melted minerals be produced in the laboratory. (ii) **Potential Formation by Non-impact Processes**. Can AH glass be explained by non-impact terrestrial processes, such as volcanism, anthropogenesis, lightning, and/or biomass burning? (iii) **Comparison to known Impact Meltglass**. Is AH glass closely comparable to known impact material, such as tektites? (iv) **Similarity to Proxies from other YDB Sites**. Is AH glass closely comparable to meltglass and other proxies from other YDB sites proposed to have resulted from cosmic impacts?

**Table 1:** High-temperature minerals.

Phase	Formula	~Equilib Melt T (°C)	~Est. Flux Melt T (°C)	~Equilib Boil T (°C)	Est. O_2_ Fugacity (log)	Oxygen Fugacity Buffer
Chromite	(Fe)Cr_2_O_4_	2265	2065	2642	−14	—
Monazite	(Ce, La, Nd, Th)PO_4_	2072	1872	2230	—	—
Titanium sulfide	TiS	1780	1580	—	—	—
Zircon	ZrSiO_4_	1775	1575	2550	—	—
Chert	SiO_2_	1720	1520	2230	—	—
Quartz	SiO_2_	1720	1520	2230	—	—
Titano-magnetite	Fe^2+^(Fe^3+^, Ti)_2_O_4_	1625	1425	—	−3	Magnetite-hematite
Magnetite	Fe_3_O_4_	1590	1390	2623	−3	Magnetite-hematite
Wollastonite	CaSiO_3_	1540	1340	—	—	—
Hematite	Fe_2_O_3_	1538	1338	—	—	Magnetite-hematite
Chlorapatite	Ca_5_(PO_4_)_3_Cl	1530	1330	—	—	—
Nickel iron	NiFe	1430	1230	—	−12	Iron-wustite
Native iron	Fe	1420	1220	—	−12	Iron-wustite
Native Silicon	Si	1414	1214	—	—	—
Iron silicide	FeSi	1410	1210	—	−15	—
Titanium phosphide	TiP	1400	1200	—	—	—
Iron sulfide	FeS, Fe_2_S, Fe_3_S	1200	1000	—	—	—
Iron phosphide	Fe_3_P	1100	900	—	—	—
Ilmenite	FeTiO_3_	1050	850	—	—	—

All minerals were observed on outer surfaces of AH glass or inner walls of vesicles. Laboratory heating experiments allow us to estimate flux-mediated melting points as ~200 °C below equilibrium melting points. Selected oxygen fugacities and respective buffers are shown.

### Laboratory experiments

To duplicate the temperatures and conditions required for melting AH glass, we conducted laboratory heating experiments on zircon, chromite, titanomagnetite, and charcoal. Laboratory conditions and temperatures are described above in the section “Furnace and torch heating experiments” and in Methods.

The equilibrium melting point of zircon is ~1775 °C, but under non-equilibrium conditions in our experiments, zircon grains typically showed incipient melting at ~1500 °C and began to convert to baddeleyite (ZrO_2_), a rare zirconium oxide mineral (ZrSiO_4_ → ZrO_2_ + SiO_2_) (Appendix, Fig. S16). By 1700 °C, most zircon grains melted completely, although a small number displayed significant surficial melting while still retaining angular shapes. A minimum high temperature of ~1500 °C is inferred for melted zircon grains found in AH glass. El Goresy^55^ first described the decomposition of zircon into baddeleyite in tektite glass and glassy melts at inferred temperatures of 1775 °C to 1900 °C.

To investigate formation temperatures for melted chromite in AH glass, we performed laboratory heating experiments using AH sediment mixed with chromite and zircon grains. By 1700 °C, the maximum temperature of this test series, nearly all zircon grains had melted, leaving most of the original chromite grains unmelted (Appendix, Figs. S17 and S18). We infer that the melted chromite in AH glass was exposed to temperatures >1700 °C.

We conducted high-temperature heating experiments designed to provide baseline analyses for melted magnetite and other Fe-rich minerals. SEM imagery of the experimentally melted glass revealed numerous circular to rounded globules of various forms of iron, including magnetite (Fe_3_O_4_), wüstite (FeO), and native iron (Fe^0^) (Appendix, Figs. S19 and S20). This demonstrates that distinctive Fe-rich globules, such as those in AH glass, can coalesce and survive temperatures of 1850 °C under low ƒO_2_ conditions.

Another laboratory heating experiment on iron silicides was conducted in a spark plasma sintering furnace at ~1850 °C, using graphite crucibles to provide a carbon-rich environment that approximates the reducing atmosphere within an impact fireball. The results revealed numerous flat globules of Fe-Si-rich minerals atop AH-like glass (Appendix, Fig. S21). Because of the very low ƒO_2_, Fe, TI, and Ca combined with elements other than oxygen to yield silicides, phosphides, and sulfides, including Fe_3_Si, native Si, and calcium sulfide (CaS). These same oxygen-deficient minerals are found in AH glass, possibly suggesting that they also formed in a carbon-rich atmosphere at Abu Hureyra. Rare inclusions of highly reduced Si were observed within some FeSi globules, commonly surrounded by elongated crystals of mostly TiP, intergrown with titanium sulfide (TiS) (Appendix, Fig. S22). Similar material is found in AH glass.

To investigate the effects of high-temperature meltglass on carbon, we measured carbon reflectance for charcoal-rich meltglass formed in the laboratory by high-temperature incineration of reeds, oak wood, and pine wood. The reflectance-inferred temperatures of all samples were more than 1000 °C lower than the measured formation temperatures of the surrounding meltglass (Appendix, Figs. S4–S6). Meltglass produced from oak wood showed the largest temperature differential between reflectance-inferred temperatures at ~1069 °C. For 11 pieces of carbon-infused AH glass, the average temperature differential was 829 °C, a striking temperature differential. For AH glass, the plant material could only have been encapsulated while the glass was molten at >1200 °C, the solidus temperature, and according to Schultz *et al*.^33^, temperatures of >1500 °C are required to entrap and preserve organics in glass. We speculate that the carbon/charcoal acted as an insulator, and therefore was less affected by the high temperatures of the surrounding meltglass. Alternately, water in plant material that imprinted the molten glass may have cooled and solidified the glass almost instantly upon contact, resulting in lower temperatures for the carbon.

In one FTIR experiment, a meltglass sample was created in the laboratory by heating common reeds (*Phragmites australis*) to >2200 °C. The carbon-infused meltglass displayed a very low H_2_O content (Fig. 16b**;** Appendix, Table S10), similar to that of AH glass. At Abu Hureyra, we speculate that water in plant material that imprinted the molten glass may have cooled and solidified the glass almost instantly upon contact, resulting in lower temperatures for the carbon.

### Potential formation by non-impact processes

We used SEM, microprobe, reflectance, and FTIR to investigate the differences between AH glass and products of biomass burning, wildfires, lightning strikes, anthropogenesis, and volcanism.

Thy *et al*. proposed that AH glass did not form by cosmic impact, but rather resulted from biomass burning, such as thatched hut fires^30^. The lead author kindly provided samples of biomass slag from Africa and investigations of the outer surfaces of biomass slag samples and interior material on sectioned slides revealed numerous low-temperature melted grains, including plagioclase and feldspar with melting points of ~1200 °C, consistent with a temperature range of 1155–1290 °C, as estimated by Thy *et al*.^56^ for biomass glass. We observed no melted, high-temperature mineral grains that melt at >2000 °C, as are found in AH glass. This suggests that the biomass glass investigated did not form the same way as AH glass.

Arguing for a non-impact source of carbon-rich material from YDB layers, Scott *et al*.^57^ and van Hoesel *et al*.^58^ used reflectance-inferred temperatures to claim that this material was produced by low-temperature wildfires (Appendix, Text S2). However, a search of the literature on natural wildfires reveals no reports that these natural events are capable of melting sediment. Furthermore, low reflectance values do not preclude an impact event. For example, Garde *et al*.^59^ investigated carbon associated with shocked quartz and melted high-temperature microbreccia grains from the Hiawatha Crater. They reported reflectance measurements of Hiawatha carbon that yielded inferred temperatures of ~300°–600 °C, even though the melting points of impact glass are typically many thousands of degrees higher (inferred temperature: >1200 °C).

To further investigate this carbon issue, we measured values for (i) man-made activated carbon; (ii) charcoal found within high-temperature trinitite atomic glass; (iii) charcoal from the Tunguska fireball layer; and (iv) carbon spherules embedded in or closely associated with meltglass from 3 YDB sites. The known temperature differentials ranged up to ~844 °C higher than the temperature inferred by reflectance (Appendix, Table S6). These results indicate that charcoal reflectance measurement is accurate for normal fires but inaccurate for highly transient, high-temperature cosmic impact events.

Measurements of remanent magnetism for two lightning-produced fulgurites and one sample of volcanic obsidian were found to display strong magnetization. Also, three samples of industrial slag displayed a wide range of remanent magnetism, reflecting differing magnetic environments as they cooled. None of the samples contained remanent magnetism similar to that of AH glass.

FTIR results revealed that the water content in AH glass is much lower than typical anthropogenic, volcanic, and biomass glasses, which form at <1500 °C (Fig. 16b**)** (Appendix, Text S5). Therefore, it is highly unlikely that high-temperature AH glass was produced by these three processes. Also, the age precludes anthropogenesis, because AH villagers lacked the technology to make glass. In addition, AH meltglass was deeply buried in well-dated, sealed strata that preclude modern human contamination. AH glass has similar water content to fulgurites, but the latter are morphologically dissimilar to AH glass^8^, and so, lightning can be eliminated as a formation mechanism. Most conclusively, fulgurites display intense remanent magnetism, as discussed above, whereas magnetism levels in AH glass are consistent with exposure only to Earth’s ambient field.

We also investigated anthropogenic meltglass, called trinitite, produced during the 1945 Trinity nuclear airburst near Socorro, New Mexico^8,60,61^. Although no atomic detonations have occurred near Abu Hureyra, the meltglass from the Trinity airburst offers an opportunity for comparison to AH glass. Trinitite contains melted quartz grains that are morphologically indistinguishable from the melted quartz in AH glass, and analyses showed that some quartz grains in trinitite melted at ~1720 °C (Appendix, Fig. S23); and others apparently boiled at ~2230 °C. These melted quartz grains in trinitite appear identical to melted quartz grains in AH glass (e.g., Appendix, Fig. S9).

SEM-EDS investigation shows that trinitite also contains abundant Fe-rich globules (Appendix, Fig. S24). As with AH glass, the globules typically occur on outer surfaces of trinitite or on the inner surfaces of vesicles and have not been observed within the trinitite matrix. Three blebs of Fe silicides (FeSi, Fe_2_Si, and Fe_3_Si), and native Si were identified within a few microns of each other on samples of trinitite glass (Appendix, Fig. S25). Coexisting globules of reduced native Fe and magnetite were also observed separated by less than a few microns. The more common green-colored trinitite commonly contains no detectable silicides, and instead, the silicides were observed in medium-gray to dark-gray samples. SEM-EDS spectra indicate that gray trinitite contains abundant carbon, in contrast to the clear to green trinitite that contains little or no detectable C. We infer that the high-carbon content promoted the formation of silicides in trinitite, as well as in AH glass.

The only known natural dynamic events that produce Fe silicides are cosmic impacts, lightning strikes, and nuclear detonations, all of which share abnormally high temperatures, reducing environments, and rapid cooling. The silicides in trinitite samples formed both in bubble cavities and on the outside of silicate melt spherules, just as they did in AH glass. Fe silicides in AH glass have characteristics most consistent with those of Fe silicides and native Fe found in trinitite, where bubble cavities were closed systems. Thus, the requisite formation conditions of AH glass appear most similar to those in nuclear detonations that produced trinitite.

Remanent magnetism measurements were also made on 2 samples of atomic glass from underground bomb tests and one sample of trinitite, all of which showed a moderate increase in remanent magnetism over background values. These measurements are unlike those for AH glass.

### Comparison to known impact meltglass

We used SEM-EDS and microprobe analyses to compare AH glass to melted materials from the Australasian tektite field; Dakhleh Oasis, Egypt; Chasico, Argentina; Darwin Crater, Australia; Houghton Crater, Canada; Meteor Crater, Arizona; Monturaqui Crater, Chile; Ries Crater, Germany; and Zhaminshin Crater, Kazakhstan.

For the Australasian tektite field, SEM-EDS analyses were performed on melted quartz grains in a Muong Nong tektite (Appendix, Fig. S26), an aerodynamically-rounded piece of impact-related meltglass from the Australasian tektite field, the world’s largest known cosmic impact strewn field, covering 10–30% of Earth’s surface^8^. The tektite displayed melted and bubbled quartz grains that had lost most of their original grain integrity and diffused into the surrounding glass matrix (Fig. 26), most likely at temperatures near the boiling point of quartz at 2230 °C. These melted quartz grains are very similar to melted quartz grains in AH glass (e.g., Appendix, Fig. S9).

Impact-related melted rocks, called “Dakhleh glass,” occur over an area of ~400 km^2^ in and around Dakhleh Oasis, Egypt^62^ as a result of a proposed cosmic airburst or ground impact event ~150,000 years ago. Our investigations of this meltglass reveal that some vesicles of Dakhleh glass contain rounded and distorted zircon grains, indicative of high-temperature melting (Appendix, Fig. S27). Dakhleh zircons are similar to melted zircon grains in AH glass (Fig. 7), demonstrating that impact events can produce zircon morphologies like those at Abu Hureyra.

Our investigations also show that meltglass samples from Meteor Crater, Arizona contain melted chromite grains (Appendix, Fig. S28). This demonstrates that cosmic impacts can produce melted chromite grains that are similar to those in AH glass. For comparison, Meteor Crater was a surface impact by a NiFe-rich impactor, whereas we infer that Abu Hureyra involved the airburst of a small comet or asteroid, accompanied by likely surface impacts by small fragments of the aerial detonation. Fe-rich inclusions commonly occur in vesicles of AH glass, and SEM-EDS analyses show similar inclusions in impact-related meltglass from Meteor Crater, Arizona; Dakhleh Oasis, Egypt^62^; and Chasico, Argentina^63^, where the meltglass is called Argentine escoria (Appendix, Fig. S29). The similarities to inclusions in AH glass are consistent with a common origin in some type of cosmic impact event.

Measurements of remanent magnetism were acquired on material from known impact events, including 4 tektites from the Australasian tektite field and one sample each from the cosmic airbursts or impacts at Dakhleh Oasis, Egypt; Chasico, Argentina; Darwin Crater, Australia; Houghton Crater, Canada; Meteor Crater, Arizona; Monturaqui Crater, Chile; Ries Crater, Germany; and Zhaminshin Crater, Kazakhstan. Field strength measurements ranged from weak to normal to strong, as compared to normal measurements for AH glass (Appendix, Text S6, Tables S8 and S9).

For known impact glass, the water content can be very low H_2_O (~80 ppm)^64^ but can be higher at ~0.46 wt.% to 24 wt.%^65^. For tektites, one of the longest recognized and most diagnostic geochemical features is that they typically contain less than 500 ppm of H_2_O^52,66,67^. In this study, water content for tektites from the Australasian tektite field and meltglass from Dakhleh Oasis and Ries Crater ranged from 78–1036 ppm, which is wider than the range of 222–460 ppm for AH glass.

### Similar proxies from other YDB sites

We compared AH proxies to those from other YDB sites, using light microscopy, SEM-EDS, neutron activation analyses, remanent magnetism, and abundances of platinum, iridium, nickel, cobalt, chromium, and/or AH glass.

Remanent magnetism for YDB material from the YDB sites at Blackville and Melrose displayed levels of remanent magnetism (demagnetized, normal, or moderate) that are similar to values from Abu Hureyra. The measurements suggest that the spherules and meltglass from Abu Hureyra, Blackville, and some from Melrose did not form as the result of lightning.

Fe-rich minerals from Abu Hureyra show similar dendritic morphologies as those in YDB meltglass from Blackville, South Carolina and Melrose, Pennsylvania (Appendix, Fig. S30). For meltglass from these YDB sites, Bunch *et al*.^8^ found geochemical differences, suggesting the glass formed from local sediments, although for all sites, they inferred formation temperatures of ≥1590 °C, the melting point of magnetite. However, unlike for AH glass, Fe-rich minerals were also found inside sectioned samples of meltglass at these localities. Because the degree of dendritic growth is a function of time, these internal Fe-rich textures suggest that the glass cooled more slowly than at Blackville and Melrose than at Abu Hureyra. Native iron (Fe^0^) in meltglass from Melrose (Fig. 32c) indicates low ƒO_2_, suggesting that the meltglass formed in an oxygen-deficient atmosphere, as also appeared to have occurred with AH glass.

A suite of elements, including platinum, iridium, nickel, cobalt, and/or chromium in variable abundances, has been previously reported in spherules, meltglass, and sediment at ~30 other YDB sites across three continents, including Greenland^1,9,15,17–19,68–72^. The results from Abu Hureyra closely match these other studies, which attribute those enrichments to a cosmic impact event, derived from the impactor and/or from target rocks.

## Conclusions

In this study, material excavated at Abu Hureyra was investigated and the characteristics were compared to those from many other sources (Table 2). High-temperature minerals are commonly associated with AH spherules and with the outer surfaces of AH meltglass fragments and the inner surfaces of vesicles, but it is noteworthy that no high-temperature grains (e.g., quartz, zircon, chromite) were ever observed on interior broken surfaces. This suggests that internal glass temperatures were much lower than ambient external temperatures (>1200 °C). From laboratory heating experiments, we infer the following. (1) Between ~1100° and 1550 °C, some original minerals became partially melted (e.g., chlorapatite and pyrrhotite) and show melt flow, minor vesiculation, and elemental diffusion into the AH glass matrix. (2) Between ~1500° and 1700 °C, other minerals (including magnetite and titanomagnetite) display partial to complete melting. (3) Between ~1700 and 2000 °C, melted or partially melted minerals include quartz and zircon, and titanium sulfide. (4) Between ~2000 and 2600 °C, monazite, chromite, and chromium-rich magnetite melted and possibly boiled. (5) Some minerals in all categories apparently formed under highly reducing conditions (Table 1**)**. We infer that internal meltglass temperatures reached only ~1250 °C, whereas ambient atmospheric temperatures rose to at least 1750 °C and most likely to >2600 °C, based on the boiling points of chromite and magnetite.

**Table 2:** Array of evidence analyzed from Abu Hureyra and other sites.

Evidence Analyzed	Abu Hureyra YDB	Blackville YDB	Melrose YDB	K-Pg impact	Tektites fields	Tunguska/Dakhleh	Atomic–trinitite	Anthropogenesis	Biomass glass	Lightning	Volcanism	Wildfires
Magnetic spherules	Y	Y	Y	Y	Y	Y	Y	Y	-x-	Y	Y	-x-
Silicate spherules	Y	Y	Y	Y	Y	Y	Y	Y	Y	Y	Y	-x-
Meltglass	Y	Y	Y	Y	Y	Y	Y	Y	Y	Y	Y	-x-
Melt accretions	Y	Y	Y	Y	Y	Y	Y	Y	Y	Y	Y	-x-
Aerodynamic shaping	Y	Y	Y	Y	Y	Y	Y	Y	Y	Y	Y	-x-
Lechatelierite	Y	Y	Y	Y	Y	Y	Y	Y	-x-	Y	-x-	-x-
Glass with plant parts	Y	Y	Y	Y	~Y	Y	Y	Y	Y	Y	Y	-x-
High-T minerals (>1800 °C)	Y	Y	Y	Y	Y	Y	Y	-x-	-x-	Y	-x-	-x-
Low O_2_ fugacity	Y	Y	Y	Y	Y	Y	Y	-x-	-x-	Y	-x-	-x-
Major biomass burning	Y	Y	Y	Y	~Y	Y	-x-	-x-	-x-	Y	Y	Y
Carbon spherules	Y	Y	Y	Y	~Y	Y	~Y	Y	Y	Y	Y	Y
Nanodiamonds	Y	Y	Y	Y	~Y	Y	~Y	-x-	-x-	~Y	-x-	-x-
Ir and Pt peaks	Y	Y	Y	Y	Y	Y	-x-	-x-	-x-	-x-	Y	-x-
Magnetism = ambient field	Y	Y	Y	Y	Y	Y	-x-	Y	Y	-x-	Y	Y
H2O content <0.05%	Y	Y	Y	Y	Y	Y	Y	-x-	-x-	Y	-x-	-x-
Widespread extinctions	Y	Y	Y	Y	Y	Y	-x-	-x-	-x-	-x-	-x-	-x-

Y = yes; -x- = no; ~Y = probable.

Shows the presence (“Y”), likely presence (“~Y”), or absence (“-x-”) of various lines of evidence in 12 types of glass. Abu Hureyra material, like that of other YDB sites, closely resembles material from known impacts and cosmic airbursts. Collectively, it is dissimilar to anthropogenic material, biomass glass, lightning-produced material, volcanic meltglass, and wildfire evidence.

We can exclude the following as potential formation mechanisms for AH glass: building fires; biomass or “haystack” fires; anthropogenic contamination; and lightning-induced melting (Appendix, Text S7). Instead, we infer that AH glass fragments resulted from the nearly instantaneous melting and vaporization of regional biomass, soils, and floodplain deposits followed by nearly instantaneous cooling. This scenario is supported by the presence of meltglass with flow marks and no apparent crystallization, unlike slow-cooling melted material. In AH glass, high formation temperatures followed by rapid cooling created oxygen-deficient minerals, such as native iron, native silicon, and alloys of Fe, Cr, Au, and Al. These materials are extremely rare under normal terrestrial conditions but are commonly formed during impact events.

The observed range of characteristics for AH glass (e.g., low water, high-temperature minerals, low remanent magnetism, and minerals with low oxygen fugacity) allow us to exclude all potential origins for AH glass, except cosmic impact-melted glass and tektites, with the latter offering the best match. Thus, the formation of AH glass appears to require the occurrence of an intense and sudden high‐temperature event similar to known tektite-producing impacts. The collective evidence is best explained by the hypothesis that at least one high-energy, high-temperature, hypervelocity event occurred near Abu Hureyra ~12,800 years ago, most likely an airburst possibly accompanied by ground impacts by impactor fragments.

The YDB hypothesis posits multiple airbursts/impacts across at least four continents^1,3,5,8–11,20,69,73–78^. These are proposed to have resulted from a one of a series of short-period, active comets known to break up frequently and to shed dozens to thousands of fragments that are 10 to 1000 m in diameter, each capable of producing catastrophic airburst/impacts, as discussed in detail in Napier^3,79^, Wolbach *et al*.^13,14^, and Pino *et al*.^9^ (Appendix, Text S8). The largest cometary debris clusters are proposed to be capable of causing thousands of airbursts within a span of minutes across one entire hemisphere of Earth. An encounter with such a million-km-wide debris cluster would be thousands of times more probable than a collision with a 100-km-wide comet or a 10-km asteroid. The YDB hypothesis proposes this mechanism to account for the impact at Abu Hureyra and coeval impacts across >14,000 km of the Northern and Southern Hemispheres.

## Methods

### Laboratory melting experiments

To determine the melting point of Abu Hureyra sediment, a typical sediment sample from a pithouse in Trench E was heated in either alumina or graphite crucibles in steps of approximately 100 °C beginning at 1000 °C. Typically, ~1 g of each sample (Abu Hureyra bulk sediment or plant-imprinted glass) was placed in an alumina crucible (99.8 wt.% Al_2_O_3_, CoorsTek) and inserted into a box furnace preheated to near the temperature of interest. Temperatures were monitored using a type B thermocouple inside the furnace chamber. After 10 minutes following hot-insertion, the crucible was removed and placed on an insulating surface, where it cooled rapidly. J.P.K. and M.W.G. performed the analyses.

### SEM-EDS analyses

Standard practices were followed for samples investigated with electron microscopy (Appendix, Methods). M.L., A.V.A., T.E.B., J.C.W., P.H., G.K., J.W., A.W., and Jake Lowenstern performed the analyses and interpreted the results.

### Carbon reflectance

Standard practices were followed for preparation and measurements of reflectance for carbon samples (Appendix, Methods). P.H. performed the analyses and interpreted the results.

### Remanent magnetism

Seven samples from Abu Hureyra and other sites were selected for magnetic measurements. They were weighed and affixed to a holder designed to measure the variation of the magnetization vector through stepwise laboratory-induced demagnetization in three perpendicular axes. The instrument measures weak magnetic fields due to a superconducting quantum interference device containing Josephson junctions made by 2 G Enterprises. The noise level of this instrument was 1e-8 A/m of the measured magnetic moment. The magnetic acquisition was acquired with pulse magnetizer ASC Model IM-10-30. G.K. performed the analyses and interpreted the results. G.K. performed the analyses and interpreted the results.

### Transmission FTIR and water content

Standard practices were followed for all samples investigated by Fourier-transform infrared spectroscopy (FTIR) (Appendix, Methods). Jake Lowenstern performed the analyses and interpreted the results.

### Electron microprobe analyses

Standard practices were followed for all samples investigated with by electron microprobe (Appendix, Methods). Jake Lowenstern performed the analyses and interpreted the results.

## Supplementary information

Supplementary Information.

## Data Availability

All data are given in the tables, figures, and SI. Regarding samples, the Abu Hureyra site is now under the waters of Lake Assad, and after >40 years, there is no more bulk sediment available from the YDB layer. However, sediment is available from layers above and below it and pieces of YDB meltglass are archived in London and available through Andrew M.T. Moore (amtmoore@gmail.com).
