# Supplementary material for: Evidence of Cosmic Impact at Abu Hureyra, Syria at the Younger Dryas Onset (~12.8 ka): High-temperature melting at >2200 °C
Source: Sci Rep. 2020 Mar 6;10:4185. doi: 10.1038/s41598-020-60867-w (PMC7060197; doi:10.1038/s41598-020-60867-w)
Supplement: Supplementary file 1 — Supplementary Information. [file 41598_2020_60867_MOESM1_ESM.pdf]

Evidence of Cosmic Impact at Abu Hureyra, Syria at the Younger Dryas Onset (~12.8 ka): High-temperature melting at >2200°C

Andrew Moore, James P. Kennett, William M. Napier, Ted E. Bunch, James C. Weaver, Malcolm LeCompte, A. Victor Adedeji, Paul Hackley, Gunther Kletetschka, Robert E. Hermes, James H. Wittke, Joshua J. Razink, Michael William Gaultois, Allen West*.

SUPPORTING INFORMATION: Images

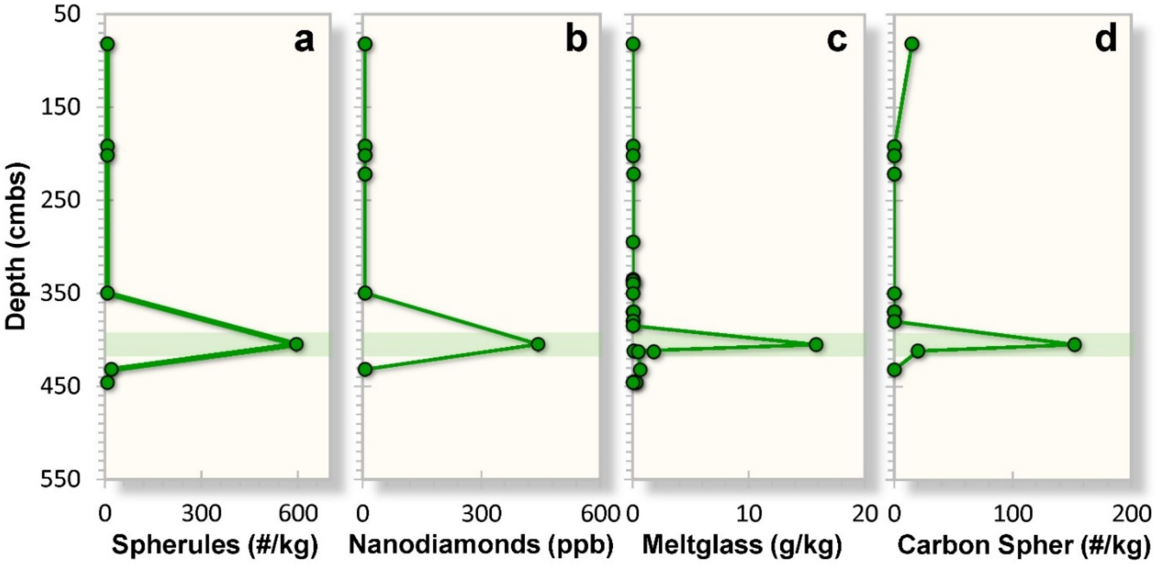

Figure S1. Peak concentrations of inferred impact-related proxies at Abu Hureyra, Syria. **(a)** Ca-Si-rich and Fe-rich spherules[1]; **(b)** nanodiamonds[2]; **(c)** high-temperature meltglass[1]; and **(d)** fire-related spherules composed of pure carbon[2,3].

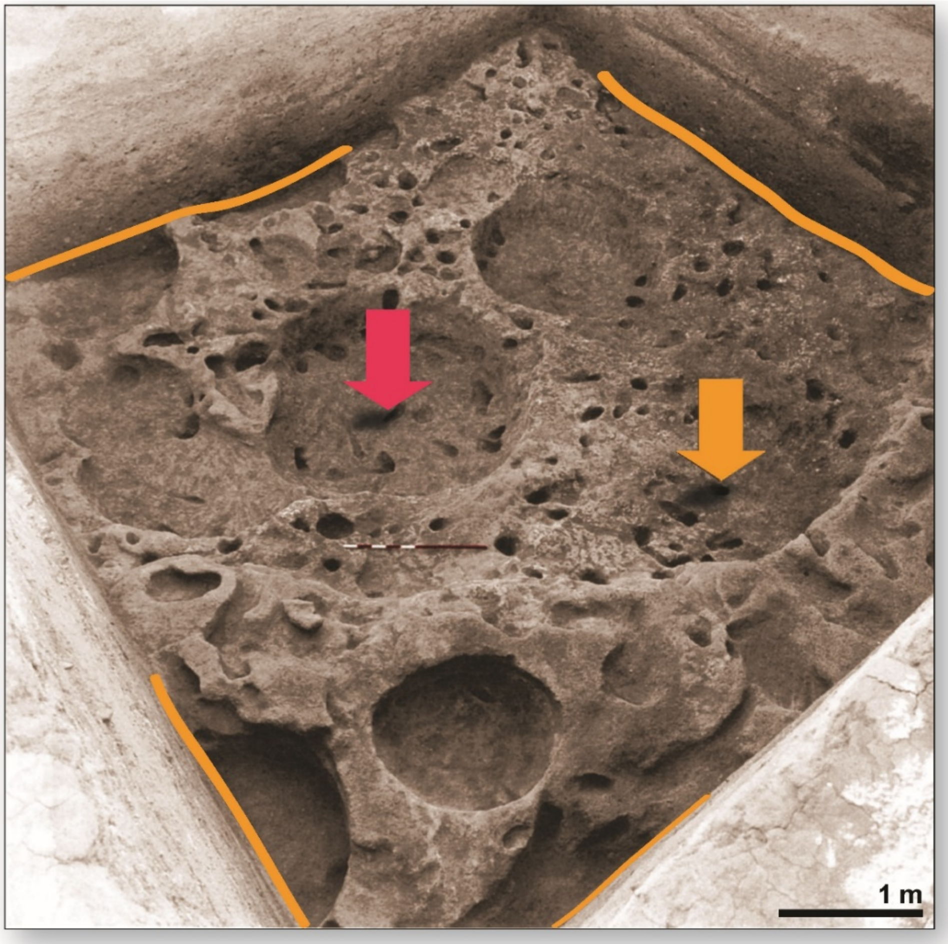

Figure S2. Photograph of Abu Hureyra occupational surface, dating to ~12,825 ± 55 cal BP[4]. Wattle-and-daub huts typically enclosed multiple small, hand-dug round pits, each less than a few meters across. The main interior room of a pit-house (red arrow) and charcoal-rich outside work area (orange arrow) contained abundance peaks in spherules, nanodiamonds, carbon spherules, meltglass, and platinum. Note that the surface contains numerous small holes, some having been dug by the villagers, suggesting considerable reworking of sediment. Orange lines along walls indicate the strata that were deposited during the onset of YD climate change. Figure adapted from Figure 5.12 in Moore et al.[5].

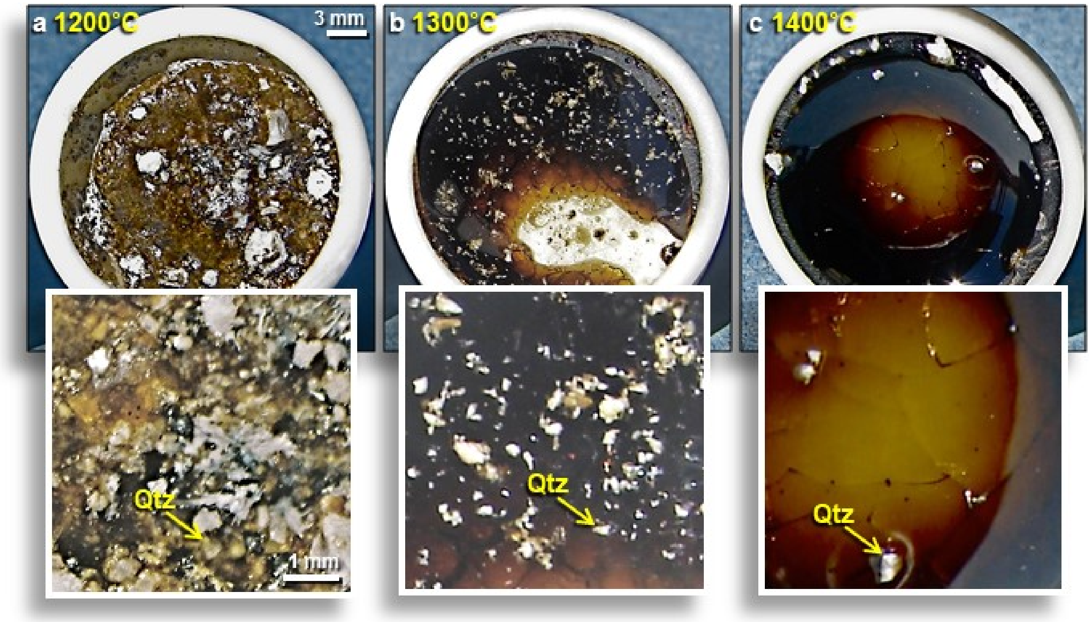

Figure S3. Results of furnace experiments. (a) At 1200°C, most AH bulk sediment melted, encapsulating existing refractory grains such as quartz and zircon. **(b)** By 1300°C, the fine-grained, clayey sediment melted and many small grains began to melt. **(c)** By 1400°C and continuing to 1700°C, progressively more detrital grains melted, and at 1700°C, some larger grains still survived. Heating experiments used Abu Hureyra bulk sediment from level 435, sample ES15, 395 cm depth.

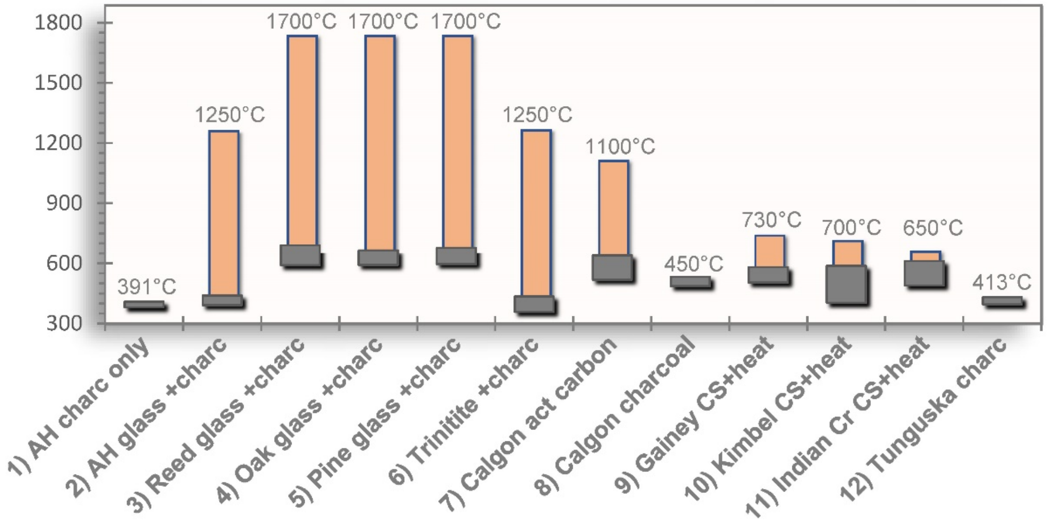

Figure S4. Charcoal temperature comparisons from reflectance measurements for different organic materials: #1 = loose Abu Hureyra charcoal extracted from AH sediment; #2 = charcoal embedded within Abu Hureyra meltglass; bars #3 through #5 = glass made from reeds, oak, and pine; bar #6 = charcoal within trinitite; bars #8 through #9 = Calgon activated carbon and source charcoal; bars #9 through #11 = YDB carbon spherules; and bar #12 = charcoal from Tunguska sediment. Gray rectangles represent the range of temperatures inferred from our reflectance percentages. Orange bars represent measured/inferred minimum formation temperatures.

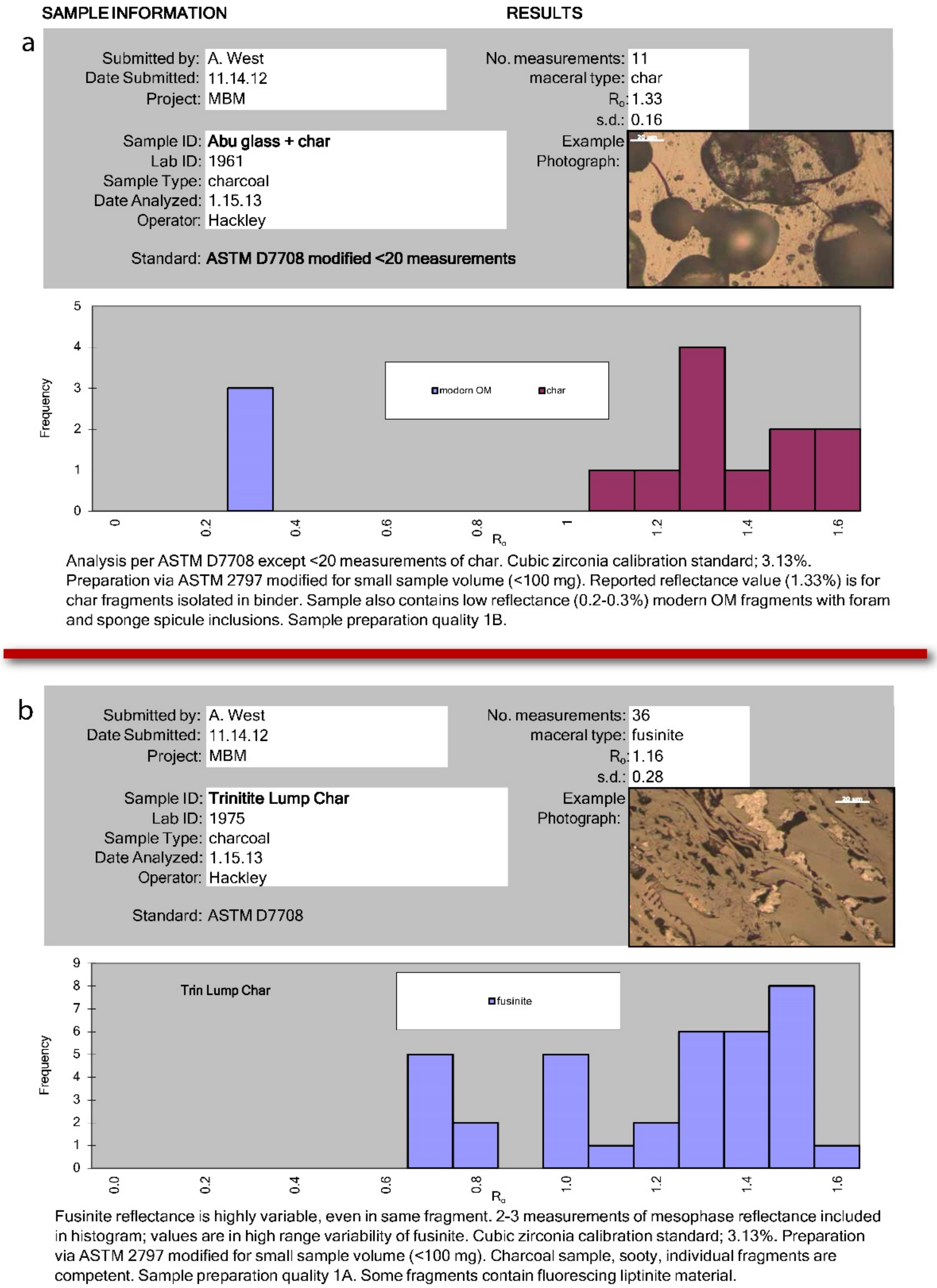

Figure S5. Reflectance results for (a) Abu Hureyra charred carbon that was fully encapsulated in AH meltglass. Inferred temperature of >1250°C, the approximate melting point of local sediment. Reflectance-inferred temperature was ~421°C, a difference of >829°C. Samples are from YDB level 445, sample E301, 405 cm; **(b)** Charcoal splattered with trinitite melted at >1250°C, the approximate melting point of local sediment. Reflectance-inferred temperature was ~406°C, a difference of >844°C.

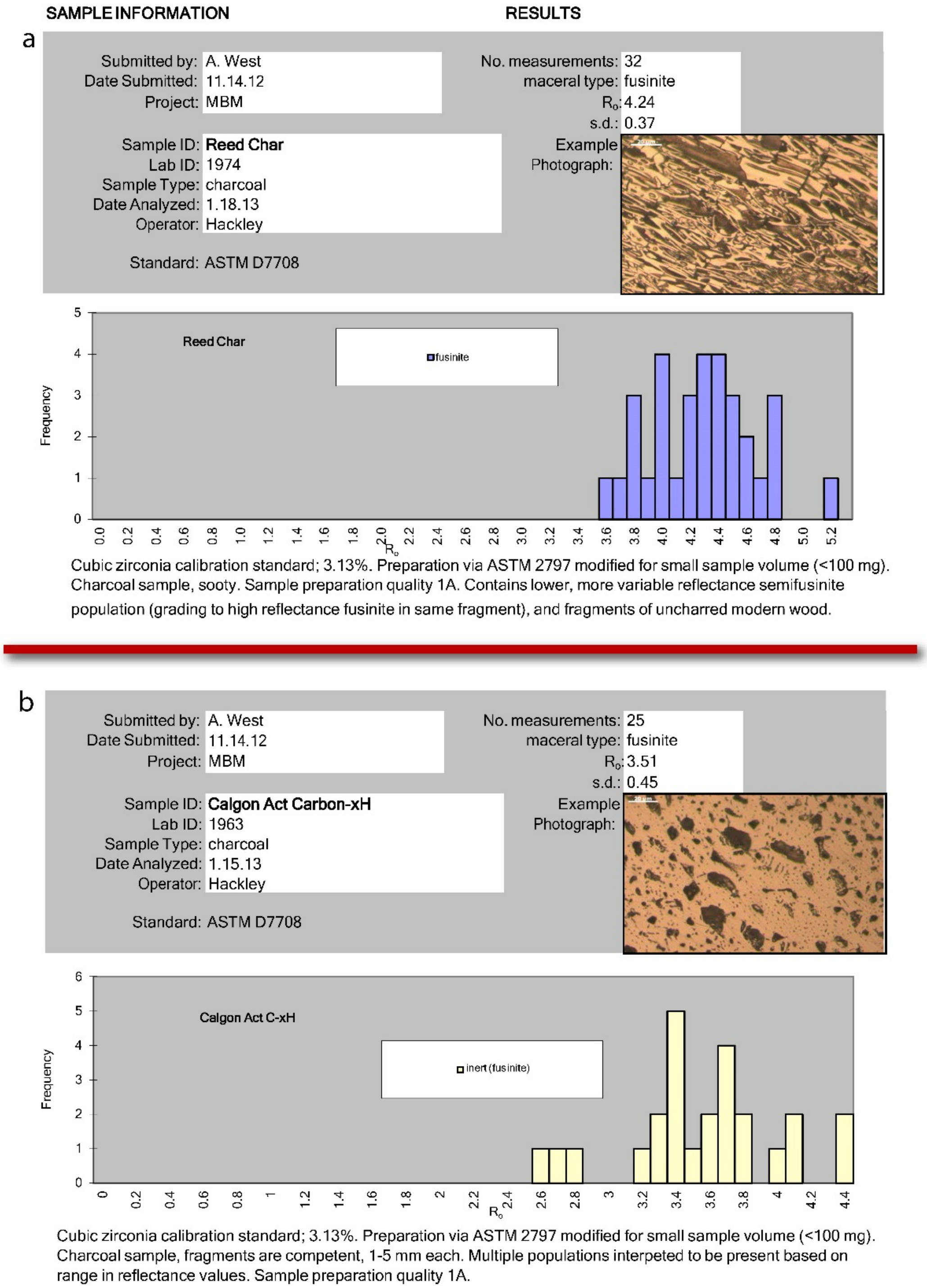

Figure S6. Reflectance results for (a) Charred carbon in meltglass produced from incinerating reeds under laboratory conditions to produce carbon-infused glass at temperatures of >1700°C. Reflectance-inferred temperature was ~632°C, a difference of >1068°C. **(b)** Calgon-brand activated carbon produced at temperatures of >1100°C. Reflectance-inferred temperature was much lower at ~585°C, a difference of >515°C.

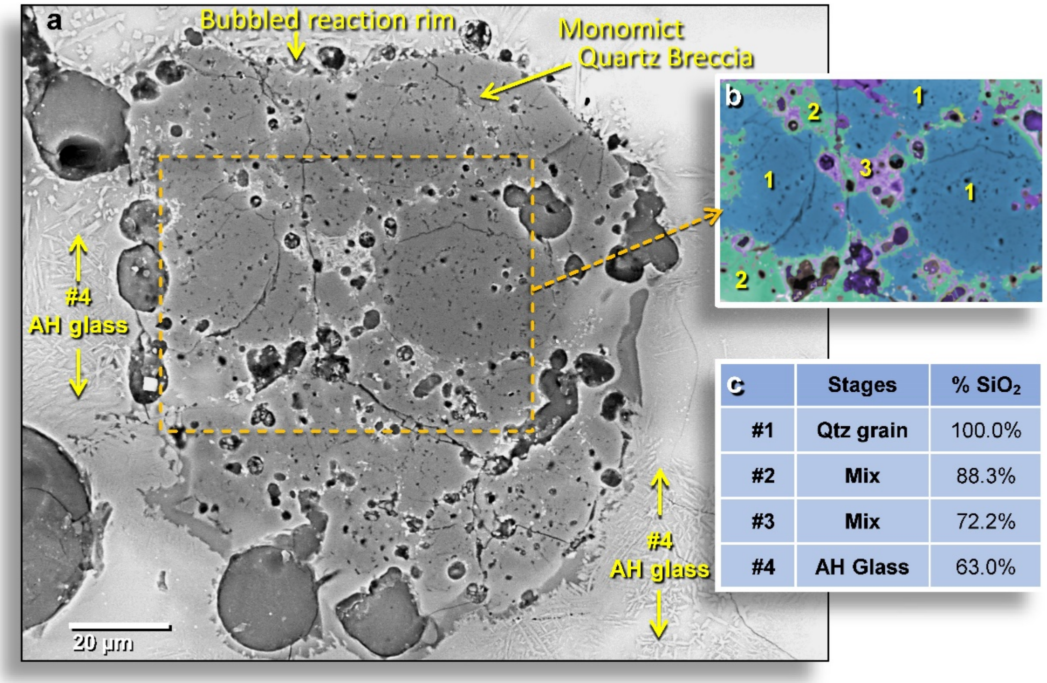

c	Stages	% SiO_2
#1	Qtz grain	100.0%
#2	Mix	88.3%
#3	Mix	72.2%
#4	AH Glass	63.0%

Figure S7. SEM images of quartz grain. (a) ~125-µm-wide brecciated quartz grain on an inner wall of AH glass vesicle. Darker gray central portion is monomict quartz, i.e., it formed from a single quartz grain. Note: bubbled reaction rim around grain breccia (arrow at top edge), indicative of high-temperature outgassing **(b)** Manually constructed EDS-based phase map with blue color representing melted grain and green and purple representing the diffusion of SiO_2 into the matrix. **(c)** Table of data measured by SEM-EDS for this grain, showing percentages of SiO_2 (range: 100 to 63.0 wt.%). From level 445, sample E301, 405 cm depth.

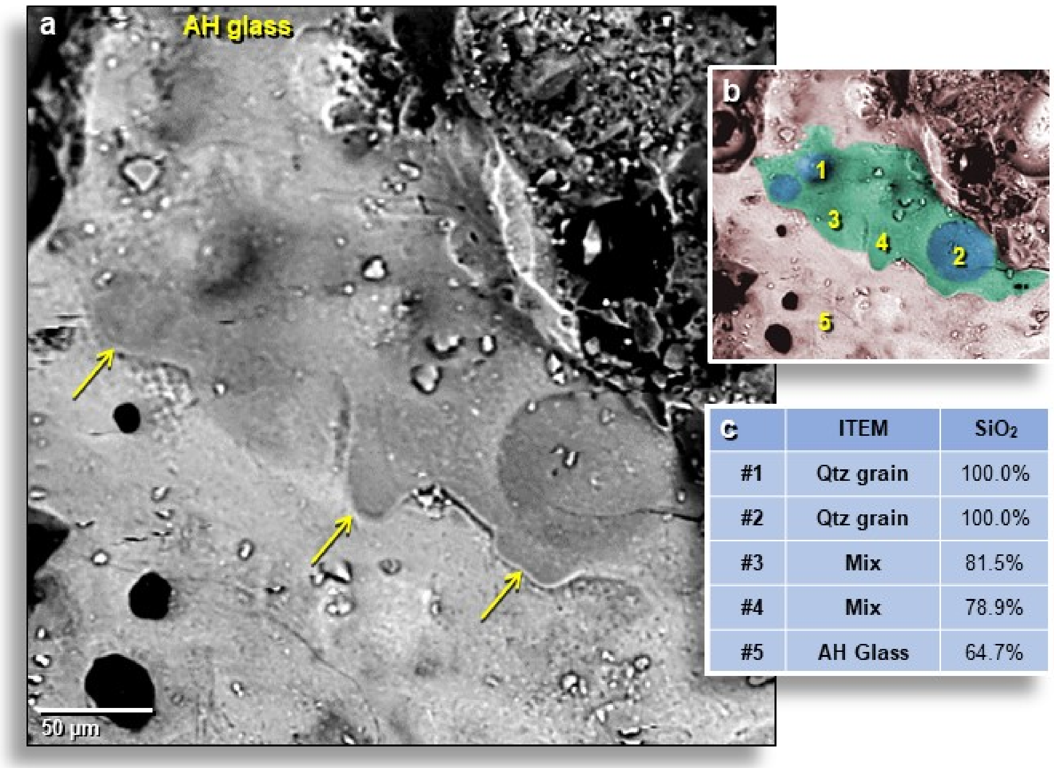

c	ITEM	SiO_2
#1	Qtz grain	100.0%
#2	Qtz grain	100.0%
#3	Mix	81.5%
#4	Mix	78.9%
#5	AH Glass	64.7%

Figure S8. SEM images of melted AH quartz grains. (a) A 260-µm-wide region of multiple quartz grains on the inner wall of an AH glass vesicle. Darker gray central portion of the image contains three almost fully melted quartz grains with no apparent remaining crystalline structure. Sinuous edge boundaries at arrows mark diffusion of melted quartz into the glass matrix. **(b)** Manually constructed EDS-based phase map with light red representing the aluminosilicate glass matrix, blue representing melted grains, and green representing the diffusion of SiO_2 into the matrix. **(c)** Table of data measured by SEM-EDS for this grain, showing percentages of SiO_2 (range: 100-64.7 wt.%). From level 435, sample ES15, 395 cm depth.

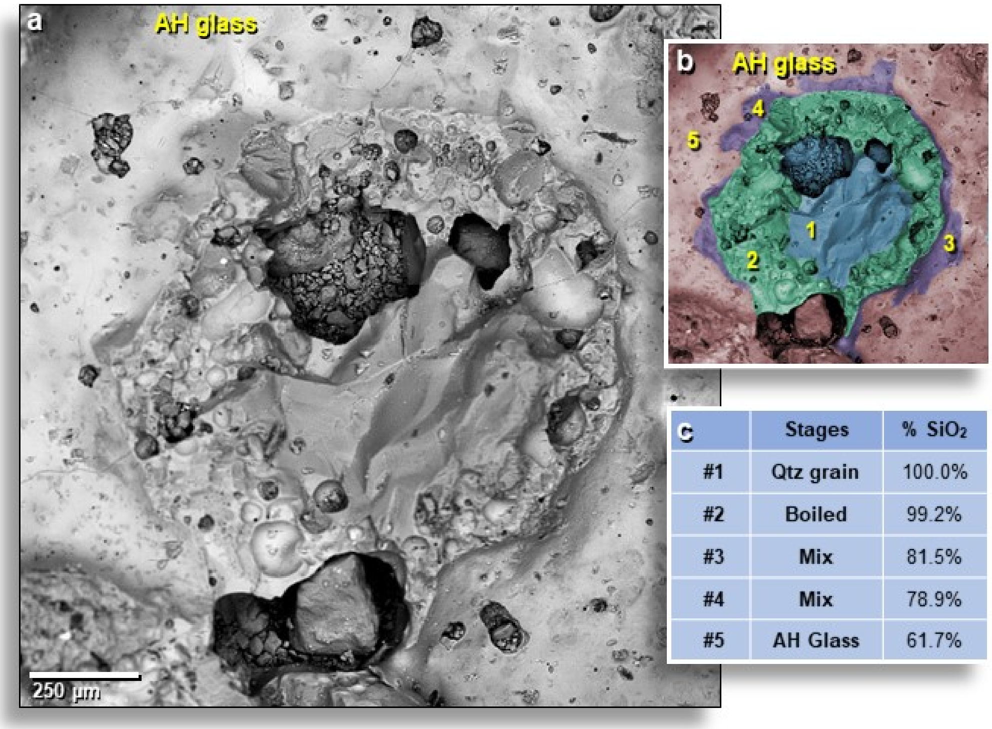

c	Stages	% SiO_2
#1	Qtz grain	100.0%
#2	Boiled	99.2%
#3	Mix	81.5%
#4	Mix	78.9%
#5	AH Glass	61.7%

Figure S9. SEM images of melted AH quartz grain. (a) The darker gray central portion of the image is of an unmelted 1040-µm-wide of a quartz grain remnant on the outer surface of AH glass. The highly vesicular area surrounding the grain is a combined 99.2 wt.% native Si and SiO_2. Vesiculation suggests the grain boiled at 2230°C. From level 435, sample ES15, 395 cm depth. **(b)** Manually constructed EDS-based phase map. Blue represents unmelted grain; green and purple represent progressive diffusion of SiO_2 into AH glass matrix in light red. **(c)** SEM-EDS data table for this grain, showing percentages of SiO_2 (range: 61.7 wt.% to 100 wt.%).

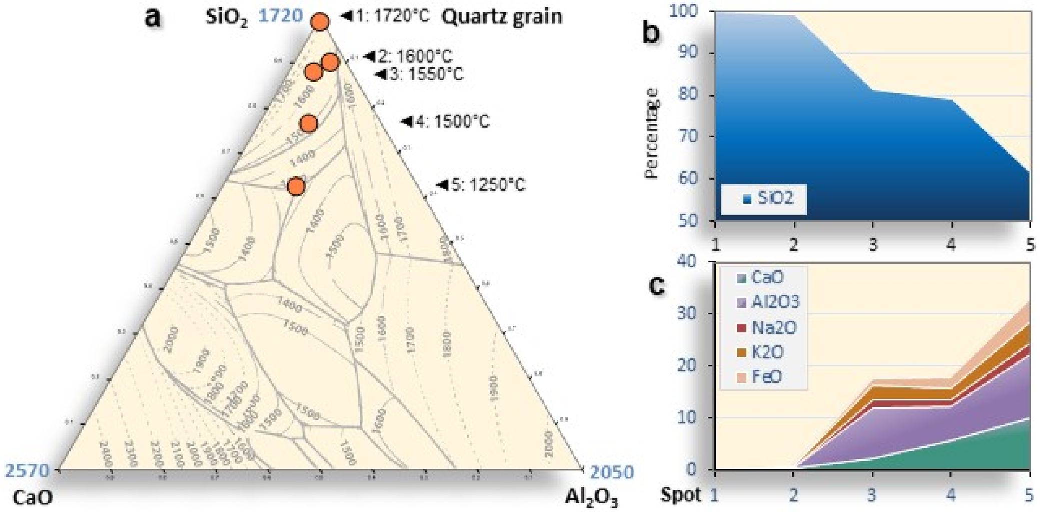

Figure S10. Compositional diagrams of quartz grain. (a) SiO_2-CaO-Al_2O_3 ternary phase diagram of five points from the table in **Fig. S9**. Inferred temperatures range from ~1720° to 1250°C under equilibrium conditions. Due to the fluxing action of oxides in the glass matrix, actual melting points are ~100° to 300°C lower. Ternary diagram based on Kracek[6]. **(b)** Area plot, showing percentages of SiO_2, ranging from 100 to 61.7 wt.%. **(c)** Area plot, showing percentages of major oxides other than SiO_2. AH glass is from level 435, sample ES15 at 395 cm.

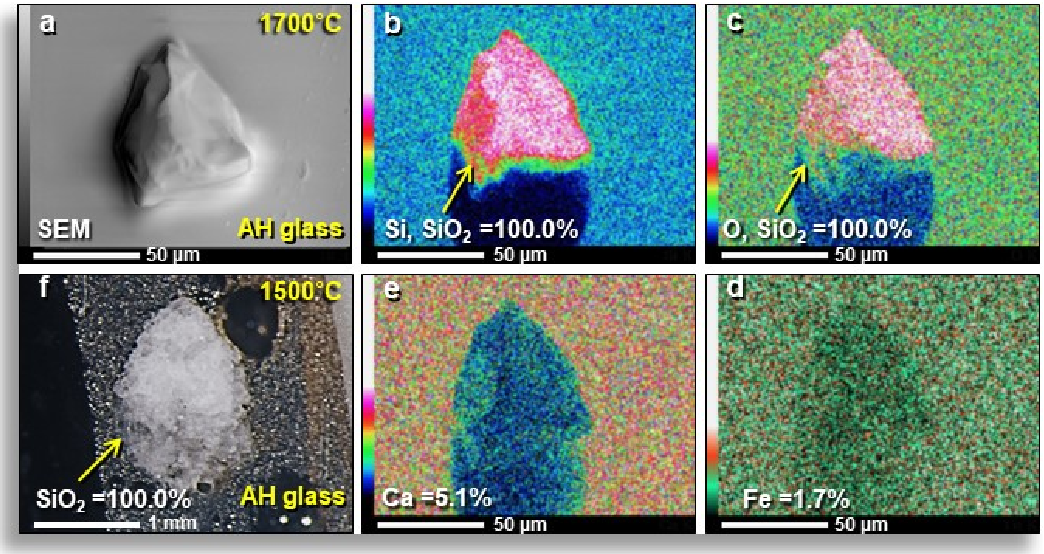

Figure S11. SEM-EDS elemental maps of quartz grains in furnace experiments. Laboratory heating experiments were conducted on Abu Hureyra sediment to determine the effects of heat on high-temperature minerals, such as quartz (SiO_2) with an equilibrium melting point of 1720°C and a boiling point of 2230°C. **(a)** SEM image of ~70-µm-wide monocrystalline quartz grain embedded into the surface of furnace-melted AH sediment heated to 1700°C. Grain has well-defined edges, showing little or no thermal alteration at high temperatures. **(b)-(e)** Single-element EDS maps with intensity scales with a colored gradient bar representing the relative wt.% at the lower left side of each panel. Results show that large grain is pure SiO_2 and confirm that quartz grain is embedded in the Ca-Al-Si-rich AH glass matrix. (**f,** bottom left**)** Photomicrograph of sectioned polycrystalline quartz grain heated to 1500°C with no apparent thermal alteration. Laboratory-melted bulk sediment from level 435, sample ES15, 395 cm depth.

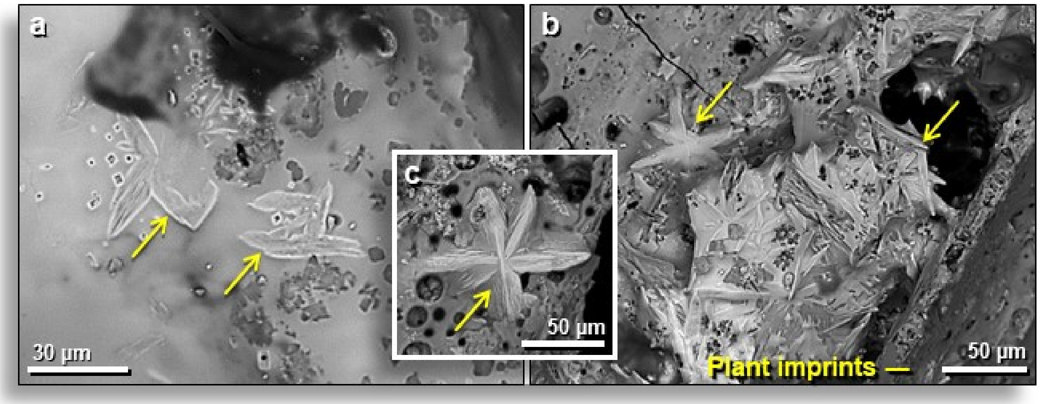

Figure S12. SEM images of calcium silicate ($CaSiO_3$, wollastonite) in AH glass. (a)-(c) SEM-EDS analyses indicate crystals are wollastonite. Panel b shows plant imprints on the AH glass at lower right. Star-shaped wollastonite crystal in Panel c is ~100 µm wide. Wollastonite crystals appear to form only on non-imprinted glass because calcium content was too low in plant remains. All images of outer surfaces of AH glass from level 445, sample E301, 405 cm depth.

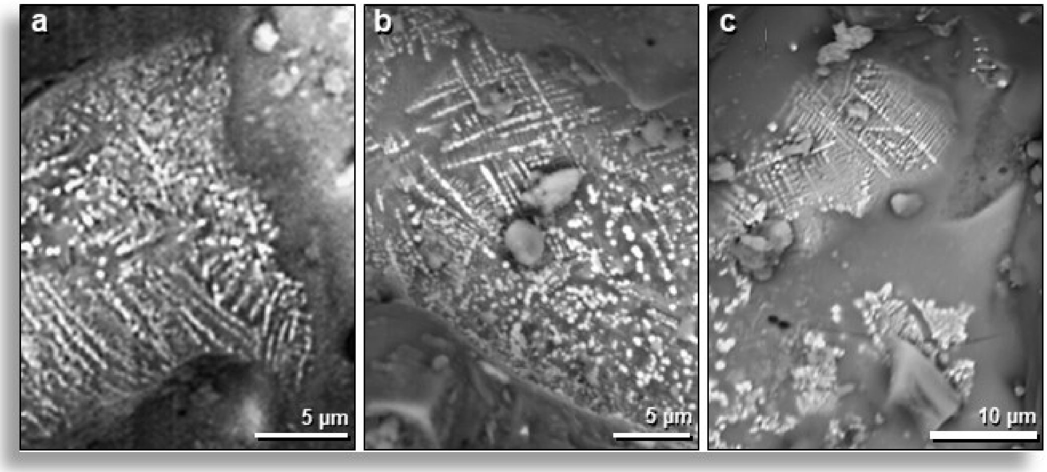

Figure S13. Iron crystals on AH glass. (a)-(c) SEM images of Fe crystals that form distinctive dendritic or feather-like quench patterns. All images from inner walls of AH glass vesicles from level 445, sample E301, 405 cm depth.

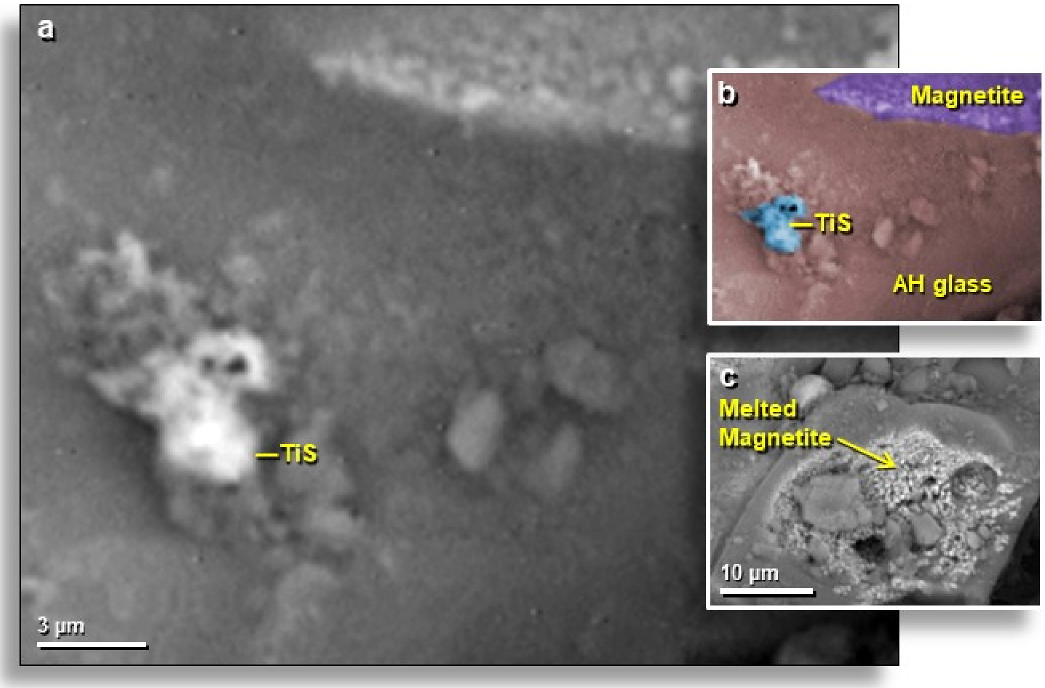

Figure S14. SEM images of titanium sulfide from Abu Hureyra. (a) ~5-µm-wide titanium sulfide (TiS) globule on the inner wall of AH glass vesicle. Small vesicles in the top right of TiS particle suggest that it was heated above its equilibrium melting point of 1780°C. **(b)** Manually constructed EDS-based phase map where TiS grain is in blue, melted magnetite grain is in purple, and AH glass matrix is in light red. **(c)** Melted 25-µm-wide magnetite grain within the same vesicle. From level 445, sample E301, 405 cm depth.

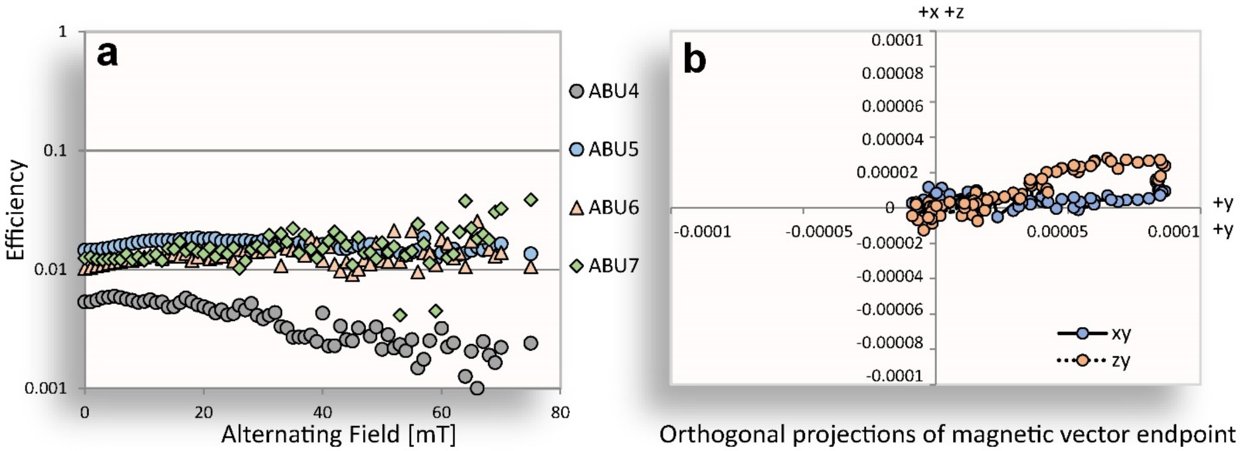

Figure S15. **Magnetic efficiency of four Abu Hureyra meltglass samples** (ABU4, ABU5, ABU6, ABU7). **(a)** Ratios of the stepwise demagnetized natural magnetic remanence and also stepwise demagnetized saturation magnetic remanence acquired at room temperature plotted against the stepwise demagnetization field used for demagnetization. **(b)** Orthogonal projections (solid-horizontal xz and empty-horizontal zy) of the magnetic vector endpoint during the demagnetization of its natural remanent magnetization. The sequence of points starts far from the origin (0 mT AF field) and ends at the origin (75 mT AF field). Units are in A/m.

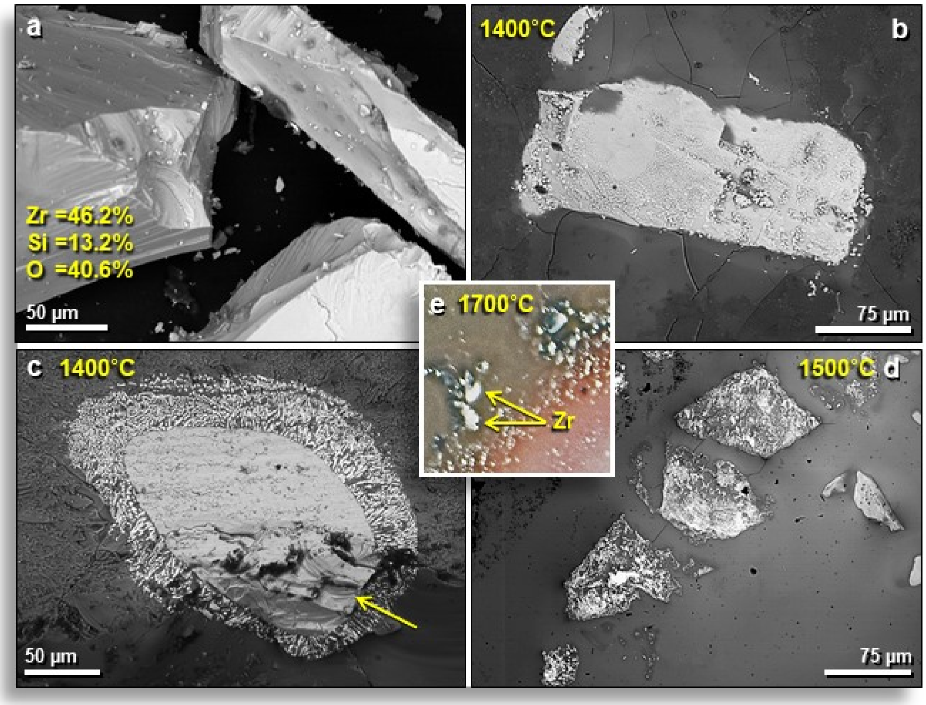

Figure S16. SEM images of zircon used in heating experiments. (a) Unheated, natural zircon grains. **(b)** At 1400°C, 275-µm-long zircon grain in laboratory-melted glass displayed slight surface alterations. **(c)** At 1400°C, a 250-µm-long zircon grain showed more thermal alteration but still maintained its original shape. **(d)** At 1500°C, 3 zircon grains ~120 µm wide show thermal alteration, while retaining their original shapes. Most but not all zircon <50 µm melted at ≤1500°C. **(e)** Photomicrograph of zircon grains (labeled Zr) heated to ~1700°C shows that the largest, white zircon grains generally retained their original shapes. Laboratory-melted bulk sediment from level 435, sample ES15, 395 cm depth.

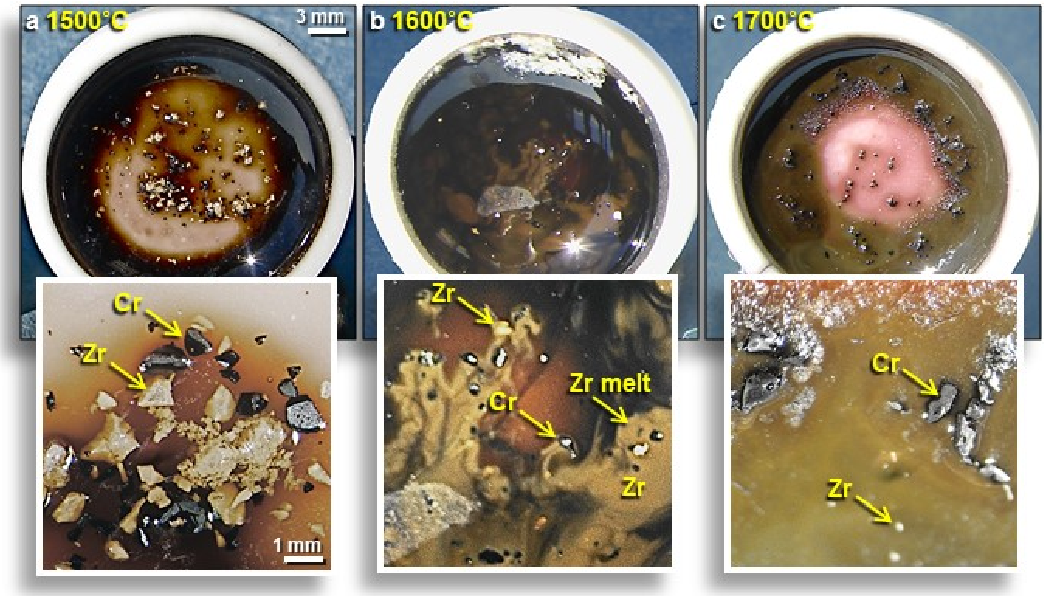

Figure S17. Heating experiments with chromite-spiked AH sediment. We conducted laboratory heating experiments to determine the response of these minerals to high temperatures, using ~1 g of AH sediment, mixed with ~10 wt.% crushed grains of chromite and zircon. At 1200ºC, the fine-grained, clayey sediment melted and enclosed numerous unmelted grains of black chromite, white zircon, clear/white quartz, and other detrital grains. At 1400ºC, a larger percentage of the bulk sediment melted, but there were still large numbers of unmelted grains visible in the dark, transparent glass. **(a)** At 1500°C, no detectable melting occurred for the added grains of chromite ($(Fe,Mg)Cr_2O_4$), labeled Cr. Limited edge melting occurred in zircon, labeled Zr. **(b)-(c)** At 1600° and 1700°C, no detectable melting occurred in chromite grains. For zircon, smaller grains melted completely and diffused into the aluminosilicate matrix at ~1600°C, but zircon grains approximately >100 µm showed moderate melting at ~1700°C. Under normal conditions, chromite crystals typically melt at equilibrium temperatures of ~2265°C[7]. Laboratory-melted bulk sediment from level 435, sample ES15, 395 cm depth.

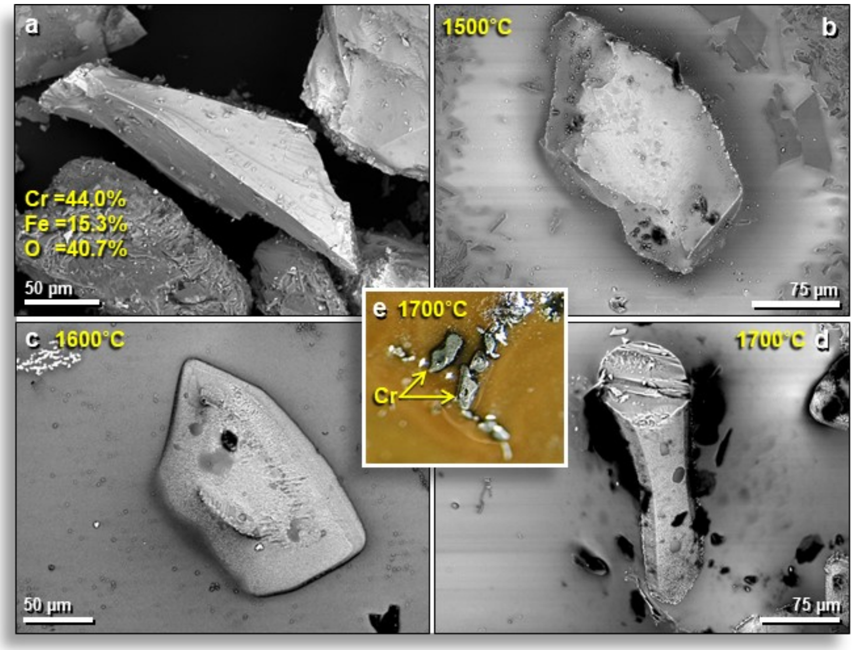

Figure S18. Images of chromite grains in heating experiments. (a) SEM image of an unheated, 235-µm-long grain of chromite ($(Fe,Mg)Cr_2O_4$). **(b)** At 1500°C, a 250-µm-long chromite grain embedded in the surface of laboratory-melted AH sediment showed minimal thermal alteration. **(c)** At 1600°C, a 165-µm-long chromite grain displayed moderate surface melting, but temperatures were insufficient to cause diffusion into AH sediment matrix. **(d)** At 1700°C, a 240-µm-long chromite grain displayed moderate surface melting with limited diffusion into the matrix. **(e)** Photomicrograph of partially melted chromite grains, labeled Cr, after heating to 1700°C. Some euhedral edges remain visible. Laboratory-melted bulk sediment from level 435, sample ES15, 395 cm depth.

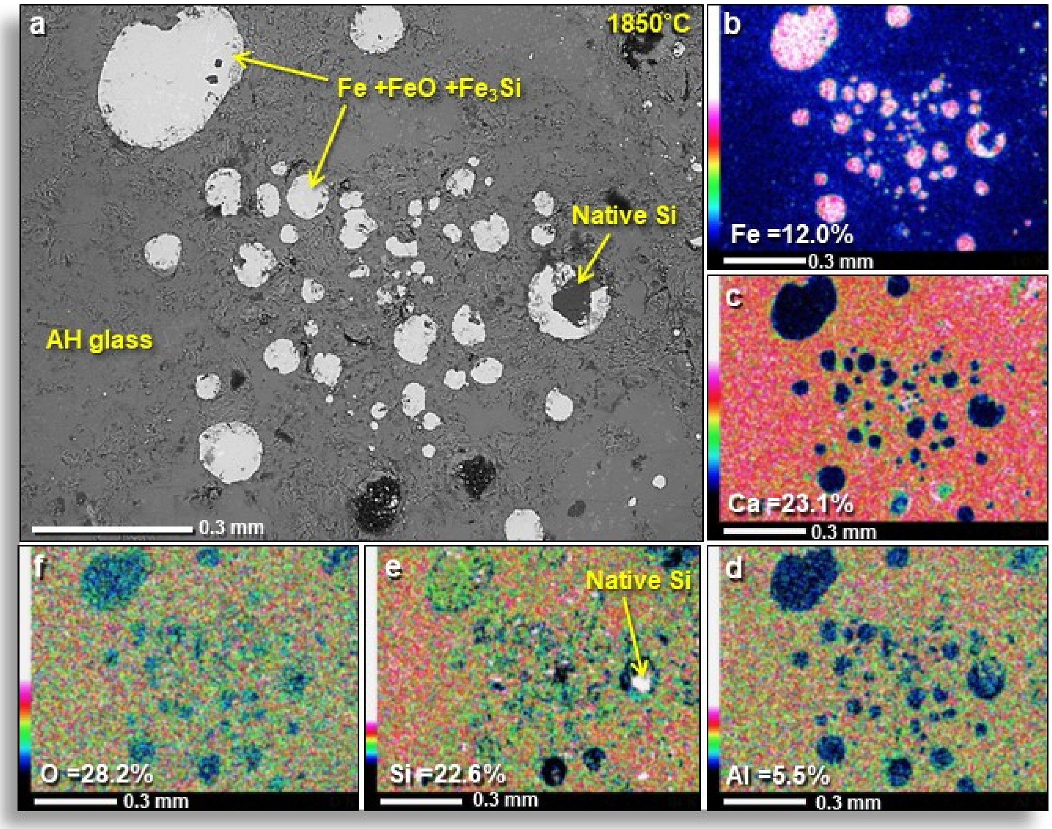

Figure S19. Fe-rich inclusions (globules) in AH glass from heating experiments. Equipment used included a Spark Plasma Sintering (SPS) furnace capable of reaching 1850°C. Graphite crucibles were used to mimic the hypothesized impact environment at Abu Hureyra that was carbon-rich because of vaporized vegetation. As with previous experiments, ~1 g of AH sediment was heated in ~100°C steps from 1000° to 1850°C. **(a)** SEM image of Fe-rich globules on the surface of the laboratory-melted glass. The maximum temperature was ~1850°C for several minutes. SEM-EDS indicates a composition of native Fe (Fe^0), reduced iron (FeO) and iron silicide (Fe_3Si), all of which reflect very low fO_2 and do not normally exist in nature. **(b)-(f)** Single-element EDS maps with intensity scales showing variations in the abundances of Fe, Ca, Al, Si, and O. Note that oxygen in these blebs has very low abundance in blue-black areas, indicating native Fe and native Si in some cases. The laboratory-melted glass is enriched in Ca, Al, and Si, the same as excavated AH glass. Laboratory-melted bulk sediment from level 435, sample ES15, 395 cm depth.

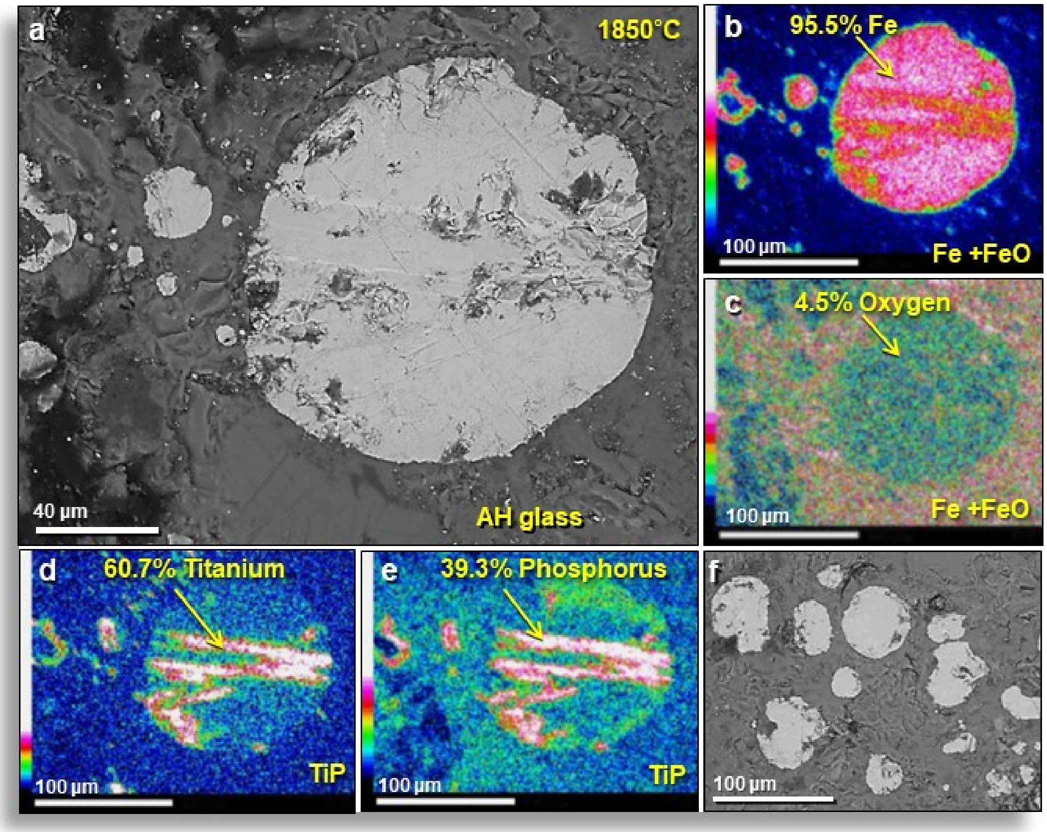

Figure S20. SEM images of iron globules from furnace experiments. (a) SEM images of globules composed of highly reduced iron. **(b)** Single-element (Fe) EDS map with intensity scale shows a rounded, flat 130-µm-wide globule of Fe (red). Pink area (arrow) is ~95.5 wt.% Fe, composed mostly of native Fe mixed with a small percentage of FeO. **(c)** Single-element (oxygen) EDS map with intensity scale of the same area containing ~4.5 wt.% O, confirming highly reduced Fe. **(d)** Single-element (titanium) EDS map with intensity scale showing streaks of Ti at 60.7 wt.% and **(e)** Single-element (phosphorus) EDS map with intensity scale showing P at 39.3 wt.%, indicating the crystallization of titanium phosphide (TiP) within the Fe globule. TiP, with a melting point of ~1400°C, was observed in heating experiments but not found in AH glass. **(f)** A field of Fe-rich globules. Laboratory-melted bulk sediment from level 435, sample ES15, 395 cm depth.

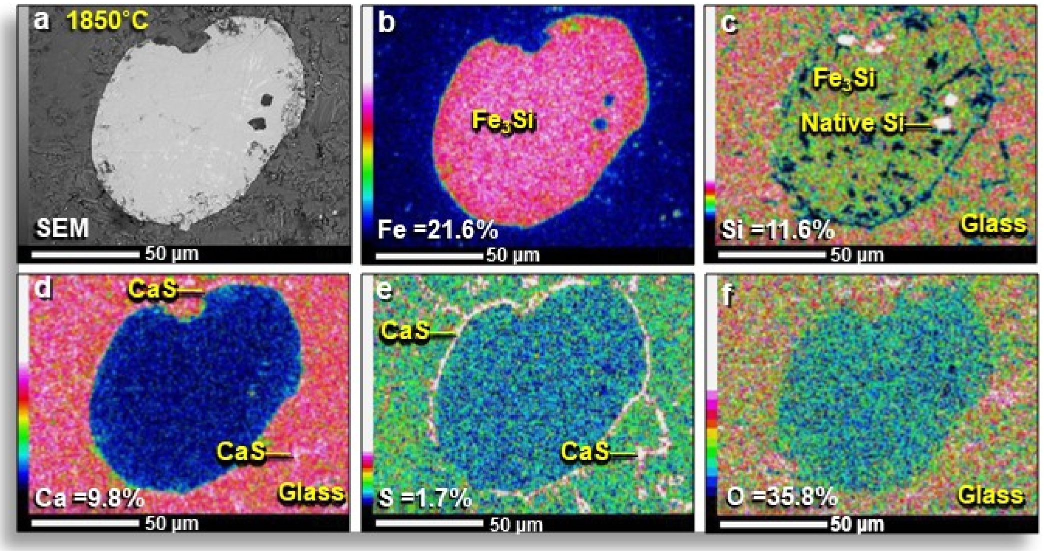

Figure S21. SEM images of iron silicide in furnace experiments at 1850°C. (a) ~110-µm-wide Fe-rich globule on the surface of laboratory melted glass. **(b)** Single-element (Fe) EDS map with intensity scale shows high Fe content in the center object. **(c)** Single-element (Si) EDS map with intensity scale. Fe content plus high Si in center object indicates the composition is iron silicide, Fe_3Si, as confirmed by SEM-EDS. White areas mark inclusions of native Si. **(d)** Single-element (Ca) EDS map with intensity scale. Ca content of AH glass is at ~9.8 wt.% **(e)** Single-element (S) EDS map with intensity scale showing white areas, representing calcium sulfide (CaS). **(f)** Single-element (O) EDS map with intensity scale indicating low oxygen content in Fe-rich globule. Laboratory-melted bulk sediment from level 435, sample ES15, 395 cm depth.

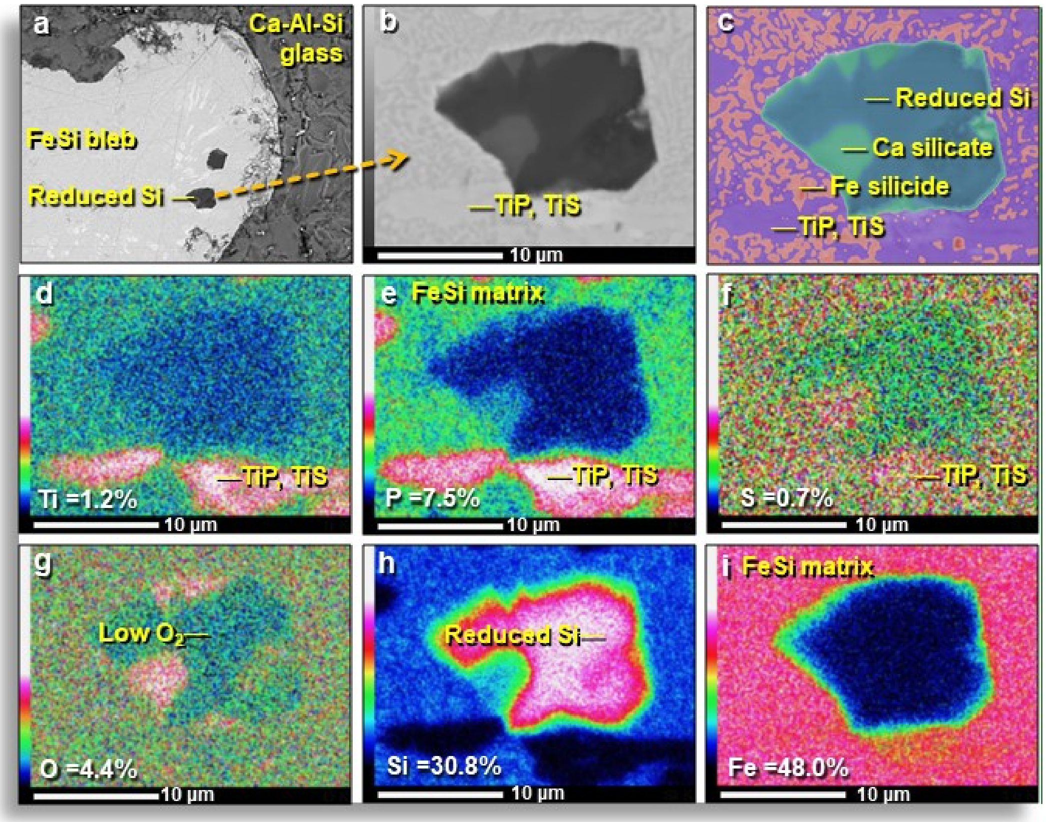

37

Figure S22. SEM images of titanium sulfide and titanium phosphide in furnace experiments. (a) Reduced-Si inclusion within FeSi globule on the surface of AH sediment laboratory-melted in Spark Sintering Furnace at 1850°C. **(b)** Close-up of inclusion at the yellow arrow. **(c)** Single-element (Ti) EDS map with intensity scale showing titanium phosphide (TiP) and titanium sulfide (TiS) (purple) in a matrix of Fe silicides (light red). **(d)-(i)** Single-element EDS map with intensity scale showing weight percentages of selected elements. Laboratory-melted bulk sediment from level 435, sample ES15, 395 cm depth.

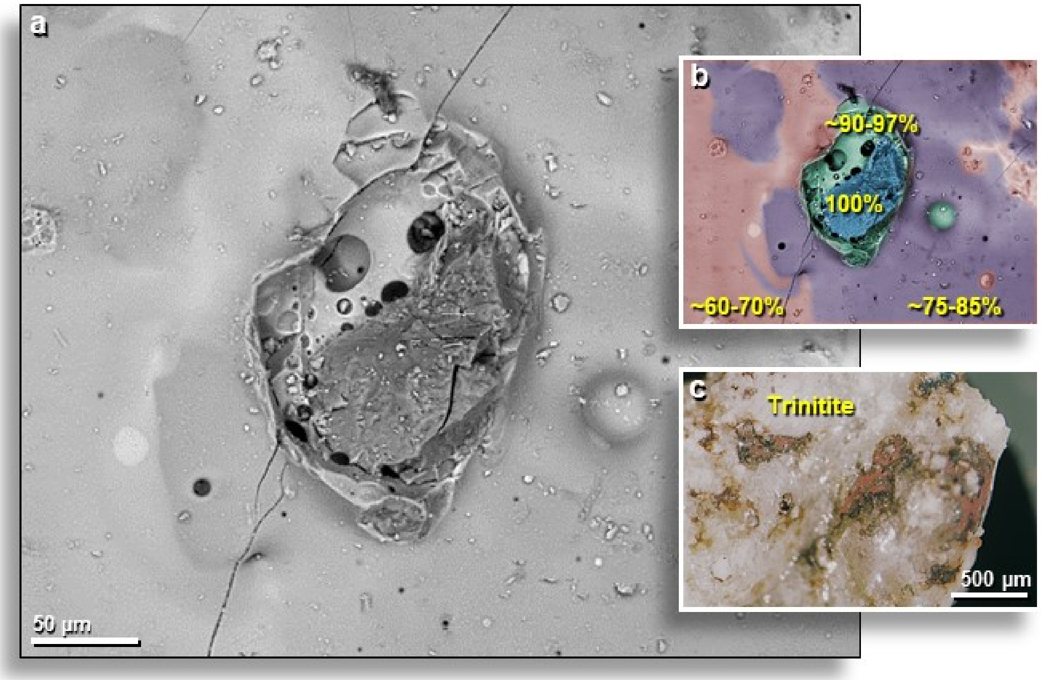

Figure S23. SEM images of melted quartz grain from the Trinity atomic bomb test. Another possible analog to the AH glass is meltglass produced during the 1945 Trinity nuclear airburst near Socorro, New Mexico at the Alamogordo Bombing Range, site of the world's first nuclear bomb test[1,8,9]. **(a)** Darker gray central portion of the image is an unmelted, shattered quartz grain. The area surrounding the grain represents nearly pure SiO_2 that possibly reached its boiling point of 2230°C, as indicated by high vesiculation. **(b)** Manually constructed EDS-based phase map with SiO_2 percentages shown for each color, ranging from ~60 wt.% to 100 wt.%. Aluminosilicate glass is in light red. **(c)** Photomicrograph of a fragment of trinitite; quartz-rich areas are white and Fe-rich areas are reddish-brown. Morphology of 135-µm-wide quartz grain on the outer surface of trinitite is similar to Abu Hureyra grains. Samples provided by co-author R.E.H.

The nuclear detonation occurred ~30 m above the ground with a TNT energy equivalent of ~21 kilotons, reaching an average plume temperature of 8000°C after three seconds[9]. The detonation formed a shallow crater 1.4 m deep and 80 m in diameter and melted the top 1–3 cm of the surface sediment, mostly composed of quartz, feldspar, muscovite, actinolite, and iron oxides. Molten material from the nuclear test, referred to as trinitite, fell back onto the surface up to distances of ~600 m, sometimes forming molten pools of glass. Some of the melted material was drawn into the rapidly rising plume and as the plume drifted north-eastward ~30 km from ground zero, trinitite rained out of the cloud as melted spherules and aerodynamically shaped glass. The Trinity ejecta includes irregularly shaped fragments, as well as melted teardrops, beads, and dumbbell shapes, many of which show collisional and accretional features. This evidence is morphologically similar to melted material recovered from Abu Hureyra.

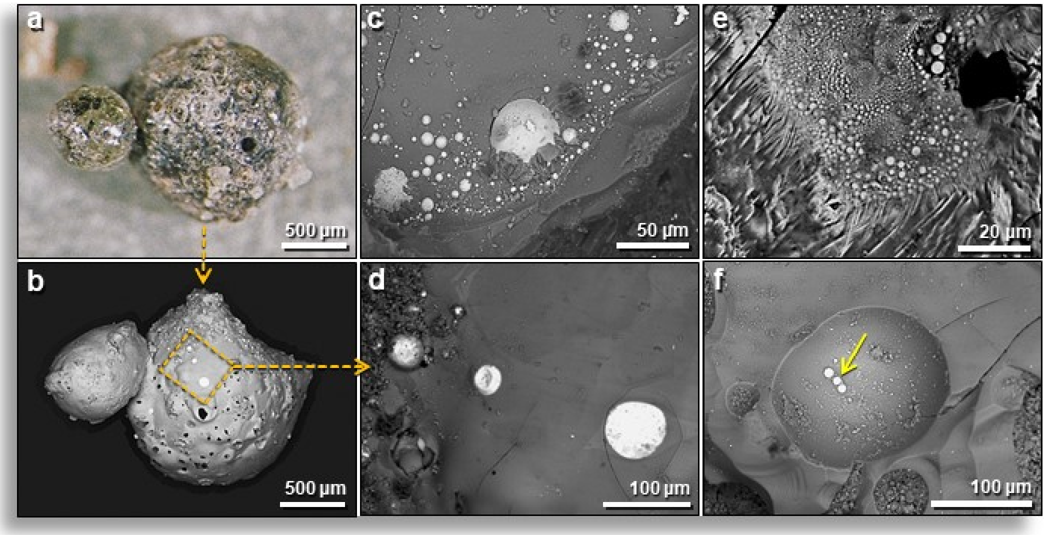

Figure S24. Fe-rich globules in glass from Trinity atomic test. (a) Photomicrograph of two fused glass spherules, one ~1400 µm wide, and the other ~700 µm across. **(b)** SEM image of the reverse side of glass spherules in Panel a. Box encloses three Fe-rich globules on spherule surfaces, as shown in Panel d. **(c)** Fe-rich globules on the outer surface of trinitite with the largest ~50 µm across. **(d)** Close-up of the boxed area on trinitite in Panel b; the largest globule is ~75 µm across. **(e)** Hundreds of Fe-rich globules on the inner wall of a trinitite vesicle. Also, note spindle-like crystals that formed inside the vesicle. **(f)** Globules inside 180-µm-wide vesicle exposed on the broken surface of trinitite. Fragment of trinitite was crushed to reveal vesicle. Note there are no globules on broken glass surface around vesicle, only inside the vesicle, suggesting they formed by vapor deposition. Samples provided by co-author R.E.H.

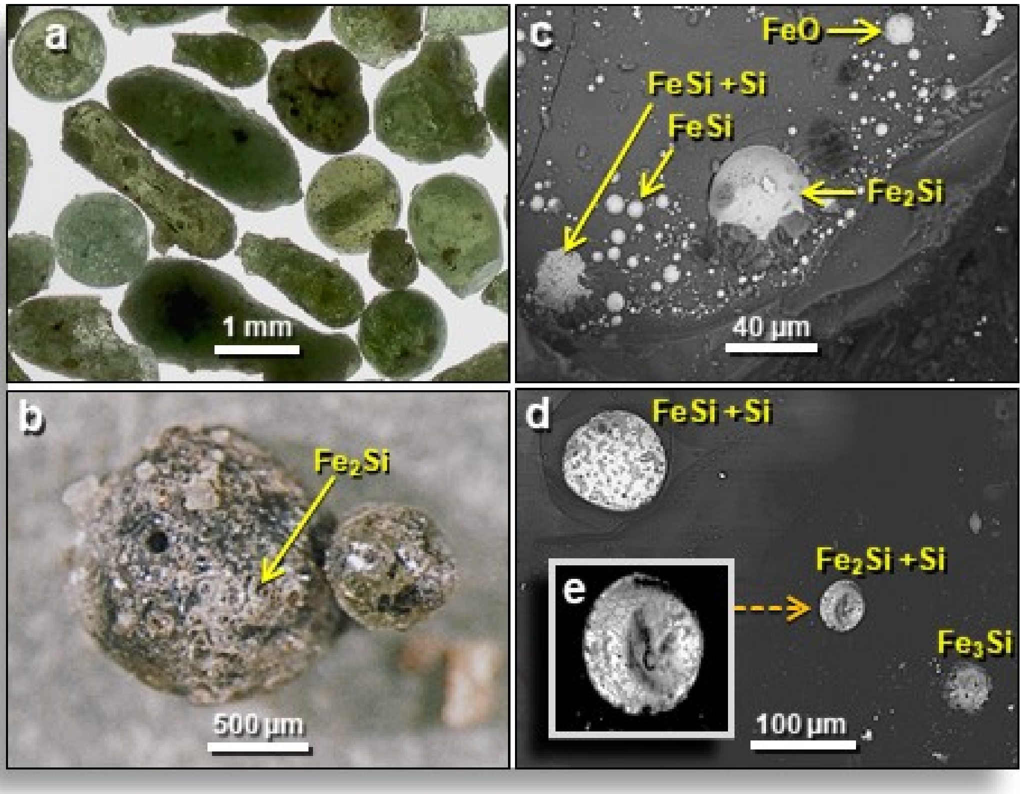

Figure S25. Iron silicide in trinitite from Trinity atomic testing. (a) Transmitted light photomicrograph of trinitite glass droplets; the longest ones are ~2.5 mm long. The green color is due to small amounts of Fe. Note dark inclusions that usually are partially- to fully-melted high-temperature minerals. **(b)** Photomicrograph of two fused spherules of trinitite; largest is ~1400 µm in diameter. Arrow points to a dark globule of iron silicide, Fe_2Si, that created a high-velocity impact crater with a raised rim. **(c)** SEM-EDS analyses indicate spherules and globules of three types of iron minerals: iron oxide (FeO) and two types of iron silicide (FeSi and Fe_2Si), coexisting with native Si. The largest globule is ~50 µm wide. From the inner wall of trinitite vesicle. **(d)** SEM-EDS analyses indicate globules of three types of iron silicide globules on the inner wall of trinitite vesicle. At top left is an immiscible mass of FeSi (bright) and native Si (darker); width is approximately 100 µm. The middle sphere is Fe_2Si with native Si, showing dimpled morphology, probably from the impact of partially congealed silicide into the plastic, molten host glass. The embedded sphere at right is Fe_3Si. **(e)** SEM close-up of the raised globule, showing concave surface caused by the high-velocity collision. Light-colored material = Fe_2Si, which is decorated around the platelet margins by dark bands of apparent native Si; irregular grains of an intermediate grayscale are likely a mixture of Fe_2Si and native Si. Samples provided by co-author R.E.H.

The ultra-high temperatures in the Trinity atomic bomb test caused thermal dissociation of the melt into the elemental species followed by condensation of the various phases. Because of fast-reaction kinetics, non-equilibrium conditions, and low fO_2, highly reduced compounds (metals and silicides) formed side-by-side with oxidized magnetite.

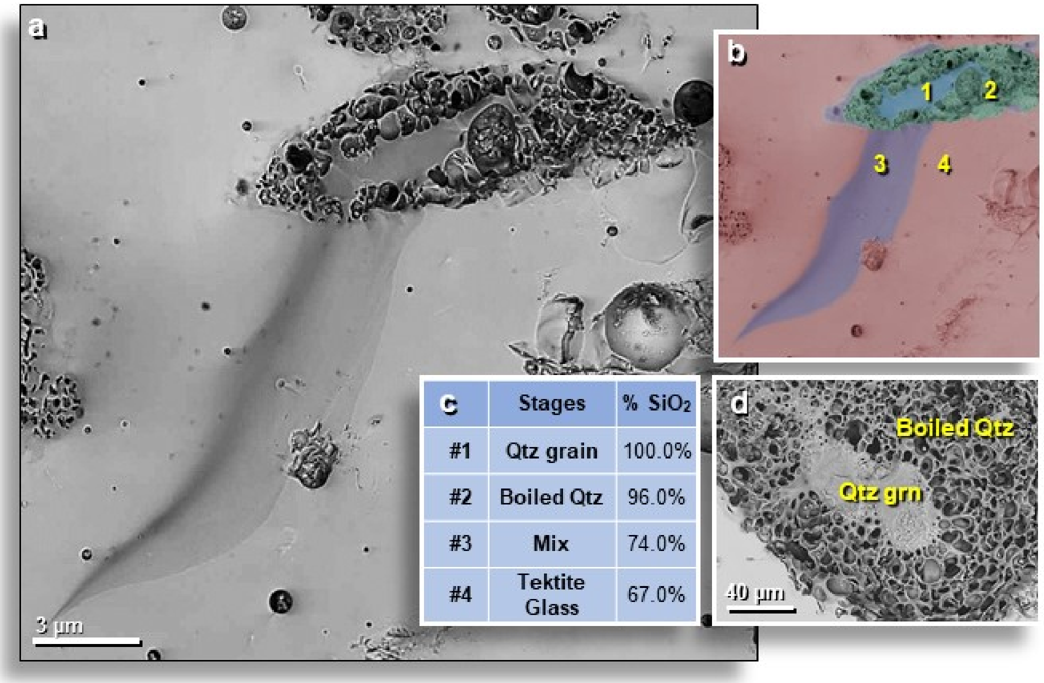

c	Stages	% SiO_2
#1	Qtz grain	100.0%
#2	Boiled Qtz	96.0%
#3	Mix	74.0%
#4	Tektite Glass	67.0%

Figure S26. SEM images of melted quartz grain from the Australasian tektite field. Partially melted 12-µm-long quartz grain on the outer surface of an aerodynamically-rounded tektite. **(a)** The darker gray upper central portion of the image is an unmelted, monocrystalline quartz grain. The surrounding highly vesicular area represents nearly pure SiO_2 that likely was flash-heated above its boiling point of 2230°C. **(b)** Manually constructed EDS-based phase map with blue representing unmelted grain and green and purple representing the diffusion of SiO_2 into the matrix. Aluminosilicate glass is in light red. **(c)** Table of data measured by SEM-EDS for Panel a,

showing percentages of SiO_2, ranging from 100 to 67.0 wt.%). **(d)** SEM image of another quartz grain from the same Australasian tektite.

Analyses of this grain suggest instantaneously melting and instantaneous quenching after flash heating that reached temperatures above the boiling point of quartz at 2230°C. The process was so rapid that it did not allow for the complete incorporation of the molten quartz grains into the bulk melt. This melted quartz grain is nearly identical to melted quartz grains in AH glass, suggesting that the latter could have resulted from a similar formation process.

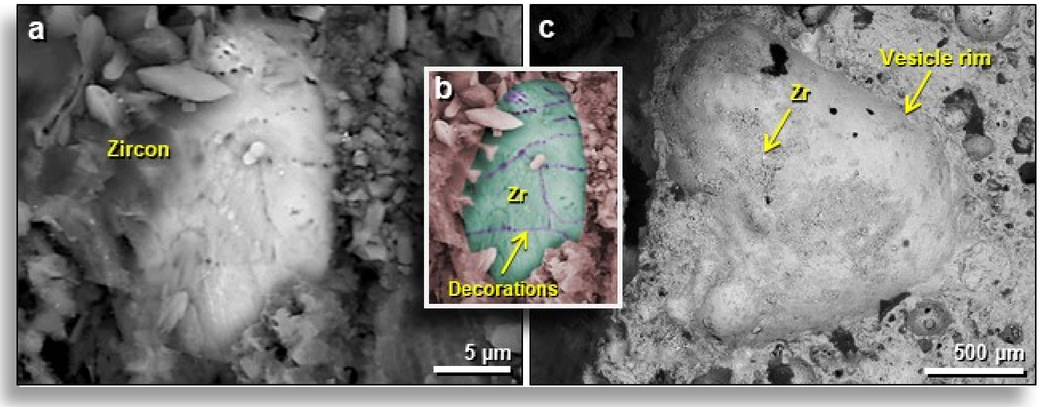

Figure S27. SEM images of zircon from cosmic airburst/impact at Dakhleh Oasis, Egypt. (a) Partially melted 22-µm-wide zircon grain on an outer surface of Dakhleh glass. Pointed crystals are melted aluminosilicates. **(b)** Manually constructed EDS-based phase map of grain. Zircon is green and the aluminosilicate matrix is light red. Arrow points to the purple line marking areas of thermal alteration that are "decorated" with sub-micron-sized vesicles, indicating that parts of the zircon began to vaporize at above its melting point of 1775°C. **(c)** ~1600-µm-wide vesicle in Dakhleh glass, with an arrow showing the location of the zircon grain. Samples from Dakhleh Oasis provided by Norm Lehrman, www.tektitesource.com.

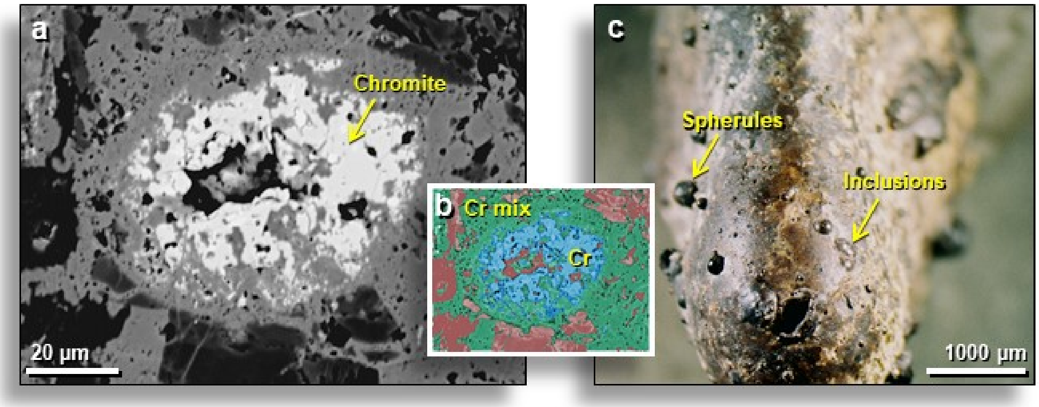

Figure S28. Melted chromite grain from Meteor Crater. Our investigations show that meltglass samples from Meteor Crater, Arizona also contain melted chromite grains. **(a)** SEM image of sectioned 95-µm-wide chromite grain from inside an impactite from Meteor Crater. **(b)** Manually constructed EDS-based phase map of same grain showing heavily altered chromite grain (blue) with diffusion (green) into the surrounding matrix (light red). High vesiculation suggests the grain reached or exceeded its boiling point of 2265°C. **(c)** A typical example of impact glass from Meteor Crater, showing embedded spherules and inclusions. Samples provided by co-author T.E.B.

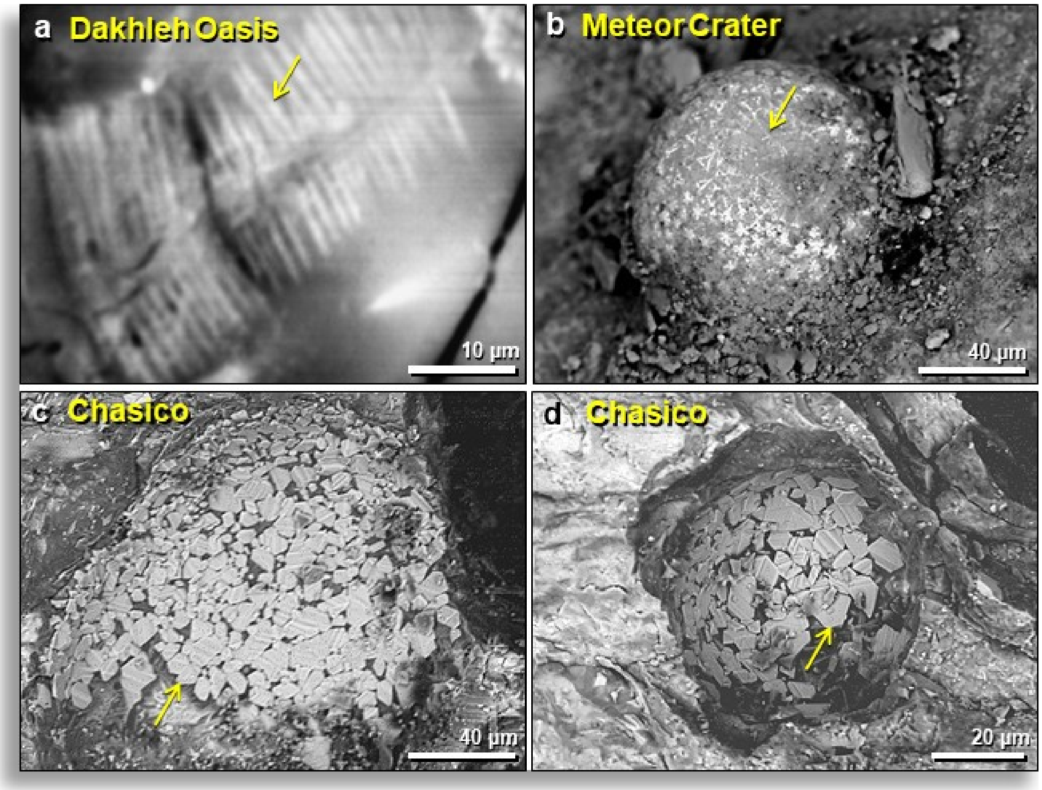

Figure S29. SEM images of Fe-rich textures in glasses from cosmic impact events. (a) Dendritic quench crystals inside vesicle of impact glass from Dakhleh Oasis, Egypt. **(b)** Dendritic quench crystals on top of ~100-µm-wide Fe-rich spherule embedded in impact glass from Meteor Crater. Other crystals display floret and trigonal shapes. **(c)** Impact meltglass with a 140-µm-wide vesicle from Chasico, Argentina. **(d)** 60-µm-wide vesicle lined with flat, polyhedral Fe crystals from Chasico, Argentina. Meteor Crater samples collected by co-author T.E.B. Impact glass samples provided by Norm Lehrman, www.tektitesource.com.

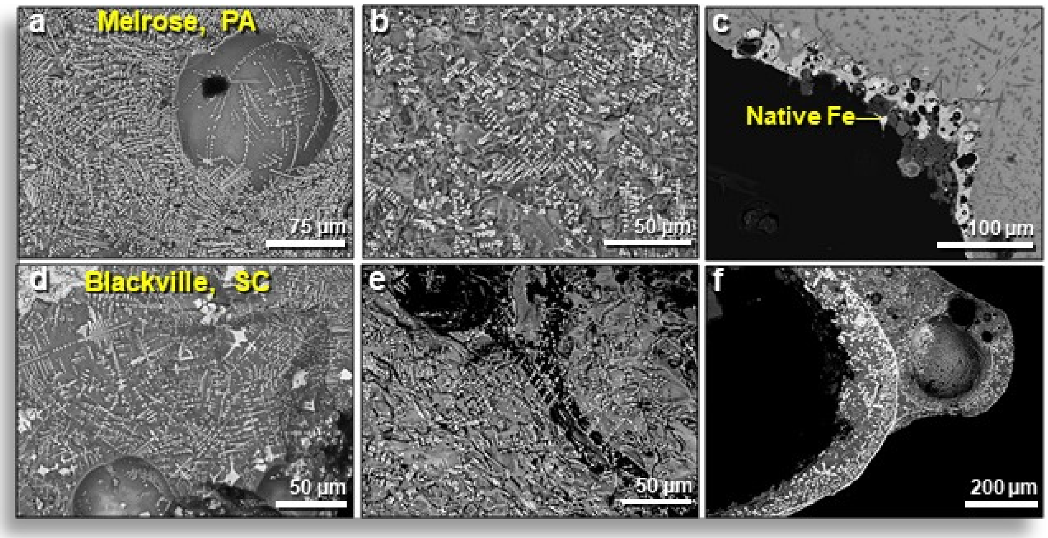

Figure S30. SEM images of Fe-rich textures inside sectioned YDB meltglass. (a)-(b) High-Z dendritic crystals in sectioned YDB meltglass from Melrose, Pennsylvania. In Panel a**,** note that crystals are present both in the matrix and inside the vesicle. **(c)** Sectioned vesicle with a label marking example of melted high-Z native iron globule (100 wt.% Fe^0). Melrose samples from Y1b

23-28 cm depth. **(d)-(e)** Dendritic crystals in YDB meltglass from Blackville, South Carolina. **(f)** Sectioned vesicles lined with high-Z, dendritic Fe crystals. Blackville glass from sample T13-240 cm depth.

Supporting Information: Text

Text S1. Previous Heating Experiments

Thy et al.[10] conducted heating experiments to estimate likely maximum temperatures for AH meltglass. Thermal analyses and modeling suggested maximum temperatures in the range of 1000-1200°C, rather than temperatures of >1700°C proposed by Bunch et al.[1]. Although this study found that ~1175°C is approximately the minimum temperature required to melt the local sediment, melting at minimum temperatures does not preclude reaching higher maximum temperatures. The presence of high-temperature, melted minerals, such as monazite, chromite, and chromferide, confirm that temperatures much higher than 1175°C under highly reducing conditions were reached during the melting of the glass. Thatched hut fires could have produced low-temperature glass at Abu Hureyra, but such fires could not have melted grains of quartz, monazite, chromite, chromferide, and suessite (1720° to 2300°C), as discussed in the main manuscript.

Text S2. Reflectance as a Temperature Indicator.

Reflectance Sample Analyses. See Methods below for preparation details. Reflectance was measured on fusinite (carbonized wood with clear cellular structure) in the case of charcoal particles, char (carbonized organic material of unknown origin with random degassing pores), and cell walls of vesicles (carbon spherule samples). In some cases, multiple reflectance populations were combined into a single reported measurement value (values commented as highly variable or multiple populations included). This was done where insufficient sample volume prevented a significant number of sample fragments from polishing into the examination surface, or where a dominant population was not clearly identified. Where sufficient numbers of fragments from a multi-population reflectance sample permitted the identification of the dominant population, that population was selected for the reported value. However, a second preparation of the same sample can result in a different dominant population occurring as the dominantly exposed fragments in the examination surface. Therefore, data from samples with multiple populations need to be interpreted cautiously.

General types of samples. Samples were divided into three types based on visual appearance: i) carbon spherules, ii) charcoal, and iii) char or amorphous carbon, as in sample ABU GLASS+CHAR, which does not fit into either category. See **Appendix, Fig. S4-S6.**

General type: Carbon spherules. These samples were subdivided into two categories: i) YDB carbon spherules, as extracted from sediment and ii) YDB carbon spherules that have been reheated to known temperatures. Carbon spherules generally have shapes that range from rounded to elongated to flattened ovoid. Sometimes, they are fragmented, consisting of a hardened, high relief rind surrounding a softer, lower relief interior. Generally, the carbon spherule rinds have higher reflectance than the carbon spherule interiors, reflecting lower inner temperatures. Extending from the rind inward are spherical to ovoid cells, usually filled with lower reflecting nonfluorescent material. Sometimes cells are empty or the lower reflecting cell-filling material contains an empty hole at its core. Proceeding further into the core of original carbon spherules, a collapsed spongy cellular structure is present, wherein individual open cells are poorly preserved.

Reheated carbon spherules are higher in reflectance than the original carbon spherules found in YDB sediment, and any retained cell filling material is of generally equivalent reflectance to cell walls. However, in general, most cells are open in reheated carbon spherule samples. The rinds of reheated carbon spherules contain more cell wall material than the interior of reheated spherules but rind reflectance and hardness are qualitatively identical to that of the interior material. Fe oxides occurring as discrete fragments also are present in some samples.

Carbon spherules are presumed to derive from most wildfires, including those associated with extraterrestrial impact events[11,12]. Alternately, they have been claimed to be modern fungal sclerotia and/or fecal pellets from insects[13,14]. Here, we discuss only the reflectance evidence; origin is not considered. The observations presented here are consistent with the prior descriptions given in the literature for carbon spherules, except that the occasional low-reflectance cell-filling material is reported here for the first time.

In an independent study, Scott et al.[13] and van Hoesel et al.[15] investigated YDB carbon spherules, such as those found at Abu Hureyra, which were inferred by Firestone et al.[11] to have formed at high temperatures during the impact event. Scott et al.[13] used charcoal reflectance, the same techniques used here[16]. Scott et al.[13] reported that all YDB carbon spherules show reflectance values indicating maximum temperatures of <450°C, thus precluding high-temperature impact fires. Similarly, van Hoesel et al.[15] reported charcoal reflectance for carbonaceous particles from the YDB layer in Europe. The results suggest maximum temperatures of ~420 ± 10°C, assuming a charring period of one hour, also precluding high-temperature impact fires. In contrast, Bunch et al.[1] reported charcoal and carbon spherules embedded in high-temperature melted glass from Abu Hureyra, Melrose (Pennsylvania), and Blackville (South Carolina), suggesting much higher temperatures for the carbon. These differences were explored by testing the hypothesis that, while reflectance is an adequate analytical technique for normal wildfires, it provides erroneous values for extremely brief, high-temperature events, such as a cosmic impact.

General type: Charcoal. These samples contain the maceral fusinite, which is the carbonized remains of charred wood with well-preserved cellular structure[17]. Charcoal samples are highly variable in reflectance, possibly indicating that incomplete or partial charring occurred in the same sample, or that heating was unevenly applied. Temperatures of combustion can be interpreted by a comparison of reflectance values to various experimentally derived chars[18-20]. However, given the high variability in reflectance present in the majority of samples evaluated herein, interpretations of combustion temperatures are preliminary and suspect. In addition, published calibrations of the reflectance of inertinite (a common organic component of coal and oil shale) to temperature are far from universally applied. Instead, they are restricted to isolated and specialized research projects and are apparently dependent on precursor material and charring time.

General type: Char. Char is high reflectance (1.3%) organic material with randomly-oriented, randomly-sized elongate ovoid to spherical degassing pores, as well as some dense regions lacking pores.

Sample: carbon in AH glass: ABU GLASS+CHAR contains 3 types of material: i) modern organic material, ii) char (as per Kwiecińska and Petersen[21]), and iii) meltglass). Some observed organic material is a low reflectance (0.2%) humic gel containing foraminifera and sponge spicules with dispersed mineral fragments. This is most likely post-depositional contamination. The char in sample ABU GLASS+CHAR appears to derive from the burning of gelified organic material, such as peat (e.g., Petersen[22]), possibly represented by the humic gel

fragments also present in this sample. The AH glass sample also contains layered Fe-oxides, which are also present as discrete fragments. Taken at face value, the uneven distribution of degassing porosity and dense areas suggests that the char formed at relatively low temperatures. However, its association with high-temperature meltglass argues for a more complex heating history.

Sample: modern reeds, oak, and pine. This experiment was designed to replicate impact conditions, where a high-temperature combustion source is applied unidirectionally at a short duration. The combusted portion of the reed sample was converted to 96.3 wt.% gases, 0.9 wt.% ash, 0.4 wt.% charcoal, and 2.4 wt.% glassy spherules[23]. For fragments of oak and pine, ~97 wt.% was transformed into gases, <1 wt.% into charcoal and ash, and ~2 wt.% into spherules[23]. Ash and glassy spherules were produced from where the wood was exposed to the highest heat, grading into charred wood, and then into unburned wood farthest from the heat source. Charcoal samples were collected from the end of the section that remained after combustion. Very little of the material exposed to the flame survived (<4 wt.%), and even less survived as charcoal (<1 wt.%). Samples of reed (*Phragmites australis*), oak (*Quercus turbinella*), and pine (*Pinus ponderosa*) were heated using an oxygen/propylene torch. Portions of short sections of each material (~6 × 0.5 cm) were exposed to a direct flame for ~30 seconds at temperatures of ~1700 to ~2600°C, the highest temperature measurable by a thermocouple.

The Reed Char sample contains some modern un-charred wood (fluorescent) fragments as well as fragments that exhibit a transition from semifusinite to fusinite (less to more carbonized woody material in the same fragment, i.e., less reflective to more reflective). Reflectance measurements did not confirm the measured high temperatures of >1700°C for the reed, oak, or pine. Instead, $\%R_o$ values indicated average temperatures of ~632°, 631°, and 648°C, respectively, far below the measured temperatures (**Appendix, Table S5**).

Sample: trinitite. Reflectance was measured for a charred twig partially covered with trinitite, recovered from the blast zone of the Trinity atomic bomb test at Alamogordo Bombing Range, NM in 1945. The average plume temperature was ~8,000°C at 3 seconds[9], falling after ~3 seconds to ~1720°C[8], the melting point of quartz. In addition to fusinite, the Trinitite Lump Char sample contains some relatively low reflecting semifusinite with preserved cellular structure. These semifusinite fragments in Trinitite Lump Char contain inclusions of fluorescing liptinite coal macerals (possible remains of corky material) as well as areas of mesophase. In this sample, the presence of fluorescing liptinite material preserved in structured semifusinite immediately adjacent to what is interpreted as mesophase indicates a transient heating event applied to a modern wood sample. Preserved cell structure indicates no humification occurred prior to the carbonization event and the presence of fluorescing material indicates transient heat as well as low levels of post-charring oxidation. The mesophase areas indicate molecular order precipitating from a plastic phase (boiling fluids and remnant solids). Reflectance measurements did not yield the correct temperature of the trinitite (minimum temperature: 1250°C), but rather, average $\%R_o$ values yield a temperature of ~406°C (**Appendix, Table S5**).

Sample: activated carbon and charcoal feedstock. Reflectance was measured for samples of commercially-made activated carbon and samples of the charred feedstock used for making activated carbon, provided by Calgon Carbon Corporation. According to the company, the raw material (coconut shells) had been subjected to temperatures of ~450°C for ~8 hours under conditions that restricted but did not eliminate oxygen, similar to ambient atmospheres during a cosmic impact event. This carbon feedstock is then used to produce activated carbon by processing it with steam for ~8 hours at ≥1100°C to create anoxic conditions, as in a cosmic impact event). Reflectance-derived inferred temperatures closely matched the maximum temperature of the

charred coconut (511°C; **Appendix, Table S5**). On the other hand, reflectance values for the activated carbon incorrectly indicated an average temperature of ~585°C instead of the recorded temperature of ≥1100°C (**Appendix, Table S5**).

Sample: YDB carbon spherules. We investigated whether reflectance measurements can determine the accurate temperatures for nanodiamond-rich carbon spherules that had been reheated to known temperatures measured with a thermocouple. Nanodiamond-rich carbon spherules from the YDB layer in Gainey, Michigan; Kimbel Bay, North Carolina; and Indian Creek, Montana were placed separately in a tube furnace filled with a CO_2 atmosphere, ramped up for ~10 minutes to the maximum temperature of 650-850°C, and held there for ~5 min. The average $\%R_o$ for Gainey indicated 537°C, compared to a measured temperature of 730°C; the average $\%R_o$ for Kimbel Bay indicated 515°C, compared to a measured temperature of 700°C; and the average for Indian Creek was 567°C, compared to a measured temperature of 650°C (**Appendix, Table S5**). Thus, reflectance values are relatively close to but lower than measured temperatures for all three YDB carbon samples.

In similar reflectance experiments, Scott et al.[13] investigated carbon spherules from several YDB sites and found reflectance values of <2%R_o, consistent with charring temperatures of <450°C. Similarly, van Hoesel et al.[15] reported that charcoal particles from the YDB-age Usselo horizon show a reflectance of 0.96 ± 0.06% R_o, indicating a charring temperature of approximately 420 ± 10°C, assuming a charring period of 1 h. However, those workers reached their conclusions based on experimental charring of organic material for a duration of one hour or more. Guo and Bustin[24] found that the duration of heating for charcoal must be considered when inferring fire temperatures. The experimental conditions in those studies are much different than those in cosmic impacts, and so those results are inapplicable to high-temperature events of extremely short duration, ranging from a few seconds to a few minutes, as detailed in Schultz et al.[25]

Sample: Tunguska charcoal. To compare a known impact fire to the wildfire charcoal at Abu Hureyra, we measured reflectance on charcoal from the Tunguska airburst, which felled 80 million trees[26] and triggered wildfires across 2150 km². The charcoal samples were extracted from the peaty impact layer that dates to 1908, the time of the airburst. The average reflectance-derived temperature for the Tunguska charcoal is 413°C. The original formation temperatures for the charcoal are unclear, and so, temperatures can only be inferred based on other lines of evidence. Nanodiamond-rich carbon found in nearby peat samples was estimated to have been exposed to temperatures from 700 to 1475°C, and the charcoal-rich layer contained Fe-rich spherules that melted at ~1500°C[23]. Thus, the charcoal from Tunguska most likely formed at ≥1500°C (**Appendix, Table S5**). Reflectance values suggest temperatures for Tunguska charcoal that are much lower than actual temperatures.

The effect of duration on inferred temperatures. Guo and Bustin[24] found that the duration of heating has a major effect on charcoal reflectance, and so, a key question is whether charring for one hour is comparable to charring for a few seconds to a few minutes. In one experiment with charring wood, Guo and Bustin[24] found that after exposure to 600°C for 40 minutes, reflectance values were ~8.5× higher than for 6 minutes. In other words, the lower value at 6 minutes erroneously indicated a heating temperature of ~350°C, instead of 600°C. Their results conclusively demonstrated that the duration of heating for charcoal must be considered when inferring fire temperatures.

Text S3. Possible Vapor Deposition.

Epitaxial films may grow from gaseous or liquid precursors in a process that deposits a crystalline layer over a crystalline substrate, where there is structural continuity between the overlayer and the substrate. However, here the process is only analogous and not epitaxial in the strictest sense because epitaxia entails growth of crystals on a crystal substrate, not deposition of amorphous glass on glass. Lechatelierite cannot be produced volcanically but is found in fulgurites, the tubular meltglass formed during a lightning strike[1,27]. No piece of AH glass investigated resembles the distinctive tubular shape of a fulgurite. Lechatelierite is also common during cosmic impact events, such as at Meteor Crater, AZ[28], Haughton Crater, Canada[29], Australasian tektites[30], Libyan Desert glass[30], and Dakhleh glass[31].

Text S4. Oxygen fugacity (oxygen deficiency).

Low oxygen fugacity (fO_2) occurs within an airburst/impact fireball[32]. Native Fe spherules within trinitite glass indicate that regions of extremely low fO_2 (within the metal) coexisted with high fO_2 (within the glass matrix) over very short distances. Native Fe is common in extraterrestrial material but extremely rare in terrestrial rocks, mostly limited to trinitite and to rocks in contact with underground coal fires[1], which are not known to have occurred near Abu Hureyra. In the absence of sufficient oxygen, Fe sometimes combined with silica (yielding silicides, e.g., Fe_3Si), sulfur (yielding sulfides, e.g., FeS), carbon (yielding carbides, Fe_3C), or phosphorus (yielding phosphides, Fe_3P). Several variants of these minerals are known only from meteorites, and others were discovered in meteorites first, and only later found at a terrestrial location Such low-fO_2 minerals have been documented in YDB meltglass from Blackville, South Carolina, and Melrose, Pennsylvania[1], in trinitite[1], and now, in meltglass from Abu Hureyra.

One question is how these oxygen-reduced, high fO_2 Fe and Ni phases formed in the highly restricted space inside AH glass vesicles. Carbon concentrations in some fragments of AH glass are estimated to have reached 15 to 25 wt.%, based on EDS spectral peak heights, and therefore, it is proposed that the high carbon content in the high-SiO_2 glass yielded highly reducing local conditions that promoted the formation of native Fe and NiFe, rather than magnetite. AH glass and spherules probably resulted from nearly instantaneous melting of both carbonate-rich sediment and significant amounts of carbon-rich biomass and the vesicles in AH glass trapped carbon in both solid and gaseous states, with carbon acting as a reducing agent. Oxygen fugacities were highly variable, some vesicles, contain coexisting oxidized Ni-bearing magnetite and native Fe and NiFe metals within sub-millimeter distances of each other.

The likely formation mechanism for awaruite is different. These grains occur on the outside of spherules and AH glass, not within vesicles, and thus, were not produced in a closed system. In this case, the NiFe-bearing spherule melts were also subjected to changing atmospheric conditions and low fO_2 in the hypervelocity impact cloud. The morphologies and compositions of some AH glass fragments imply that they formed in the impact cloud similar to the way that trinitite formed in the ground-hugging base surge produced by atomic bomb tests. Other AH glass fragments resemble trinitite droplets that were drawn up into the rising atomic bomb plume and then fell out as melt droplets. The spiral morphology of some AH glass droplets is very similar to some trinitite droplets that show evidence of cooling while rapidly rotating in the impact cloud.

Some of the Ni-rich material may contain a small percentage (<1%) of Ni from the impactor. Pechersky et al.[33], who analyzed samples from 25 different types of meteorites, found that metallic inclusions in meteorites clustered in three different groups: i) pure or native iron, ii) kamacite containing 3–6% Ni, and iii) taenite containing ~50% Ni. AH glass displays the same

three groups, in which Fe and Ni concentrations overlap or are close to concentrations in meteorites, suggesting that the Ni in AH glass may derive from meteoritic material, most likely during an airburst, possibly by a small dust-rich comet or a rubble-pile asteroid[34].

In summary, the temperatures involved in forming AH glass are important, but more important is the rapid, non-equilibrium cooling under highly variable fO_2. Such fast-reaction, isentropic conditions (i.e., having equal entropy) are known to exist only in lunar materials, fulgurites, aerial nuclear detonations, and cosmic impact events. Only the latter is a plausible source for AH glass.

Text S5. Transmission FTIR and water content.

Other results. A layered sample from the Australasian tektite field (Muong Nong, Laos) showed H_2O content ranging from 182-227 ppm (n=6), which falls within the published FTIR range of 90-300 ppm[35]. Another example, a splash-form Australasian tektite, had a low H_2O content ranging from 78-89 ppm (n=7), falling within with the published range of 40-120 ppm for various splash-form tektites[35]. Both tektite glass samples analyzed yielded results within the range of water content in other tektites from various fields (~20 to 500 ppm)[35].

A sample of known cosmic impact glass from the Darwin crater in Western Tasmania, Australia had an H_2O concentration of 611 ppm (n=7), similar to the previously published value of 470 ppm (n=2)[36]. One sample of impact glass from the Zhamanshin crater in western Kazakhstan yielded a range of 705-1036 ppm (n=10), which is higher than the reported range of 50-630 ppm for the crater[35] but overlaps published values for H_2O in various impact glasses (80-1300 ppm). It also overlaps previously reported Zhamanshin glass values of ≥1000 ppm[37,38].

FTIR analysis of a sample of trinitite from the Trinity airburst at the Alamogordo Bombing Range, New Mexico yielded water contents of 283-510 ppm, somewhat higher than previous reports of 70-100 ppm[39].

Lightning-produced glass (fulgurites) from near Socorro, New Mexico yielded an H_2O concentration of 159 ppm (n=7), which is lower than some published values of 500-1400 ppm for some fulgurites[37,40].

Chaiten volcanic glass samples (rhyolite) from Chile had an H_2O content of 1497 to 1769 ppm, consistent with previous values of 1200 ppm reported for this volcano[41,42]. These values are in the range of H_2O contents (500-4000 ppm) reported for various other volcanic glasses[43-47], excluding volcanic glass samples that appear to have absorbed additional water after cooling.

Biomass glass, a natural glassy slag from Botswana and other sites in Africa, was not analyzed but has been reported to have an H_2O concentration of 1000 to 9100 ppm[48,49]. Medieval anthropogenic glass from an archaeological site has been reported to contain from 500 ppm to 7,600 ppm H_2O[50]. Other examples of modern human-produced glasses range from ~1000 ppm to 12 wt.% H_2O[51,52].

Text S6. Remanent Magnetism.

Results for other YDB sites. One sample from Blackville, South Carolina displayed magnetization along one vector that was moderately higher than the normal geomagnetic field. This result is inconsistent with normal terrestrial rocks but consistent with impact-related material, possibly due to thermal and mechanical shock, as seen in laboratory remanent magnetism experiments[53]. The Melrose samples fall into 2 groups; the first with 3 samples showed low levels of initial magnetization, meaning that this group could not have formed by lightning. This group had soft magnetic carriers and showed dramatic, continuous changes of the magnetization vectors,

consistent with rotation of the meltglass during cooling, as could have occurred during either a surface impact or a cosmic airburst that lofted molten material into the air. The second group of 2 samples showed maximum levels of magnetization, consistent with having been melted either by natural lightning strikes or by impact-induced lightning[54], which occurs during both volcanic eruptions and cosmic impacts. Thus, two of the Melrose samples of meltglass could have formed by lightning, but three other Melrose samples could not have formed that way.

Text S7. Discussion of potential meltglass formation mechanisms

Building fires. Thy et al.[10] proposed that thatched hut fires created AH glass at temperatures in the range of 1100-1200°C. However, based on our laboratory heating experience, we conclude that the presence of melted chromite and monazite in AH glass indicates flux-adjusted temperatures of ~1872-2065°C, well above the highest known temperatures for building fires. For comparison, the phosphorus-induced firebombing of Dresden, Germany, during World War II produced updrafts of ~275 km/h, forming fire tornados that produced maximum temperatures of 1000°C[55], sufficient to soften but not melt glass and too low to melt iron structural materials. It seems unlikely that small thatched huts could burn at temperatures as high as modern buildings, and therefore, the AH huts are unlikely to have reached temperatures of ~1200°C.

Thy et al.[10] also investigated meltglass fragments at other excavated archaeological sites in northern Syria, including Mureybet, ~32 km north of Abu Hureyra, and Jerf el Ahmar, ~76 km north, from which the lead author kindly provided samples for this study. Our analyses by light microscopy show that they are morphologically identical to those from Abu Hureyra, displaying both plant imprints and aerodynamic shaping. However, our SEM-EDS analyses of only a few available glass samples identified no high-temperature melted minerals in the samples, precluding a direct comparison with AH glass. Thy et al.[10] did not report sedimentary concentrations of meltglass, so it is unknown whether or not the meltglass is rare or common at those sites. Thus, those investigators may be correct that meltglass from these other sites formed during hut fires, unlike AH glass, and therefore, lacks high-temperature melted minerals.

The sediments investigated by Thy et al.[10] span 1500 years, a wide range that led them to conclude that the presence of meltglass at the sites in northern Syria is inconsistent with a single cosmic impact. However, Bunch et al.[1] and this study found very small amounts of AH glass in Holocene sediments above the YDB layer and attribute this “young” meltglass not to additional impacts but rather to the reworking (redeposition) of deeper YDB-age sediment. In support of that, Moore et al.[5] documented extensive reworking of 12,800-year-old meltglass as the most likely explanation for apparently younger meltglass.

One interesting possibility deserves future study. The question is whether the meltglass at Mureybet and Jerf el Ahmar was created at the time of the YDB impact event and then was later reworked into younger sediment. If so, YDB-age meltglass covers a much wider regional area.

Biomass or “haystack” fires. Thy et al.[48] reported that biomass glass or slag is sometimes found in midden piles of prehistoric settlements in Africa with estimated formation temperatures of 1155-1290°C, well below the melting points of high-temperature minerals in age glass. The lead author kindly provided us with samples of biomass glass, which we found to be morphologically different from AH glass and to contain no high-temperature minerals, making it easily distinguishable.

The lead author of Thy et al.[10] provided samples of biomass slag from Africa that are morphologically somewhat similar to AH glass but typically rougher and more heterogeneous. Analyses of the slag using SEM-EDS found that its composition is somewhat different from AH

glass; the main component is SiO_2 at 66.9 wt.% for biomass slag compared to 50.9 wt.% for Abu Hureyra. Investigations of the outer surfaces of biomass slag samples and interior material on sectioned slides revealed no melted, high-temperature mineral grains like those on AH glass. Instead, there are only low-temperature melted grains, including plagioclase and feldspar with melting points of ~1200°C, consistent with a temperature range of 1155-1290°C, as estimated for biomass glass in Thy et al.[48]. The biomass glass investigated formed at much lower temperatures than AH glass. In addition, biomass glass typically cools slowly, so that the pieces of glass we analyzed show no flow marks that are evident in AH glass.

Anthropogenic contamination. AH glass production by Abu Hureyra villagers can be ruled out because they were unable to achieve the requisite temperatures. Pottery-making began ~14,000 years ago, but maximum temperatures were <1050°C[48]; copper smelting began ~7000 years ago but only reached temperatures of ~1100°C[1]; glass-making at ~5000 years ago only reached ~1100°C[1]. These temperatures are too low to have melted quartz, chromite, and/or zircon grains. In addition, contamination from modern human activities can be eliminated because AH glass was buried nearly 4 m below the modern surface and sealed beneath undisturbed living floors.

Coal-seam fires. Subsurface burning of coal seams can produce a glasslike material, called clinker, or scoria[1], at low-pressure formation temperatures of 1000-1400°C. However, these temperatures are too low to melt zircon or chromite, as observed at Abu Hureyra. In addition, no coal deposits are known near Abu Hureyra; the nearest are in Turkey.

Lightning-induced melting. Temperatures in fulgurites can far exceed 1720°C, hot enough to fully melt quartz, making lightning a potential source of AH glass. However, magnetic measurements, as discussed in the section below, indicate that lightning did not produce AH glass, which lacks the high remanent magnetism characteristic of fulgurites. In addition, meltglass containing high-temperature minerals are found concentrated at Abu Hureyra in 12,800-year-old sediment across Trenches D, E, and G[5], while almost none are found above and below. This evidence is widely distributed. Trench E is ~122 m from Trench D and ~110 m from Trench G; Trenches D and G are ~175 m apart, an area of about 6700 m^2, or ~0.67 hectares.

Text S8. Astronomical Environment

Astronomical discoveries over the last few decades demonstrate that the mass distribution of comets is biased towards larger bodies, up to 300 km in diameter. A single giant comet (diameter ≳100 km) may contain >100 times the mass of all the asteroids that currently threaten Earth, and if thrown into a short-period, Earth-crossing orbit, it would disintegrate, like most comets do[56]. The hierarchic fragmentation of a large comet in a short-period orbit may yield many hundreds of short-lived debris streams comprising dust, boulders, and cometary fragments, expanding along the orbital track. Over the lifetime of the comet, such co-moving fragments constitute a significant terrestrial hazard[57,58].

Such large comets drift into the near-Earth environment frequently in relatively short geological timescales. In fact, the fragmented remains of two such bodies are present in the inner Solar System today. One of them, the Taurid Complex, is composed of debris from an ~100-km-wide comet that arrived at least 20,000 to 30,000 years ago from the Centaur system of large comets, after which it disintegrated hierarchically into short-period, Earth-crossing orbits[59,60]. One or two intersections with such material, sufficiently massive to yield YDB-like catastrophes, are reasonably probable over the course of the 20,000-year-long breakup of this Taurid progenitor, thus providing a plausible impact mechanism for the YDB Impact Hypothesis.

METHODS

SEM-EDS analyses used a JEOL JSM 6010PLUS/LA at Elizabeth City State University and a Hitachi S3200N variable pressure scanning electron microscope (VPSEM) at North Carolina State University. All SEM imagery was acquired at a resolution of 2560 × 1920 pixels. Images were uniformly post-processed for contrast and brightness, if necessary, using Adobe Photoshop CC2014. SEM-EDS images for all analytical tests were uniformly post-processed for contrast and brightness, if necessary, using Adobe Photoshop CC2014. Colorized phase maps were manually constructed with Adobe Photoshop CC2014, based on multiple single-element EDS maps with rainbow-colored intensity scales. M.A.L. and A.V.A. performed the analyses.

Reflectance. Samples were received into the laboratory in glass vials. Eight of seventeen samples were prepared in 1-inch thermoplastic mounts heated at 360 °F and 4000 psi pressure for 10 minutes, with no additional processing. The examination surfaces were ground and polished following ASTM D2797 (ASTM 2012a). Nine low-volume samples were poured into pre-drilled holes in otherwise identical 1-inch molds, mixed with thermoplastic binder powder and mounted at the conditions given above. Examination surfaces of the low volume samples were slabbed with a wafer saw prior to grinding and polishing via ASTM D2797. All samples were desiccated overnight prior to reflectance measurements.

Select sample mounts were mapped using a Leica DM4000 microscope equipped with customized LED illumination and image mosaic software from Hilgers, Inc. A Leica DMRX Pol microscope equipped with a J&M photomultiplier (PMT) and Zeiss MRc digital camera was used for reflectance analysis and imaging. Reflectance was determined according to ASTM D7708[61]. Reflectance values were checked on the Hilgers system, which employs a camera as the detector. Reflectance measurements were calibrated using a K&B cubic zirconia standard (3.13%; used with PMT system) and a K&B glass standard (1.31%; used with a camera system).

Samples were examined dry at 50x with white LED illumination for mapping and at 500x under oil immersion with tungsten halogen incident light (PMT system) for reflectance and with white and blue LED (camera system). Sample and material identifications are noted in the photographs. P.H. performed all analyses and interpreted the results.

Transmission FTIR and water content. The glasses were prepared as doubly polished wafers for analysis with a Nicolet iN10 MX infrared imaging microscope with an attached liquid-N_2-cooled MCT-B detector at the USGS in Menlo Park, CA USA. The iN10 MX is purged with low-CO_2, dry air and is equipped with a collar that, for transmission experiments, can be lowered around the sample stage to maximize purge during sample and reference collection[41]. A reference spectrum (R) was collected away from the hydrous glass sample through the BaF_2 window, transparent to both visible and infrared light. The sample surface was brought into focus with reflected light prior to the collection of the sample spectrum (S). Sufficient scans were collected to minimize noise (usually 256) at 4 cm^{-1} resolution. A square aperture was used to define the precise area of analysis, which could be sized as small as 20μm to avoid bubbles. Measured absorbance (a) was calculated as follows.

$$A = \log (I_R/I_S) \qquad \text{[Eq. 1]}$$

where I_R = the radiation transmitted in the reference spectrum and I_S = radiation transmitted through the sample plus reference.

We analyzed the 3570 cm^{-1} peak, which measures total water (OH- and molecular water; i.e., H_2O_m)[41]. The samples had very low H_2O, and in such cases for silicate glass, the water is expected to be found entirely as OH-. The peak height for the principle OH^- stretching band

(located near 3600 cm^{-1}) was measured from a flat baseline to the maximum peak height. Concentrations were calculated assuming Beer's Law:

$$\text{wt.\% } H_2O = 1802A/(\varepsilon d\rho) \qquad \text{[Eq. 2]}$$

where A is the absorption for the peak of interest, ε is the extinction coefficient for the peak in L mol-cm^{-1}, ρ is the density in g L^{-1}, and d is the thickness of the doubly polished sample in cm. An ε value of 75 was chosen as reasonable for high-silica tektite glasses[35], though H_2O is quantified with ε ranging from 63 (basaltic glass, Dixon et al., 1988) to 150 (cf. Paterson[62]) to provide a maximum possible range of H_2O contents within the sample suite). All glass densities were assumed to be 2350 g cm^{-3} (anhydrous rhyolite), though a few of the more mafic samples may have had densities 10-15% greater. An increase in the assumed density would result in a proportional decrease in the final calculated H_2O concentration. Wafer thicknesses were measured with a digital pin micrometer. Jake Lowenstern, USGS, Menlo Park, CA performed all analyses and interpreted the results.

Electron microprobe analyses were conducted using a JEOL 8900 at the U.S. Geological Survey in Menlo Park, California (USA). Concentrations in samples and standards are reported as oxides, except for Cl. Each spot was analyzed two times; once for major elements (Si, Al, Ca, K, Na, Mg, and Fe) and once for minor elements (F, Cl, Mg, P, Mn, S, Ti). The first analysis used a 10-nA, 2-μm diameter beam, and count times of 20 seconds for all elements except for 10 seconds on easily volatilized Na. Standards included a variety of glasses (VG2, RLS-132), minerals (Tiburon albite, OR1, sodalite, barite,) and simple elemental oxides (TiO_2, Mn_2O_3) in standard use at the Menlo Park facility[47]. Jake Lowenstern, USGS, Menlo Park, CA performed all analyses and interpreted the results.

SUPPORTING INFORMATION: Tables

Table S1. Fourteen Abu Hureyra radiocarbon dates from Moore et al.[5] were used to develop a Bayesian age-depth model by Kennett et al.[4]. The most abundant YDB Abu Hureyra meltglass was found in level 445 (green), directly dated to 12,933 ± 68 cal BP (UCIAMS-105429, 11,070 ± 40 ^{14}C BP) with smaller amounts of glass in the other green-highlighted levels with a Bayesian-modeled calibrated radiocarbon age of 12,825 ± 55 cal BP at 68% Confidence Interval (roughly equivalent to one sigma standard deviation), overlapping the previously published YDB age of 12,835 to 12735 cal BP for ~40 sites[4]. The green-highlighted age overlaps the published YDB age range of 12,835 to 12,735[4], indicating that identification of the YDB layer is robust. Anomalously old and young dates were excluded by Bayesian analysis. Dates based on humic fractions of bones were considered unreliable and were excluded[5]. "Extracted" column lists field numbers of samples. "Elevations" for each level (meters above sea level) vary across the trench and are averaged. "OxA" = Oxford Radiocarbon Accelerator Unit. "BM" = British Museum Radiocarbon Laboratory. "UCIAMS" = University of California, Irvine. W. M. Keck Carbon Cycle. Accelerator Mass Spectrometry Laboratory. Dates from Moore et al.[5] and Bunch et al.[1], as noted.

Level	Phases	Extracted	Elevation (m.a.s.l.)	Depth (cm)	14C Date	±	IntCal13	±	Lab #	Material dated	References
**Accepted dates: Phases 1-2, chronologically within phase**											
425	2		285.05	380	10000	170	**11595**	**285**	OxA-473	Charred sheep bone	Moore, 2000
419	3		285.05	372	10050	180	**11669**	**305**	OxA-407	Charred wild sheep bone	Moore, 2000
430	2		284.90	384	10420	140	**12264**	**225**	OxA-397	Grain fragments of wild einkorn	Moore, 2000
430	2		284.90	384	10490	150	**12342**	**218**	OxA-434	Charred gazelle bone	Moore, 2000
457	2	E313	284.60	413	10600	200	**12426**	**262**	OxA-171	Grain fragments of wild einkorn	Moore, 2000
54	2	E54	284.70	340	10650	50	**12625**	**47**	OxA-12111	Charcoal	Moore, 2000
445	2	E301	284.50	405	--	--	**12825**	**55**	--	Bayesian-calculated age of YDB	This paper
445	2	E301	284.50	405	11070	40	**12933**	**68**	UCIAMS-105429	Charcoal	Bunch, 2012
447	2		284.45	410	11140	140	**12991**	**138**	BM-1718R	Charcoal	Moore, 2000
460	2		284.55	411	11020	150	**12909**	**126**	OxA-430	Charred gazelle bone	Moore, 2000
455	2	E311	284.60	413	10930	120	**12848**	**105**	OxA-6685	Grain (dom. rye)	Moore, 2000
470	1	E326	284.20	446	10900	200	**12818**	**193**	OxA-172	Grain fragments of wild einkorn	Moore, 2000
470	1	E326	284.20	446	11070	160	**12945**	**140**	OxA-387	Charred *Bos* sp. bone	Moore, 2000
470	1	E326	284.20	446	11090	150	**12955**	**136**	OxA-468	*Bos* sp. bone, repeat of OxA-387	Moore, 2000
470	1	E326	284.20	446	11450	300	**13335**	**317**	OxA-883	Grain fragments of wild einkorn	Moore, 2000
435	1	ES15	284.40	395	(Undated)	--	--	--	--	--	--
468	1	E324	284.30	436	(Undated)	--	--	--	--	--	--
**Rejected dates: stratigraphically out of order**											
396	3		285.50	327	9060	140	**10190**	**214**	OxA-475	Charred gazelle bone	Moore, 2000
398	3	E254	285.40	337	9100	100	**10282**	**140**	BM-1719R	Charcoal	Moore, 2000
430	2		284.90	384	9600	200	**10936**	**288**	OxA-476	Fulvic fraction of OxA-434	Moore, 2000
449	2	E305	284.55	412	9860	220	**11384**	**379**	OxA-6996	Grain (domestic rye)	Moore, 2000
405	3		285.30	347	10600	200	**12426**	**262**	OxA-170	Grain fragments of wild einkorn	Moore, 2000
419	3		285.05	372	10610	100	**12538**	**127**	OxA-8719	Grain (domestic rye)	Moore, 2000
420	3	ES14	285.10	372	10800	160	**12718**	**171**	OxA-386	Grain fragments of wild einkorn	Moore, 2000
418	3		285.05	370	11140	100	**12982**	**108**	OxA-8718	Grain (domestic rye)	Moore, 2000
**Rejected dates: Phase 3, bone humic fraction**											
419	3		285.05	372	10250	160	**11972**	**306**	OxA-408	Humic fraction of OxA-407	Moore, 2000
430	2		284.90	384	10450	180	**12262**	**272**	OxA-435	Humic fraction of OxA-434	Moore, 2000
419	3		285.05	372	10620	150	**12490**	**189**	OxA-471	Humic fraction of OxA-407 (repeat)	Moore, 2000
460	2		284.55	330	10680	150	**12562**	**177**	OxA-431	Humic fraction of OxA-430	Moore, 2000
425	2		285.05	380	10750	170	**12645**	**197**	OxA-472	Humic fraction of OxA-473	Moore, 2000
470	1	E326	284.20	446	10820	160	**12743**	**165**	OxA-470	Humic fraction of OxA-468	Moore, 2000
470	1	E326	284.20	446	10920	140	**12844**	**121**	OxA-469	Humic fraction of OxA-468	Moore, 2000
429	2		284.90	376	10930	150	**12852**	**128**	OxA-474	Humic fraction of sheep bone	Moore, 2000

Table S2. Depths and abundances of Abu Hureyra impact proxies. "Level" = stratum # and "sample: = # assigned by Moore et al.[5]: magnetic spherules = MSp[23]; carbon spherules = CS[2]; nanodiamonds = NDs[2]; Meltglass[1]; floated organic material = Organics[5]; and seeds charred during cooking = Seeds[5].

Level #	Sample #	Depth (cm)	MSp (#/kg)	CS/kg	NDs (ppb)	Meltglass (g/kg)	Organics (mL/L)	Seeds (#/200 L)
**19**	**ES4**	82	0	15	0	0.00	--	--
**337**	**ES7**	192	0	0	0	0.00	--	--
**361**	**ES10**	202	0	0	0	0.02	--	--
**362**	**ES11**	222	0	0	0	0.04	--	--
**401-402**	**E256-259**	340	--	--	--	--	0.36	237
**405**	**--**	347	--	--	--	--	0.35	234
**402-406**	**ES14**	350	0	0	0	0.23	--	--
**411**	**--**	355	--	--	--	--	0.28	137
**412**	**--**	362	--	--	--	--	0.20	168
**417**	**E273**	370	--	0	--	--	--	--
**418**	**E274**	370	--	0	--	--	0.19	145
**419**	**--**	372	--	--	--	--	0.68	322
**420**	**--**	376	--	--	--	--	0.45	323
**423**	**E279**	380	--	0	--	--	--	--
**425**	**--**	380	--	--	--	--	1.02	97
**426**	**--**	380	--	--	--	--	0.12	265
**427**	**--**	382	--	--	--	--	--	--
**430**	**--**	384	--	--	--	--	0.71	422
**438**	**--**	400	--	--	--	--	0.27	191
**445**	**E301**	405	595	152	443	15.76	245.00	--
**449**	**E305**	412	--	20	--	--	0.90	926
**457**	**E313**	413	--	--	--	--	1.92	6044
**455**	**E311, E55**	413	--	--	--	--	1.44	2016
**435**	**ES15**	432	20	0	0	0.66	--	--
**467**	**--**	444	--	--	--	--	0.79	809
**468**	**E324**	445	--	--	--	--	0.35	692
**469**	**E325**	446	--	--	--	--	0.57	664
**470**	**E326**	446	--	--	--	--	0.25	596
**474**	**--**	446	--	--	--	--	1.01	1553
**471**	**E327**	450	--	--	--	--	0.78	677

Table S3. Representative SEM-EDS analyses of glass from Abu Hureyra. Shows keyed ms figure #s, elemental abundances, and total wt.%. Dashes indicate not present or not measured.

Fig.	Al	Au	C	Ca	Ce	Cr	Fe	Ir	K	La	Mg	Mn	Na	Nd	Ni	O	P	Pt	S	Si	Th	Ti	Zr	Total
1a-b	3.2	--	--	4.1	--	0.0	11.7	--	1.7	--	1.8	0.1	0.4	--	30.0	33.3	0.7	--	0.4	12.2	--	0.6	--	100.1
1c-d	4.6	--	18.7	3.8	--	--	14.9	--	1.4	--	1.1	--	0.8	--	--	37.8	--	--	1.2	14.6	--	1.1	--	100.0
1c-d	3.3	--	--	2.9	--	7.8	36.3	--	0.8	--	0.5	0.1	0.4	--	3.8	32.7	0.0	--	0.2	10.9	--	0.2	--	100.0
1e-f	9.9	--	--	5.8	--	0.0	2.8	--	3.6	--	0.5	0.0	2.1	--	0.0	46.4	0.1	--	0.4	27.6	--	0.7	--	100.0
1e-f	9.3	--	--	7.5	--	0.0	2.6	--	7.5	--	0.3	0.3	3.7	--	0.1	43.7	0.0	--	0.1	24.9	--	0.1	--	100.0
2a	2.0	--	--	5.1	--	0.1	2.6	--	1.7	--	1.1	0.0	0.7	--	0.2	49.0	0.0	--	1.0	36.3	--	0.2	--	100.1
2b	5.3	--	--	12.3	--	0.0	4.9	--	1.3	--	3.4	0.0	1.4	--	0.0	44.5	0.6	--	0.0	25.9	--	0.3	--	100.0
2b	1.1	--	--	1.0	--	0.0	1.6	--	0.9	--	0.8	0.2	0.3	--	0.2	51.3	0.2	--	0.0	42.4	--	0.2	--	100.0
2b	2.2	--	--	1.5	--	0.2	2.1	--	1.7	--	0.8	0.2	0.5	--	0.0	50.3	0.0	--	0.1	40.2	--	0.2	--	100.0
2b	5.0	--	--	12.2	--	0.2	3.5	--	1.4	--	3.4	0.1	1.3	--	0.0	45.1	0.2	--	0.4	26.8	--	0.5	--	100.0
2b	5.3	--	--	12.3	--	0.0	4.9	--	1.3	--	3.4	0.0	1.4	--	0.0	44.5	0.6	--	0.0	25.9	--	0.3	--	100.0
2d	4.3	--	--	8.8	--	0.0	4.7	--	2.9	--	3.7	0.5	2.9	--	0.1	44.3	0.0	--	0.0	27.3	--	0.6	--	100.2
2e	6.7	--	--	12.6	--	0.5	5.8	--	2.9	--	2.7	0.0	2.5	--	0.0	43.2	0.5	--	0.3	22.6	--	0.6	--	100.9
2f	3.6	--	22.7	3.9	--	0.1	2.1	--	2.6	--	1.2	0.0	1.5	--	0.0	45.5	0.2	--	0.1	16.0	--	0.4	--	100.0
2f	0.9	--	24.2	1.6	--	0.0	0.4	--	0.4	--	0.7	0.0	1.1	--	0.0	50.6	0.2	--	0.2	19.7	--	0.0	--	100.0
2f	2.0	--	25.8	4.1	--	0.0	1.2	--	1.1	--	1.6	0.0	1.0	--	0.0	45.6	0.1	--	0.3	17.2	--	0.1	--	100.0
2g	4.5	--	--	1.5	--	0.0	3.7	--	3.4	--	1.5	0.3	1.6	--	0.1	48.0	0.0	--	0.3	35.8	--	0.5	--	101.2
2h	4.0	--	--	15.1	--	0.0	8.5	--	1.0	--	5.5	0.3	0.7	--	0.0	42.3	0.7	--	0.3	21.5	--	0.6	--	100.3
2i	2.6	--	18.8	13.5	--	--	2.1	--	1.0	--	6.0	--	0.5	--	--	31.1	0.4	--	--	23.6	--	0.5	--	100.0
2i	1.6	--	--	13.2	--	0.0	5.7	--	0.3	--	8.8	0.1	1.9	--	0.1	43.3	0.2	--	0.0	24.6	--	0.4	--	100.0
2i	1.6	--	--	13.1	--	0.0	5.7	--	0.3	--	8.8	0.1	1.9	--	0.1	43.3	0.2	--	0.0	24.6	--	0.4	--	100.0
3d-h	5.1	--	--	0.5	--	0.0	6.2	--	0.7	--	3.8	0.4	1.3	--	0.1	48.1	0.5	--	0.2	32.4	--	0.6	--	100.0
3e-i	3.3	--	--	0.9	--	0.1	6.5	--	0.6	--	2.3	0.2	0.3	--	0.3	48.6	0.6	--	0.2	34.7	--	1.7	--	100.0
3f-j	2.2	--	--	1.0	--	0.2	10.7	--	0.9	--	2.1	3.3	0.5	--	0.0	45.8	0.9	--	0.3	30.8	--	1.4	--	100.0
3g-k	2.5	--	--	1.0	--	0.0	6.5	--	0.7	--	1.9	0.1	0.0	--	0.0	49.2	0.7	--	0.1	36.9	--	0.6	--	100.0
4a	4.6	--	--	12.9	--	0.1	2.9	--	2.0	--	2.9	0.0	1.8	--	0.1	44.7	0.3	--	0.0	27.2	--	0.5	--	100.0
4b	5.3	--	--	5.4	--	0.1	3.9	--	5.5	--	1.4	0.0	2.8	--	0.0	45.4	0.1	--	0.9	28.4	--	0.8	--	100.0
4c	4.5	--	--	5.2	--	0.0	4.7	--	7.0	--	2.5	0.0	1.6	--	0.0	44.7	0.4	--	0.0	28.8	--	0.5	--	100.0
4c	3.1	--	--	14.8	--	0.2	10.4	--	4.6	--	4.1	0.1	1.6	--	0.0	39.8	0.3	--	0.0	20.1	--	0.8	--	100.0
5a	1.5	--	--	0.9	--	0.1	3.2	--	0.3	--	1.3	0.1	0.2	--	0.3	50.7	0.4	--	0.2	40.2	--	0.5	--	100.0
5a	2.7	--	--	1.3	--	0.0	3.1	--	0.5	--	0.6	0.2	0.2	--	0.0	50.7	0.4	--	0.1	40.0	--	0.2	--	100.0
5c-d	2.9	--	--	6.1	--	0.0	6.1	--	5.6	--	10.6	0.3	1.8	--	0.1	42.7	0.5	--	0.3	22.6	--	0.3	--	100.0
6	0.0	--	0.0	0.0	--	0.0	0.0	--	0.0	--	0.0	0.0	0.0	--	0.0	53.3	0.0	--	0.0	46.7	--	0.0	--	100.0
6	2.8	--	0.0	3.2	--	0.3	1.8	--	0.5	--	0.8	0.0	0.3	--	0.0	47.8	0.2	--	0.1	41.9	--	0.2	--	100.0
6	3.8	--	0.0	3.7	--	0.0	0.8	--	0.8	--	1.5	1.9	0.8	--	0.7	45.4	0.2	--	0.4	39.7	--	0.3	--	100.0
6	5.5	--	0.0	10.4	--	1.4	7.8	--	0.7	--	4.0	0.2	1.4	--	0.1	36.2	0.2	--	0.1	31.8	--	0.2	--	100.0
7	1.2	--	--	0.6	--	--	1.2	--	0.6	--	0.6	--	0.4	--	--	33.4	--	--	--	17.3	--	--	44.6	100.0
7	0.0	--	0.0	0.0	--	0.0	0.0	--	0.0	--	0.0	--	0.0	--	--	36.7	--	--	--	16.2	--	--	47.1	100.0
8a-b	8.4	--	--	1.6	--	8.8	24.7	--	0.4	--	3.2	--	--	--	--	43.6	--	--	0.3	8.7	--	0.3	--	100.0
8a-b	10.0	--	--	1.5	--	9.4	26.6	--	0.5	--	4.0	--	--	--	--	37.0	--	--	0.4	10.4	--	0.4	--	100.2
8a-b	--	--	--	--	--	12.9	36.4	--	--	--	--	--	--	--	--	50.7	--	--	--	--	--	--	--	100.0
8c	3.5	21.7	17.7	1.5	--	5.5	35.0	--	0.5	--	--	--	--	--	--	9.1	--	--	--	5.5	--	--	--	100.0
9	3.0	--	11.6	2.6	--	6.9	48.2	--	1.3	--	--	--	1.9	--	--	13.4	--	--	1.6	8.9	--	--	--	99.3
9	--	--	--	--	--	12.5	87.5	--	--	--	--	--	--	--	--	--	--	--	--	--	--	--	--	100.0
10a,e	1.9	--	--	1.9	--	--	43.4	--	1.7	--	1.3	--	--	--	0.3	34.7	--	--	--	14.9	--	--	--	100.0
10a,e	1.9	--	--	1.5	--	41.1	1.7	--	1.3	--	--	--	--	--	0.5	34.8	--	--	--	17.1	--	--	--	100.0
10a,e	3.5	--	--	1.7	--	--	--	--	3.6	--	2.8	--	--	--	--	49.6	--	--	--	38.8	--	--	--	100.0
11a-d	1.3	--	--	3.9	--	--	61.9	--	--	--	--	--	--	--	--	23.7	--	--	--	9.3	--	--	--	100.0
11e	2.8	--	--	3.2	--	0.2	38.2	--	0.5	--	2.4	0.1	0.8	--	0.3	33.5	0.2	--	0.1	9.4	--	8.3	--	100.0
11e	--	--	--	--	--	--	46.3	--	--	--	2.9	--	--	--	--	40.7	--	--	--	--	--	10.1	--	100.0
11f	1.3	--	--	2.5	--	--	69.6	--	1.0	--	1.2	--	1.2	--	--	13.6	--	--	--	9.7	--	--	--	100.0
11f	--	--	--	--	--	--	82.9	--	--	--	--	--	--	--	--	16.2	--	--	--	--	--	--	--	99.1
11f	--	--	--	1.6	--	--	64.6	--	--	--	--	--	--	--	--	22.5	--	--	--	11.3	--	--	--	100.0
12b	0.9	--	--	1.6	--	0.0	57.5	--	0.7	--	0.9	0.4	0.3	--	2.0	28.1	0.1	--	0.1	7.1	--	0.2	--	100.0
12c	4.7	--	--	3.2	--	0.1	9.7	--	2.1	--	2.6	0.2	1.1	--	19.5	37.7	0.3	--	0.2	17.8	--	0.8	--	100.0
12e	3.9	--	--	18.2	--	0.0	4.3	--	1.7	--	2.6	0.0	1.4	--	0.0	42.9	0.4	--	0.4	23.6	--	0.5	--	100.0
12e	3.9	--	--	18.2	--	0.0	4.3	--	1.7	--	2.6	0.0	1.4	--	0.0	42.9	0.4	--	0.4	23.6	--	0.5	--	100.0
12e	0.9	--	--	5.9	--	0.1	52.6	--	0.5	--	0.4	0.0	0.7	--	0.4	29.5	0.6	--	0.3	8.3	--	0.0	--	100.0
12f	3.2	--	44.3	7.6	--	--	6.7	0.8	1.5	--	0.5	--	--	--	--	24.0	--	2.5	--	8.9	--	--	--	100.0
12f	--	--	--	--	--	--	56.3	6.9	--	--	--	--	--	--	--	16.0	--	20.9	--	--	--	--	--	100.1
12f	2.9	--	43.1	7.4	--	--	5.8	0.4	1.3	--	0.5	--	0.3	--	--	19.5	--	0.6	--	9.7	--	0.4	8.3	100.0
13a	2.9	--	--	4.2	--	0.3	32.0	--	2.0	--	2.0	1.3	0.5	--	0.0	36.5	0.3	--	0.3	16.9	--	0.8	--	100.0
13b	2.9	--	--	4.2	--	0.3	31.9	--	2.0	--	2.0	1.3	0.5	--	0.0	36.5	0.3	--	0.3	16.9	--	0.8	--	100.0
14a	4.3	--	--	10.9	--	--	2.9	--	2.0	--	2.4	--	1.1	--	--	46.0	0.3	--	0.2	29.6	--	0.4	--	100.0
14a-c	1.0	--	--	0.2	--	0.1	11.9	--	0.0	--	2.1	0.1	0.3	--	18.5	43.5	0.0	--	19.7	2.5	--	0.1	--	100.0
14c	--	--	--	--	--	--	37.7	--	--	--	--	--	--	--	--	--	--	--	62.4	--	--	--	--	100.1
15	9.8	--	--	3.8	--	0.1	3.5	--	3.5	--	1.4	0.1	2.8	--	0.1	46.4	0.1	--	0.1	28.0	--	0.3	--	100.0
15	0.7	--	--	3.0	--	0.0	61.3	--	0.5	--	0.1	0.0	0.0	--	0.2	27.5	0.0	--	1.3	4.9	--	0.6	--	100.0
15a-b	--	--	--	--	29.2	--	--	--	--	14.5	--	--	--	12.0	--	26.6	12.9	--	--	--	4.8	--	--	100.0

Table S4. Representative microprobe measurements of glass from Abu Hureyra and other sources. Shows sites, sample numbers, number of spot measurements, and oxide percentages.

Site	Sample	Spot	SiO2	TiO2	Al2C	FeO	MgC	MnC	CaO	Na2(	K2O	Cl	SO3	Cr2O3	V2O3	Total
ABU E-B11	abu-1-pyx-1	4	46.35	0.79	5.34	8.33	13.17	0.07	24.31	0.54	0.19	0.00	0.00	0.00	0.00	99.09
ABU E-B12	abu-1-pyx-2	5	45.82	0.91	5.66	9.86	12.60	0.09	23.79	0.57	0.15	0.00	0.00	0.00	0.00	99.46
ABU E-B14	abu-1-gl-1	7	52.70	1.10	11.18	5.06	1.89	0.15	21.00	2.28	2.35	0.01	0.00	0.00	0.00	97.74
ABU E-B15	abu-1-gl-2	8	50.99	1.03	11.44	4.76	2.05	0.09	21.44	2.24	2.29	0.00	0.00	0.00	0.00	96.33
ABU E-B16	abu-1-gl-3	9	49.38	0.84	10.72	5.16	2.02	0.15	23.37	2.07	2.04	0.00	0.04	0.00	0.00	95.81
ABU E-B17	abu-1-gl-4	10	50.91	1.42	10.47	6.55	2.39	0.21	18.10	2.24	3.40	0.00	0.04	0.00	0.00	95.74
ABU E-B18	abu-1-gl-5	11	48.35	0.75	11.87	5.20	2.09	0.15	23.35	2.25	2.22	0.00	0.04	0.00	0.00	96.27
Blackville	BC-glass.1	6	58.51	1.46	20.26	8.89	2.02	0.03	3.40	0.50	3.39	0.02	0.00			98.49
Blackville	BC-glass.2	7	58.36	1.45	20.22	8.94	1.99	0.03	3.61	0.43	3.32	0.01	0.02			98.39
Blackville	BC-glass.3	8	56.70	1.60	20.95	9.58	2.13	0.00	3.71	0.50	3.24	0.00	0.00			98.41
Blackville	BC-glass.4	9	56.55	1.46	20.95	9.52	2.02	0.01	3.75	0.44	3.26	0.00	0.00			97.95
Blackville	BC-glass.5	10	57.85	1.35	20.47	7.84	1.90	0.02	3.68	0.57	3.51	0.00	0.00			97.19
Blackville	BC-glass.6	11	54.72	1.37	20.74	8.86	2.12	0.05	4.19	0.47	3.18	0.00	0.01			95.71
Blackville	BC-glass.7	12	53.10	1.56	20.94	9.23	2.22	0.04	4.39	0.44	2.91	0.00	0.00			94.83
Dakhleh	DG-glass.1	21	62.54	0.73	14.63	5.22	0.27	0.02	3.04	3.28	2.68	0.27	0.00			92.68
Dakhleh	DG-glass.2	22	61.12	0.91	14.12	5.91	0.40	0.00	3.31	2.63	1.97	0.32	0.05			90.75
Dakhleh	DG-glass.3	23	59.26	0.89	17.55	4.33	0.68	0.00	3.72	5.08	4.27	0.20	0.09			96.07
Dakhleh	DG-glass.4	24	60.16	0.88	16.65	4.26	0.45	0.03	2.93	4.72	4.48	0.18	0.01			94.75
Dakhleh	DG-diop.1	31	49.74	0.16	0.67	9.57	13.89	0.12	23.39	1.16	0.09	0.00	0.02			98.80
Dakhleh	DG-diop.2	32	50.29	0.15	0.72	9.41	13.57	0.15	23.31	1.07	0.09	0.00	0.12			98.89
Dakhleh	DG-diop.3	33	49.69	0.16	0.85	11.08	13.31	0.11	23.35	0.99	0.05	0.00	0.00			99.58
Dakhleh	DG-diop.4	34	48.98	0.33	0.97	12.98	12.41	0.17	22.68	1.26	0.07	0.01	0.01			99.86
Fulgurite	F-gl-1	12	45.35	0.58	11.00	4.12	1.11	0.07	36.90	0.60	1.26	0.00	0.00	0.00	0.00	100.99
Fulgurite	F-gl-2	13	45.36	0.55	11.00	4.01	1.06	0.07	36.85	0.52	1.27	0.05	0.00	0.00	0.00	100.74
Fulgurite	F-gl-3	14	45.27	0.59	10.98	3.93	0.99	0.07	36.65	0.53	1.25	0.02	0.00	0.00	0.00	100.27
Fulgurite	F-gl-4	15	45.15	0.51	10.87	4.01	1.07	0.10	36.65	0.46	1.26	0.00	0.00	0.00	0.00	100.09
Fulgurite	F-gl-5	16	44.91	0.61	10.88	3.93	1.05	0.05	36.85	0.53	1.27	0.00	0.00	0.00	0.00	100.08
Melrose	M-bulk	17	49.28	0.90	17.61	29.18	0.52	0.01	0.27	0.00	0.00	0.00	0.09	0.03	0.02	97.92
Melrose	M-bulk	18	44.11	1.06	20.88	30.57	0.52	0.01	0.25	0.00	0.00	0.00	0.05	0.01	0.06	97.51
Melrose	M-bulk	19	46.24	0.96	18.67	30.01	0.47	0.00	0.26	0.00	0.00	0.00	0.00	0.01	0.00	96.62
Melrose	M-bulk	20	49.58	0.87	15.74	29.48	0.45	0.05	0.39	0.00	0.00	0.00	0.03	0.00	0.01	96.61
Melrose	M-bright	21	0.86	0.89	27.78	68.74	0.58	0.01	0.00	0.00	0.00	0.00	0.06	0.49	0.11	99.52
Melrose	M-bright	23	0.15	1.53	29.34	67.39	0.56	0.00	0.00	0.00	0.00	0.00	0.04	0.17	0.09	99.29
Melrose	M-bright	24	0.19	1.00	27.13	70.74	0.52	0.01	0.00	0.00	0.00	0.00	0.01	0.22	0.09	99.91
Melrose	M-bright	25	0.08	0.32	7.27	84.95	1.60	0.04	0.01	0.00	0.00	0.00	0.00	0.00	0.06	94.33
Melrose	M-dark glass	35	48.60	0.88	14.53	31.44	0.48	0.01	0.36	0.16	2.15	0.02	0.01	0.00	0.00	98.65
Ries	RC-glass mn	36	61.98	0.87	15.62	5.04	2.90	0.12	3.31	2.46	3.09	0.02	0.00	0.00	0.00	95.41
Ries	RC-glass mn	38	61.53	0.87	15.34	4.60	2.73	0.10	3.32	2.38	3.18	0.02	0.00	0.00	0.00	94.05
Ries	RC-glass mn	39	61.74	0.85	15.14	4.39	2.47	0.04	3.12	2.50	3.18	0.01	0.00	0.00	0.00	93.44
Ries	RC-glass mn	40	62.86	0.76	15.29	4.61	2.54	0.07	3.17	0.62	2.04	0.00	0.00	0.00	0.00	91.95
Ries	RC-glass mn	41	58.76	1.17	16.03	6.32	3.62	0.10	4.99	2.18	2.56	0.01	0.00	0.00	0.00	95.75
Ries	RC-glass mn	42	60.87	0.96	16.10	5.57	3.08	0.09	3.73	2.38	2.92	0.00	0.01	0.00	0.00	95.71
Ries	RC-glass mn	43	60.73	1.00	15.67	5.62	3.17	0.11	4.21	2.33	2.70	0.00	0.04	0.00	0.00	95.57
Ries	RC-glass mn	44	60.84	1.00	15.75	5.64	3.07	0.07	4.26	2.33	2.78	0.01	0.00	0.00	0.00	95.75
Power plant	PP-glass.1	41	46.37	1.36	17.16	11.99	3.17	0.04	15.16	0.99	0.63	0.02	0.00			96.88
Power plant	PP-glass.2	42	46.77	1.31	17.21	12.09	3.14	0.04	15.08	0.92	0.65	0.01	0.05			97.28
Power plant	PP-glass.3	43	45.39	1.45	17.42	10.81	3.28	0.03	15.58	0.98	0.62	0.00	0.00			95.57
Power plant	PP-glass.4	44	45.63	1.49	17.31	10.79	3.55	0.03	16.33	0.99	0.62	0.02	0.00			96.76
Power plant	PP-glass.5	45	46.03	1.38	17.15	11.33	3.48	0.00	16.27	0.95	0.61	0.00	0.00			97.19
Power plant	PP-glass.6	46	45.60	1.44	17.17	10.38	3.64	0.00	16.62	0.92	0.61	0.02	0.00			96.40
Power plant	PP-glass.7	47	45.78	1.49	17.27	10.73	3.55	0.02	16.67	0.93	0.64	0.02	0.02			97.11
Power plant	PP-glass.8	48	46.02	1.56	16.97	10.88	3.54	0.00	16.53	0.93	0.68	0.03	0.02			97.17
Slag	SS-glass.1	74	46.22	0.53	17.29	0.03	4.83	0.61	27.95	0.09	2.80	0.01	0.16			100.52
Slag	SS-glass.2	75	46.00	0.55	17.28	0.00	4.79	0.58	28.04	0.09	2.83	0.02	0.18			100.37
Slag	SS-glass.3	76	46.24	0.51	17.26	0.03	4.86	0.61	27.94	0.07	2.85	0.00	0.16			100.51
Slag	SS-glass.4	77	44.03	0.43	17.27	0.04	4.68	0.63	27.59	0.12	2.83	0.00	0.16			97.80
Slag	SS-glass.5	78	44.13	0.56	17.11	0.05	4.73	0.58	27.51	0.15	2.78	0.03	0.16			97.79
Slag	SS-glass.6	79	46.13	0.47	17.17	0.04	4.83	0.59	27.72	0.13	2.78	0.01	0.22			100.06
Slag	SS-glass.7	80	46.31	0.45	17.15	0.00	4.77	0.56	28.08	0.14	2.84	0.01	0.11			100.43
Trinitite	TAB-glass.1	84	65.21	0.47	11.45	2.86	1.08	0.06	9.98	1.44	2.81	0.00	0.02			95.37
Trinitite	TAB-glass.2	85	63.67	0.50	11.66	2.92	1.16	0.07	10.63	1.41	2.72	0.03	0.00			94.78
Trinitite	TAB-glass.3	86	63.53	0.49	11.79	2.93	1.19	0.04	10.83	1.49	2.80	0.00	0.07			95.18
Trinitite	TAB-glass.4	87	59.54	0.50	11.12	2.69	0.99	0.08	9.91	1.42	2.79	0.00	0.00			89.04
Trinitite	TAB-glass.5	88	56.31	0.55	10.69	2.42	0.92	0.07	9.35	1.40	2.68	0.02	0.00			84.42
Trinitite	TAB-glass.6	89	59.50	0.56	12.40	3.70	1.59	0.06	13.90	1.51	2.70	0.02	0.03			95.96
Trinitite	TAB-glass.7	90	64.41	0.62	11.76	3.24	1.22	0.04	10.77	1.68	2.88	0.04	0.04			96.71

Table S5. Time and temperature for furnace experiments. "Sample" = # assigned by Moore et al. 2000. "Set T" = experimental target temperatures. "Insertion T" = actual temperature at start of experiment. "Overshoot T" = actual maximum temperature of experiment. "Time to target T" = ramp-up to maximum temperature.

Sample	Set T (°C)	Box furnace #	Insertion T (°C)	Overshoot T (°C)	Overshoot time	Time to target T
**ES15-H**	1100	2		1107	10 sec	
**ES15-O**	1150	1	1117	1150		30 sec
**ES15-E**	1200	2		1204		
**ES15-N**	1250	1	1217	1250		40 sec
**ES15-G**	1300	2		1307	30 sec	
**ES15-F**	1400	2		1420	30 sec	
**ES15-I**	1500	2	1400	1533	60 sec	180 sec
**ES15-J**	1500	1	1400	1500		60 sec
**ES15-L**	1600	1	1500	1500		60 sec
**ES15-K**	1700	1	1550	1700		180 sec
**ES1-5M**	1700	1	1500	1700		8 min
**ES15 3/3**	1200	1	1180	1200		30 sec
**ES15 2/2**	1400	1	1350	1400		45 sec
**ES15 5/5**	1500	1	1440	1500		90 sec
**ES15 6/6**	1500	1		1500		
**ES15 1/1**	1600	1	1500	1600		90 sec
**ES15 7/7**	1700	1	1500	1700		9 min

Table S6. Reflectance values. Information on various organic materials tested, including collection site, material, maceral, and the number of reflectance test measurements. Shows average reflectance value (%R_o); error (s.d.); minimum; maximum; known/inferred temperatures; and inferred temperature differential. The maximum temperature differential was ~1069°C with an average of 487°C. Temperatures interpolated from Table 2 of Ascough et al.[20].

#	Sample Name	Site	Material	Maceral	No. tests	Avg R_o	Err	Avg Temp	Min R_o	Min Temp	Max R_o	Max Temp	Time (min)	Known Temp	Δ Temp
1	**AH Charcoal**	Abu Hureyra, Syria	Charcoal	fusinite	15	1.01	0.09	391	0.89	380	1.19	409	unk	≈391°C	0
2	**AH Glass +Char**	Abu Hureyra, Syria	Melt glass + charred matter	char	11	1.33	0.16	421	1.03	391	1.56	440	unk	>1250°C	829
3	**Reed Charcoal**	Rochester, NY	Charcoal from reeds	fusinite	32	4.24	0.37	632	3.60	591	5.16	690	<1	>1700°C	1068
4	**Oak Charcoal**	Prescott, AZ	Charcoal from oak	fusinite	28	4.23	0.27	631	3.63	593	4.76	665	<1	>1700°C	1069
5	**Pine Charcoal**	Prescott, AZ	Charcoal from pine	fusinite	21	4.49	0.29	648	3.70	597	4.95	677	<1	>1700°C	1052
6	**Trinitite +Charcoal**	Socorro, NM	Charcoal from Trinity site	fusinite	36	1.16	0.28	406	0.66	357	1.51	436	unk	>1250°C	844
7	**Calgon Act Carbon**	Calgon Corp.	Act carbon (OLC 12x40)	fusinite	31	3.51	0.15	585	2.55	518	4.38	641	≈480	≈1100°C	515
8	**Calgon Char**	Calgon Corp.	Coconut char (OLC 12x40)	fusinite	25	2.45	0.45	511	2.12	486	2.76	532	≈480	≈450°C	0
9	**Gainey CS heated**	Gainey, MI	Carbon spherules, heated	cell walls	28	2.82	0.32	537	2.36	504	3.44	580	≈5	≈730°C	193
10	**Kimbel CS heated**	Kimbel Bay, NC	Carbon spherules, heated	cell walls	25	2.51	0.52	515	1.13	403	3.55	588	≈5	≈700°C	185
11	**Indian Crk CS heated**	Prescott, AZ	Carbon spherules, heated	cell walls	73	3.26	0.7	567	2.16	489	3.92	611	≈5	≈650°C	83
12	**Tunguska Charcoal**	Tunguska, Russia	Charcoal from Tunguska	fusinite	35	1.24	0.13	413	1.05	395	1.47	432	unk	≈413°C	0

Table S7. Elemental results of Abu Hureyra sediment and magnetic grains, using INAA, fire assay, and ICP-MS (ActLabs). Samples with the suffix of "-m" represents magnetic fractions; "-glass" represent pieces of excavated AH glass; other samples are AH bulk sediment. Depths and abundances of Abu Hureyra impact proxies: cobalt = Co; chromium = Cr; nickel = Ni; and iron = Fe, with values in ppm. Palladium = Pd; platinum = Pt; and gold =Au, with values in ppb. Dark green highlighting indicates peak concentrations in the YDB layer, sample E301 at 405 cm.

Sample	Type	D (cm)	Co	Cr	Ni	Fe	Pt	Pd	Au	Pt/Pd	Pt/Au
Mag-ES4	Mag	82	37	744	20	58100	--	--	2.0	--	--
Mag-ES7	Mag	192	33	454	20	46000	--	--	2.0	--	--
Mag-ES10	Mag	202	35	601	20	58000	--	--	2.0	--	--
Mag-ES11	Mag	222	34	629	20	56300	--	--	2.0	--	--
Mag-E48	Mag	295	38	576	20	59600	--	--	2.0	--	--
Mag-E255	Mag	337	29	572	20	43600	--	--	2.0	--	--
Mag-E256-259	Mag	340	30	411	20	44700	--	--	2.0	--	--
Mag-ES14-m	Mag	350	42	2020	125	93900	1.1	1.4	2.0	0.8	0.6
Mag-ES14	Mag	350	28	805	20	52500	--	--	2.0	--	--
Mag-E279	Mag	380	33	964	20	53100	--	--	2.0	--	--
Mag-E301-1	Mag	405	42	2660	200	87500	--	--	--	--	--
Mag-E301-2	Mag	405	68	3750	300	195000	8.1	1.6	3.0	5.1	2.7
Mag-E301-glass	Mag	405	27	561	20	56800	1.1	1.4	2.0	0.8	0.6
Mag-ES15	Mag	432	25	1020	20	52500	--	--	2.0	--	--
Mag-ES15-2	Mag	432	47	2770	20	110000	--	--	2.0	--	--
Mag-E55	Mag	446	29	797	20	49100	--	--	2.0	--	--
ES4	Sed	82	27	460	20	37800	1.6	1.2	4.0	1.3	0.4
ES7	Sed	192	25	383	20	36000	1.5	1.0	2.0	1.5	0.8
ES10	Sed	202	25	418	20	39600	1.1	1.3	3.0	0.8	0.4
ES11	Sed	222	24	378	20	35600	1.3	1.3	2.0	1.0	0.7
E48	Sed	295	27	391	20	39900	1.6	1.5	4.0	1.1	0.4
E255	Sed	337	24	428	20	34100	1.2	1.2	2.0	1.0	0.6
E256-259	Sed	340	21	336	20	31700	1.2	1.2	2.0	1.0	0.6
ES14	Sed	350	16	446	20	29200	0.7	1.1	2.0	0.6	0.4
ES14-2	Sed	350	22	701	20	40000	0.7	1.1	2.0	0.6	0.4
E279	Sed	380	19	418	20	22800	1.2	1.2	2.0	1.0	0.6
E301	Sed	405	18	261	20	23900	6.2	1.3	2.5	4.8	2.5
ES15	Sed	432	20	479	20	30400	1.1	1.4	4.0	0.8	0.3
ES15-2	Sed	432	19	494	20	33300	1.1	1.4	4.0	0.8	0.3
E55	Sed	446	20	480	20	34000	0.9	1.2	3.0	0.8	0.3

Table S8. Remanent magnetism values. Sources and materials. Mechanisms: TRM = conventional natural magnetization mechanisms (thermoremanent magnetization); NRM = natural rock samples; LRM = lightning-induced remanent magnetization. Values are reported as 'efficiency of magnetic intensity.' References: Wasilewski[63-65]; Parry[66]; Lewis[67]; Kletetschka[68].

Sources	Material	Mechanism	Efficiency (hi)	Efficiency (lo)	Efficiency (avg)	Reference
Abu Hureyra	Meltglass	TRM	0.02	0.002	0.011	This study
Terrestrial 1	FeNi Spheres	TRM	0.03	0.0012	0.0156	Wasilewski, 1981
Terrestrial 2	Magnetite 1	TRM	0.0043	0.001	0.00265	Wasilewski 1999
Terrestrial 3	Magnetite 2	TRM	0.018	0.018	0.018	Parry, 1982
Terrestrial 4	Titanomagnetite	TRM	0.01	0.007	0.0085	Lewis, 1968
Meteorite	Murchison	NRM	0.0009	0.0001	0.0005	Kletetschka 2003
Lightning 1	94ADK2	LRM	0.83	0.83	0.83	Wasilewski 1999
Lightning 2	Lodestones	LRM	0.69	0.14	0.415	Wasilewski 1977
Lightning 3	Fulgurites	LRM	0.69	0.45	0.57	Wasilewski 1999

Table S9. The magnetization of Abu Hureyra meltglass and other materials. Shows type of material, site name, location, and the number of samples tested. Field strength indicates the level of remanent magnetism, along with the number of vectors, rotational history, and the inferred source.

Type	Site	Location	Type	Samples	Field strength	Vectors	Rotating/ Stable	Possible source
YDB spherules	Abu Hureyra, E301	Syria	YDB melted spherules	6	Normal	1	Stable	Airburst/impact event?
YDB glass	Abu Hureyra, E311	Syria	YDB meltglass	3	Normal	1	Stable	Airburst/impact event?
	Blackville, Core-12	SC	YDB meltglass	1	Moderate	1	Stable	Airburst/impact event?
	Melrose, Y-1	PA	YDB meltglass #1	3	Demag.	Many	Rotating	Airburst/impact event?
	Melrose, Y-1	PA	YDB meltglass #2	2	Strong	1	Stable	Lightning/Impact lightning?
Tektites	Indochinite, layered	Laos	Australasian tektites	2	Normal	1	Stable	Airburst or impact event?
	Indochinite, layered	Laos	Australasian tektites	1	Strong	1	Stable	Airburst or impact event?
	Muong Nong	Laos	Australasian tektites	1	Mod/weak	1	Stable	Airburst or impact event?
Impact glass	Argentine escoria	Argentina	Impact melt	1	Normal	1	Stable	Shock from impact?
	Dakhleh glass	Egypt	Meltglass from airburst	1	Demag.	2	Moving	Airburst or impact event?
	Darwin glass	Australia	Impact glass	1	Weak	1	Stable	Shock from impact?
	Houghton Crater	Canada	Impact-melted rocks	1	Weak	2	Moving	Shock from impact?
	Meteor Crater	AZ	Impact glass	1	Demag.	3	Rotating	Shock from impact?
	Monturaqui Crater	Chile	Impact glass	1	Strong	2	Rotating	Lightning/Impact lightning?
	Ries Crater	Germany	Impact-melted suevite	1	Normal	1	Stable	Shock from impact?
	Zhaminshin	Kazakhstan	Impact-melted glass	1	Moderate	1	Stable	Shock from impact?
Fulgurites	Black, anthill	NM	Lightning-formed glass	1	Strong	1	Stable	Lightning
	Tan	AZ	Lightning-formed glass	1	Strong	Many	Rotating	Lightning
Volcanics	Obsidian	AZ	Classic obsidian	2	Strong	1	Stable	Volcanic lightning?
Atomic glass	Sedan crater, tuff	NV	Undergrnd.atomic test	1	Moderate	1	Stable	EM pulse in detonation?
	Sedan crater, granite	NV	Undergrnd.atomic test	1	Moderate	1	Stable	EM pulse in detonation?
	Trinitite, dark	NM	Glass-atomic bomb	1	Moderate	2	Moving	EM pulse in detonation?
Anthropogenic	Power plant slag	LA	Coal-fired power plant	1	Strong	2	Moving	Anthropogenic
	Railroad slag	SC	South Carolina Railroad	1	Moderate	1	Stable	Anthropogenic
	Smelting slag	MI	Foundry ore	1	Demag.	1	Moving	Anthropogenic

Table S10. Water content. Test materials showing low and high ppm of H_2O. Columns 2-3 were measured in this study; columns 4-5 by others. References: Beran and Koeberl[35]; Heide[37]; Watt[38]; Harris[69]; Glass[39]; Bouska[40]; Dixon[43]; Koeberl[45]; Thy[48]; Jacobson[49]; Takata[51]; and Zajacz[52].

Test Materials	Low ppm (here)	High ppm (here)	Low ppm (others)	High ppm (others)	References
AH glass	222	460	--	--	This study
Reed glass	229	329	--	--	This study
Tektites	78	227	20	500	Beran & Koeberl 1997
Impact glass	611	1036	80	240000	Heide 2011, Watt 2011, Harris 2007
Trinity glass	283	510	70	100	Glass 1987
Fulgurites	--	159	500	1400	Heide 2011, Bouska 1993
Volcanic glass	1497	1769	500	4000	Dixon 1988, Koeberl 1992
Biomass glass	--	--	1000	9100	Thy 1995, Jacobson 2003
Anthro. glass	--	--	500	120000	Takata 1992, Zajacz 2005

References

1 Bunch, T. E. *et al.* Very high-temperature impact melt products as evidence for cosmic airbursts and impacts 12,900 years ago. *Proc Nat Acad Sci* **109**, E1903-E1912 (2012).

2 Kinzie, C. R. *et al.* Nanodiamond-rich layer across three continents consistent with major cosmic impact at 12,800 cal BP. *J Geol* **122**, 475-506 (2014).

3 Wolbach, W. S. *et al.* Extraordinary biomass-burning episode and impact winter triggered by the Younger Dryas cosmic impact ~12,800 years ago. 2. Lake, marine, and terrestrial sediments. *J Geol* **126**, 185-205 (2018).

4 Kennett, J. P. *et al.* Bayesian chronological analyses consistent with synchronous age of 12,835-12,735 Cal B.P. for Younger Dryas boundary on four continents. *Proc Nat Acad Sci* **112**, E4344-4353 (2015).

5 Moore, A. M. T., Hillman, G. C. & Anthony, J. *Village on the Euphrates: from foraging to farming at Abu Hureyra*. 585 (Oxford University Press, 2000).

6 Kracek, F. C. Melting and transformation temperatures of mineral and allied substances. *Geol Soc Am, Special Papers* **36**, 139-174 (1963).

7 Gueguen, E., Hartenstein, J., Fricke-Begemann, C. & K., T. Raw material challenges in refractory application in *Raw material challenges in refractory application in Mineral by-products and waste-ash, slag, dust and construction debris* (ed K. J. Thome-Kozmiensky) 489-501 (Verlag GmbH, 2014).

8 Glasstone, S. & Dolan, P. J. *The Effects of Nuclear Weapons*. 653 (US Dept of Defense, U.S. Government Printing Office, 1977).

9 Hermes, R. E., Strickfaden, W. B. & J. A new look at trinitite. *Nucl Weap J* **2**, 2-7 (2005).

10 Thy, P., Willcox, G., Barfod, G. H. & Fuller, D. Q. Anthropogenic origin of siliceous scoria droplets from Pleistocene and Holocene archaeological sites in northern Syria. *J Archaeol Sci* **54**, 193-209 (2015).

11 Firestone, R. B. *et al.* Evidence for an extraterrestrial impact 12,900 years ago that contributed to the megafaunal extinctions and the Younger Dryas cooling. *Proc Nat Acad Sci* **104**, 16016-16021 (2007).

12 Kennett, D. J. *et al.* Wildfire and abrupt ecosystem disruption on California's Northern Channel Islands at the Ållerød–Younger Dryas boundary (13.0–12.9 ka). *Quat Sci Rev* **27**, 2530-2545 (2008).

13 Scott, A. C. *et al.* Fungus, not comet or catastrophe, accounts for carbonaceous spherules in the Younger Dryas "impact layer". *Geophys Res Lett* **37**, 1-5 (2010).

14 Pinter, N. *et al.* The Younger Dryas impact hypothesis: A requiem. *Earth Sci Rev* **106**, 247-264 (2011).

15 van Hoesel, A. *et al.* Nanodiamonds and wildfire evidence in the Usselo horizon postdate the Allerød-Younger Dryas boundary. *Proc Nat Acad Sci* **109**, 7648-7653 (2012).

16 Braadbaart, F. & Poole, I. Morphological, chemical and physical changes during charcoalification of wood and its relevance to archaeological contexts. *J Archaeol Sci* **35**, 2434-2445 (2008).

17 ICCP. The new inertinite classification (ICCP System 1994). *Fuel* **80**, 459-471 (2001).

18 Jones, T. P., Scott, A. C. & Cope, M. Reflectance measurements and the temperature of formation of modern charcoals and implications for studies of fusain. *Bull Soc Géol France* **162**, 193-200 (1991).

19 McParland, L. C., Collinson, M. E., Scott, A. C. & Campbell, G. The use of reflectance values for the interpretation of natural and anthropogenic charcoal assemblages. *AAS* **1**, 249-261 (2009).

20 Ascough, P. L. Charcoal reflectance measurements: implications for structural characterization and assessment of diagenetic alteration. *J Archaeol Sci* **37**, 1590-1599 (2010).
21 Kwiecińska, B. & Petersen, H. I. Graphite, semi-graphite, natural coke, and natural char classification-ICCP system. *Int J Coal Geol* **57**, 99-116 (2004).
22 Petersen, H. I. Morphology, formation and paleo-environmental implications of naturally formed char particles in coals and carbonaceous mudstones. *Fuel* **77**, 1177-1183 (1998).
23 Wittke, J. H. *et al.* Evidence for deposition of 10 million tonnes of impact spherules across four continents 12,800 y ago. *Proc Nat Acad Sci* **110**, E2088-E2097 (2013).
24 Guo, Bustin & R, M. FTIR spectroscopy and reflectance of modern charcoals and fungal decayed woods: implications for studies of inertinite in coals. *Int J Coal Geol* **37**, 29-53 (1998).
25 Schultz, P. H., Harris, R. S., Clemett, S. J., Thomas-Keprta, K. L. & Zárate, M. Preserved Flora and Organics in Impact Melt Breccias. *Geology* **42**, 515-518 (2014).
26 Florenskiy, K. Preliminary results from the 1961 combined Tunguska meteorite expedition *Meteoritica* **XXIII**, 3-37 (1965).
27 French, B. M. *Traces of Catastrophe: A Handbook of Shock-Metamorphic Effects in Terrestrial Meteorite Impact Structures.*, 120 (Lunar and Planetary Institute, 1998).
28 Chao, E. C. Shock effects in certain rock-forming minerals. *Science* **156**, 192-202 (1967).
29 Osinski, G. F., Bunch, T. E. & Wittke, J. Evidence for shock melting of carbonates from Meteor Crater, Arizona. *66th Annual Meeting Meteoritical society* (2003).
30 Wasson, J. T. Large aerial bursts: an important class of terrestrial accretionary events. *Astrobiology* **3**, 163-179 (2003).
31 Osinski, G. R. *et al.* The Dakhleh Glass: product of an impact airburst or cratering event in the Western Desert of Egypt? *Meteorit Planet Sci* **43**, 2089-2106 (2008).
32 Collins, G. S., Melosh, H. J. & Marcus, R. Earth impact effects program: a web-based computer program for calculating the regional environmental consequences of a meteoroid impact on Earth. *Meteorit Planet Sci* **40**, 817-840 (2005).
33 Pechersky, D., Markov, G. & Tsel'movich, V. Pure iron and other magnetic minerals in meteorites. *Solar System Research* **49**, 61-71 (2015).
34 Harris, R. & Schultz, P. When Rubble Piles Attack: The Menagerie of Microscopic Meteorite Debris in Pica Impact Glass. *Lunar and Planetary Science Conference* **50** (2019).
35 Beran, A. & Koeberl, C. Water in tektites and impact glasses by Fourier-transformed infrared spectrometry. *Meteorit Planet Sci* **32** (1997).
36 Gilchrist, J., Thorpe, A. N. & Senftle, F. E. Infrared analysis of water in tektites and other glasses. *J Geophys Res* **74**, 1475-1483 (1969).
37 Heide, K. & Heide, G. Vitreous state in nature—Origin and properties. *Chem Erde* **71**, 305-335 (2011).
38 Watt, N., Bouchet, R. A. & Lee, C. T. A. Exploration of tektite formation processes through water and metal content measurements. *Meteorit Planet Sci* **46**, 1025-1032 (2011).
39 Glass, B. P., Senftle, F. E., Muenow, D. W., Aggrey, K. E. & Thorpe, A. N. Atomic bomb glass beads: tektite and microtektite analogs. *Proc 2nd Intl Conf Natural Glasses Prague pp*, 361-369 (1987).
40 Bouska, V. *Natural Glasses*. 354 (Ellis Horwood, 1993).
41 Lowenstern, J. B. & Pitcher, B. W. Analysis of H2O in silicate glass using attenuated total (ATR) micro-FTIR spectroscopy. *Amer Minerol* **98**, 1660-1668 (2013).
42 Lowenstern, J. B., Bleick, H., Vazquez, J. A., Castro, J. M. & Larson, P. Degassing of Cl, F, Li, and Be during extrusion and crystallization of the lava dome at Volcan Chaiten, Chile during 2008 and 2009. *Bull Volcanol* **74**, 2303-2319 (2012).

43 Dixon, J. E., Stolper, E. M. & Delaney, J. R. Infrared spectroscopic measurements of CO2 and H2O glasses in the Juan de Fuca Ridge basaltic glasses. *Earth Planet Sci Lett* **90**, 87-104 (1988).

44 Koeberl, C. Geochemistry and origin of Muong Nong-type tektites. *Geochim Cosmochim Acta* **56**, 1033-1064 (1992).

45 Koeberl, C. & Sigurdsson, H. Geochemistry of impact glasses from the K/T boundary in Haiti - Relation to smectites and a new type of glass. *Geochim Cosmochim Acta* **56**, 2113-2129 (1992).

46 Rutherford, M. J. & Devine, J. D. I. Magmatic Conditions and Processes in the Storage Zone of the 2004-2006 Mount St Helens Dacite in *Magmatic Conditions and Processes in the Storage Zone of the 2004-2006 Mount St Helens Dacite in A volcano rekindled: the renewed eruption of Mount St. Helens, 2004-2006* (eds D.R. Sherrod, W.E. Scott, & P.H. Stauffer) Ch. 31, (US Geological Survey, 2008).

47 Sisson, T. W. & Vallance, J. W. Frequent eruptions of Mount Rainier over the last ~600 years. *Bull Volcanol* **71**, 595-618 (2009).

48 Thy, P., Segobye, A. K. & Ming, D. W. Implications of prehistoric glassy biomass slag from east-central Botswana. *J Archaeol Sci* **22**, 629-637 (1995).

49 Jacobson, L., Loock, J. C., van Huffman, T. N. & Dreyer, J. J. B. The occurrence of vitrified dung from the Kamdeboo district, southern Karoo, and Den Staat, Limpopo Valley, South Africa. *S Afr J Sci* **99**, 26-28 (2003).

50 Sterpenich, J. & Libourel, G. Water diffusion in silicate glasses under natural weathering conditions: evidence from buried medieval stained glasses. *J Non Cryst Solids* **352**, 5446-5451 (2006).

51 Takata, M. *et al.* Effect of Water Content on Properties of Na2O, 3SiO2 Glasses. *J Phys Colloques* **43**, C9-455-C459-458 (1982).

52 Zajacz, Z., Halter, W., Malfait, W. J., Bachmann, O. & Bodnar, R. J. A composition-independent quantitative determination of the water content in silicate glasses and silicate melt inclusions by confocal Raman spectroscopy. *Contrib Mineral Petrol* **150**, 631-642 (2005).

53 Gattacceca, J., Lamali, A., Rochette, P., Boustie, M. & Berthe, L. The effects of explosive-driven shocks on the natural remanent magnetization and the magnetic properties of rocks. *Phys Earth Planet Inter* **1**, 1-30 (2007).

54 Essene, E. & Fisher, D. Lightning strike fusion: extreme reduction and metal-silicate liquid immiscibility. *Science* **234**, 189-193 (1986).

55 Hopkins, R. P. *The Historiography of the Allied Bombing Campaign of Germany* MA thesis, East Tennessee State University, (2008).

56 Jenniskens, P. Mostly dormant comets and their disintegration into meteoroid streams: A Review. *EM&P* **102**, 505-520 (2008).

57 Napier, W. The hazard from fragmenting comets. *Monthly Notices of the Royal Astronomical Society* **488**, 1822-1827 (2019).

58 Napier, W. M. Palaeolithic extinctions and the Taurid Complex. *Mon Not R Astron Soc* **405**, 1901-1906 (2010).

59 Clube, S. & Napier, W. The microstructure of terrestrial catastrophism. *Mon Not R Astron Soc* **211**, 953-968 (1984).

60 Steel, D. & Asher, D. The orbital dispersion of the macroscopic Taurid objects. *Mon Not R Astron Soc* **280**, 806-822 (1996).

61 ASTM. *Standard Test Method D7708: Microscopical determination of the reflectance of vitrinite dispersed in sedimentary rocks*. (ASTM International, 2014).

62 Paterson, M. S. The determination of hydroxyl by infrared absorption in quartz, silicate glasses and similar materials. *Bull Minéral* **105**, 20-29 (1982).

63 Wasilewski, P. Magnetization of small iron-nickel spheres. *Phys Earth Planet Inter* **26**, 149-161 (1981).

64 Wasilewski, P. Magnetic and microstructural properties of some lodestones. *Phys Earth Planet Inter* **15**, 349-362 (1977).

65 Wasilewski, P. & Kletetschka, G. Lodestone: Natures only permanent magnet-What it is and how it gets charged. *Geophys Res Lett* **26**, 2275-2278 (1999).

66 Parry, L. G. Magnetization of immobilized particle dispersion with two distinct particle sizes. *Phys Earth Planet Inter* **28**, 0-241 (1982).

67 Lewis, M. Some experiments on synthetic titanomagnetites. *Geophys J Int* **16**, 295-310 (1968).

68 Kletetschka, G., Kohout, T. & Wasilewski, P. J. Magnetic remanence in the Murchison meteorite. *Meteorit Planet Sci* **38**, 399-405 (2003).

69 Harris, R., Schultz, P. & King, P. in *Bridging the Gap II: Effect of Target Properties on the Impact Cratering Process.* 57-58.
